# Supplementary material for: Evolutionary safety of lethal mutagenesis driven by antiviral treatment
Source: PLoS Biol. 2023 Aug 8;21(8):e3002214. doi: 10.1371/journal.pbio.3002214 (PMC10409280; doi:10.1371/journal.pbio.3002214)
Supplement: S1 Text — Supplementary figures: Figs A1 to A31, and supplementary tables: Tables A1 to A4. (DOCX) [file pbio.3002214.s001.docx]

S1 Text: Supplementary Figures and Tables

Evolutionary safety of lethal mutagenesis driven by antiviral treatment

Gabriela Lobinska^1^, Yitzhak Pilpel^1^*, Martin A Nowak^2^*

1 Department of Molecular Genetics, Weizmann Institute of Science, Rehovot 76100, Israel

2 Department of Mathematics, Department of Organismic and Evolutionary Biology, Harvard University, Cambridge MA 02138, USA

*corresponding authors

**
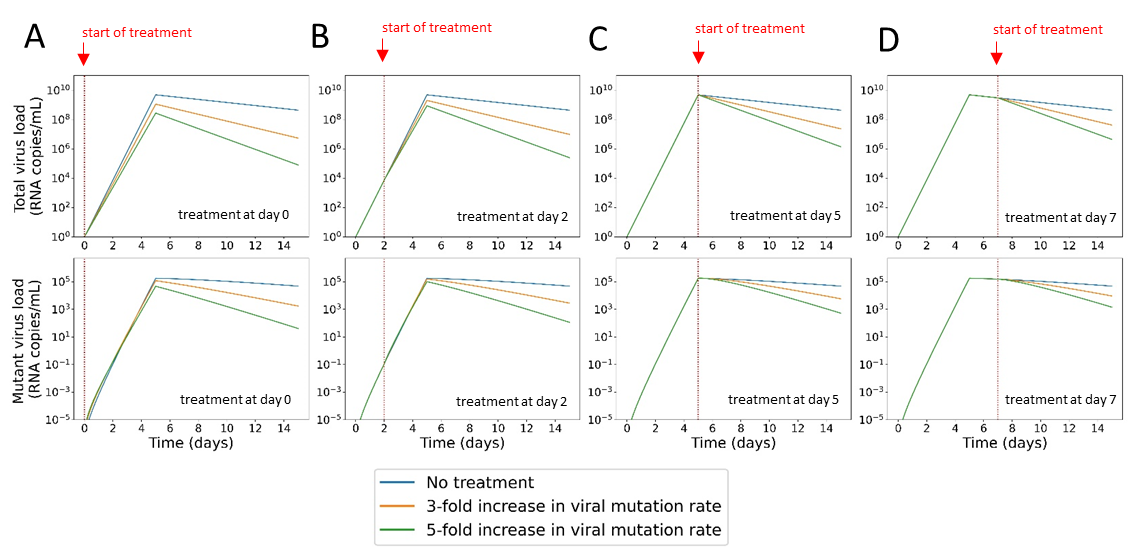
**

**Fig A1. Time series of total virus (**$\boldsymbol{v=x+y}$**) and mutant virus (**$\boldsymbol{y}$**) with and without treatment.** We consider four starting points for treatment: (A) at infection, day 0; (B) before reaching peak virus load, day 2; (C) at peak virus load, day 5; and (D) after peak virus load, day 7. The red arrow and dotted line indicate the beginning of treatment. Virus load increases during the first 5 days when the death rate is $a_{0}$. Virus load subsequently declines when the death rate is $a_{1}$. Treatment with a mutagenic drug reduces the total abundance of virus. The higher the mutation rate induced by treatment, the higher is the decrease in virus load. The abundance of mutant virus (here, we are interested in a specific mutant) can increase transiently after the start of treatment, but subsequently declines compared to the case of no treatment. Parameters: $a_{0}=3$ per day, $a_{1}=7.7$ per day, $b=7.61$ per day, $u_{0}={10}^{-6}$ per bp, $m=20,000$ positions, $n=1$ position. Initial condition: $x_{0}=1$ and $y_{0}=0$. The code used to generate this figure can be found at DOI: 10.5281/zenodo.8017992.


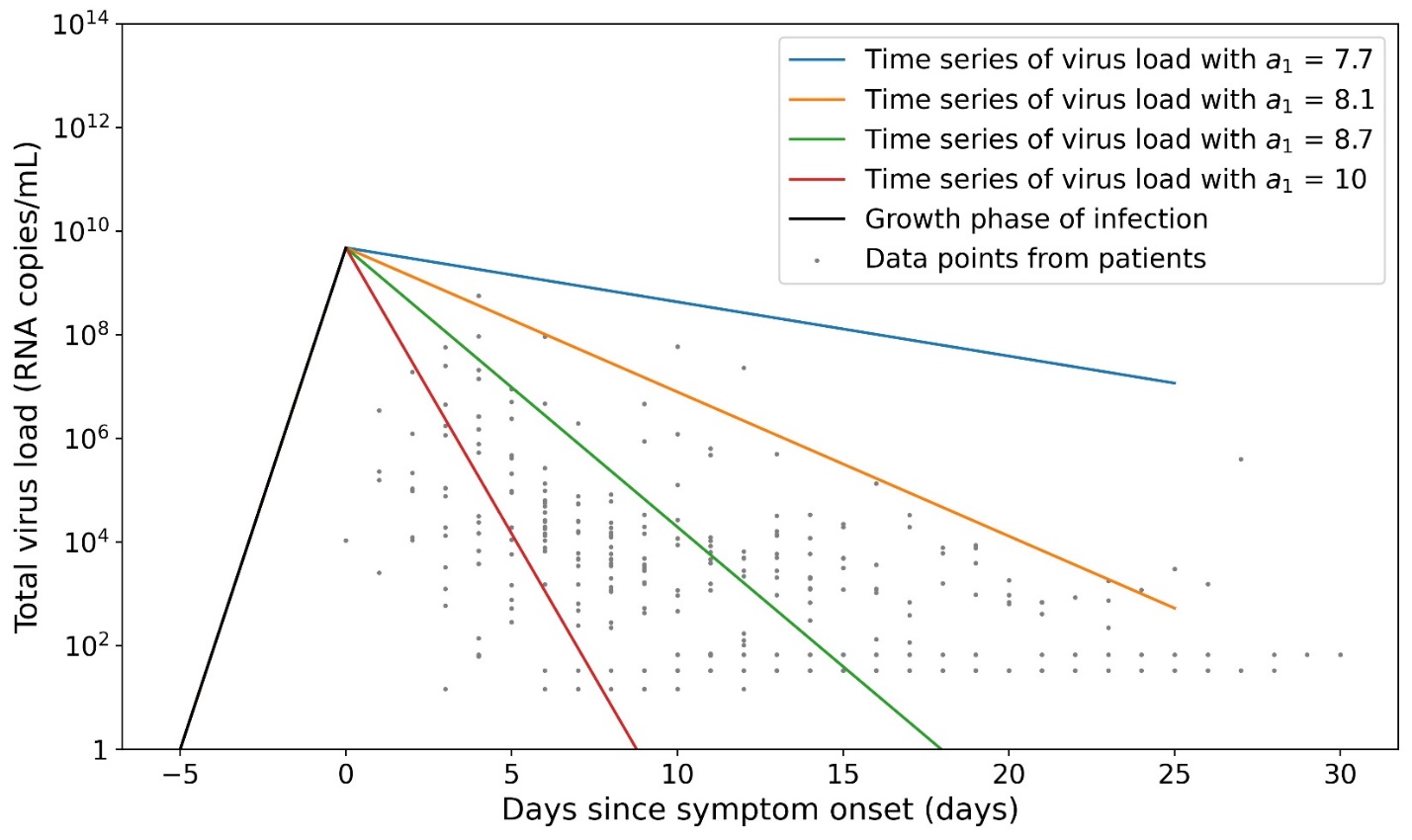


**Fig A2. Comparison of time series of virus load with empirical data points.** We compare virus load measurements for individual patients - taken from Ref [1], who pooled it from Refs[2–5] - with time series plotted with Eq. 1. The blue line corresponds to our upper bound estimate of $a_{1}$, the virus death rate during the clearance phase. The red line corresponds to our lower bound estimate of $a_{1}$. We observe that the majority of points falls between the two lines, confirming that our estimates are realistic. Initial condition: $x_{0}=1$ and $y_{0}=0$. Parameters: $b=7.61$ per day, $a_{0}=3$ per day, $T=5$ days, $m=20,000$ positions. The code used to generate this figure can be found at DOI: 10.5281/zenodo.8017992.


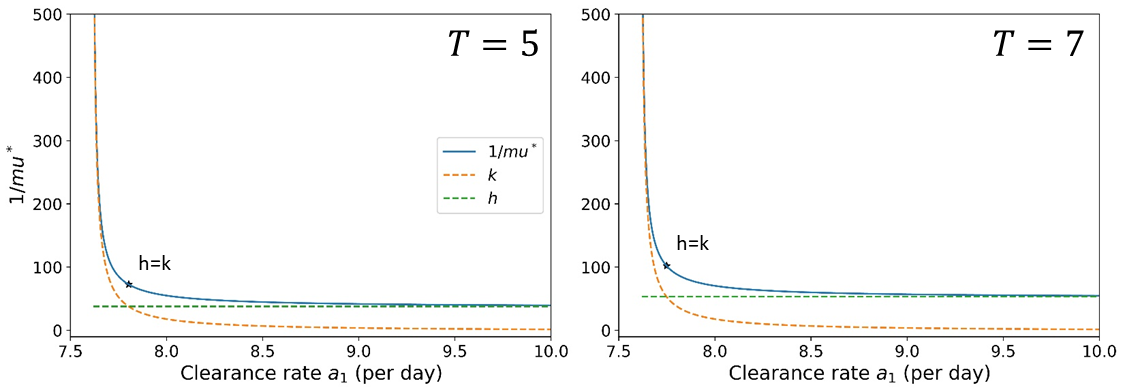


**Fig A3. Validity of approximations for** $\boldsymbol{u}^{\boldsymbol{*}}$ **when treatment starts at infection.** The cumulative mutant virus load, $Y(u)$, is a one humped function which attains a maximum at mutation rate $u^{*}$. The figure shows the value of $1/mu^{*}$as function of $a_{1}.$ We use the notation $h=bT$ and $k=[b\left( 2b-a_{0}-a_{1} \right)]/[\left( b-a_{0} \right)\left( a_{1}-b \right)]$. If $h\ll k$ then $1/mu^{*}\approx h$. If $k\ll h$ then $1/mu^{*}\approx k$. If $h\approx k$ then $1/mu^{*}\approx h/0.52138$. We observe good agreement. Other parameters: $b=7.61$ per day, $a_{0}=3$ per day, $m=20,000$ positions, $n=1$ position. The code used to generate this figure can be found at DOI: 10.5281/zenodo.8017992.


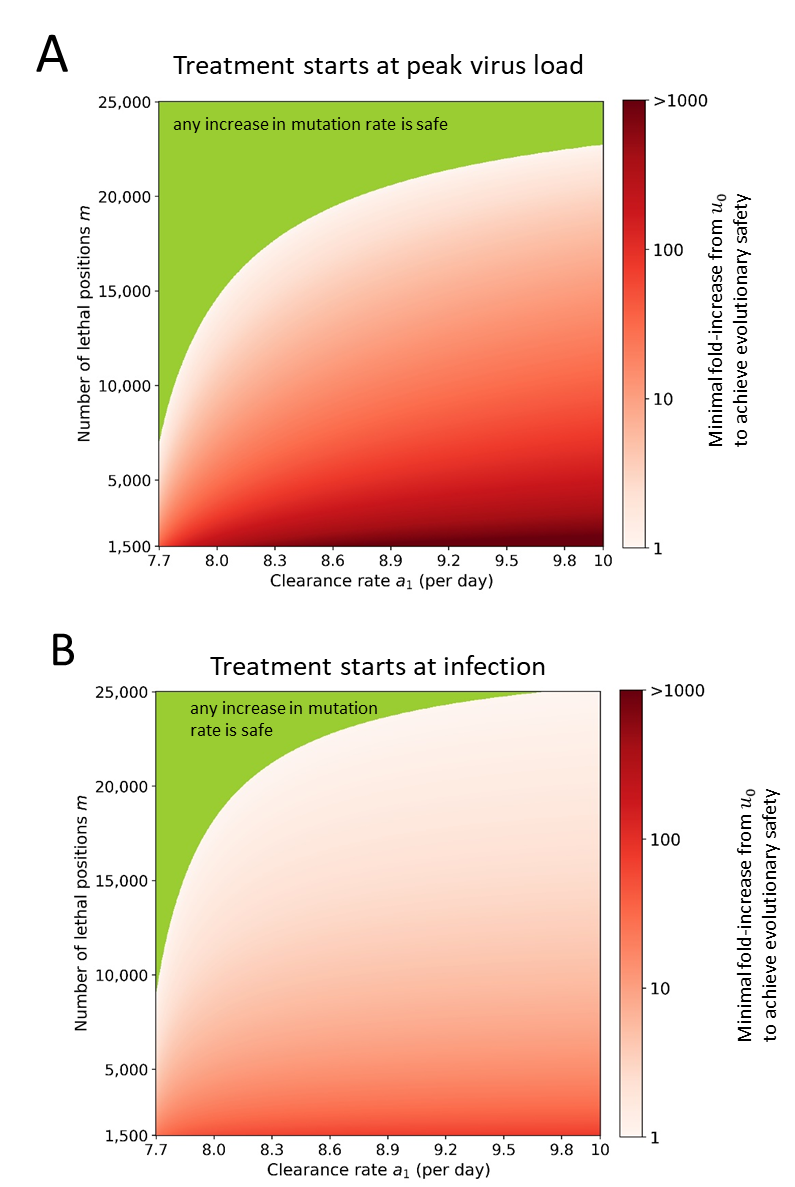


**Fig A4. Evolutionary safety of mutagenic treatment.** In the green parameter region, any increase in mutation rate reduces the cumulative mutant virus load and is therefore evolutionarily safe. In the red shaded region, we indicate the minimum fold increase in mutation rate that is required to reduce the cumulative mutant load. Contour lines for 3-fold and 10-fold increase are shown. (A) Treatment starts at peak virus load. (B) Treatment starts at infection. Parameters: $b=7.61$ per day, $a_{0}=3$ per day, $n=1$, $T=5$ days, $u_{0}={10}^{-6}$ per bp. The code used to generate this figure can be found at DOI: 10.5281/zenodo.8017992.

**
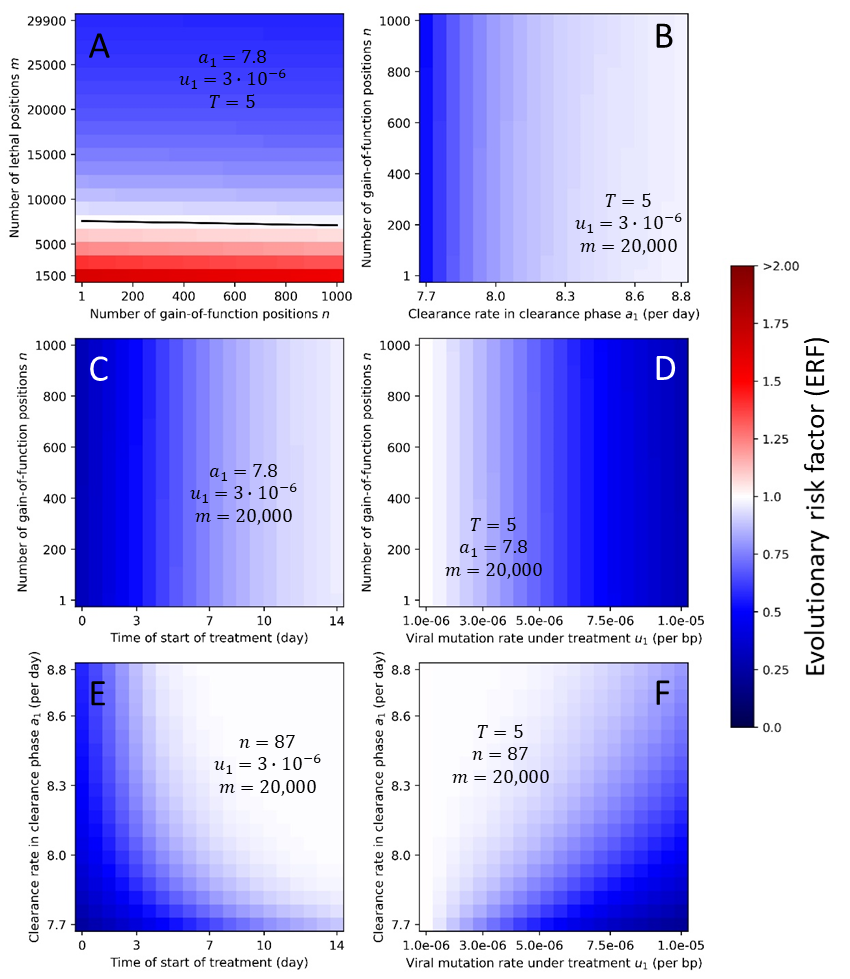
**

**Fig A5. Evolutionary risk factor for grids of selected parameters.** For each pair of parameters, we numerically compute the ERF for a range of values, while other parameters are fixed. Note that the value of $n$ has little effect on the ERF. ERFs above 1 are only observed for low values of the number of lethal positions $m$. The ERF decreases with early treatment, high viral mutation rate under treatment, large number of lethal positions. Initial condition: $x_{0}=1$ and $y_{0}=0$. The code used to generate this figure can be found at DOI: 10.5281/zenodo.8017992.


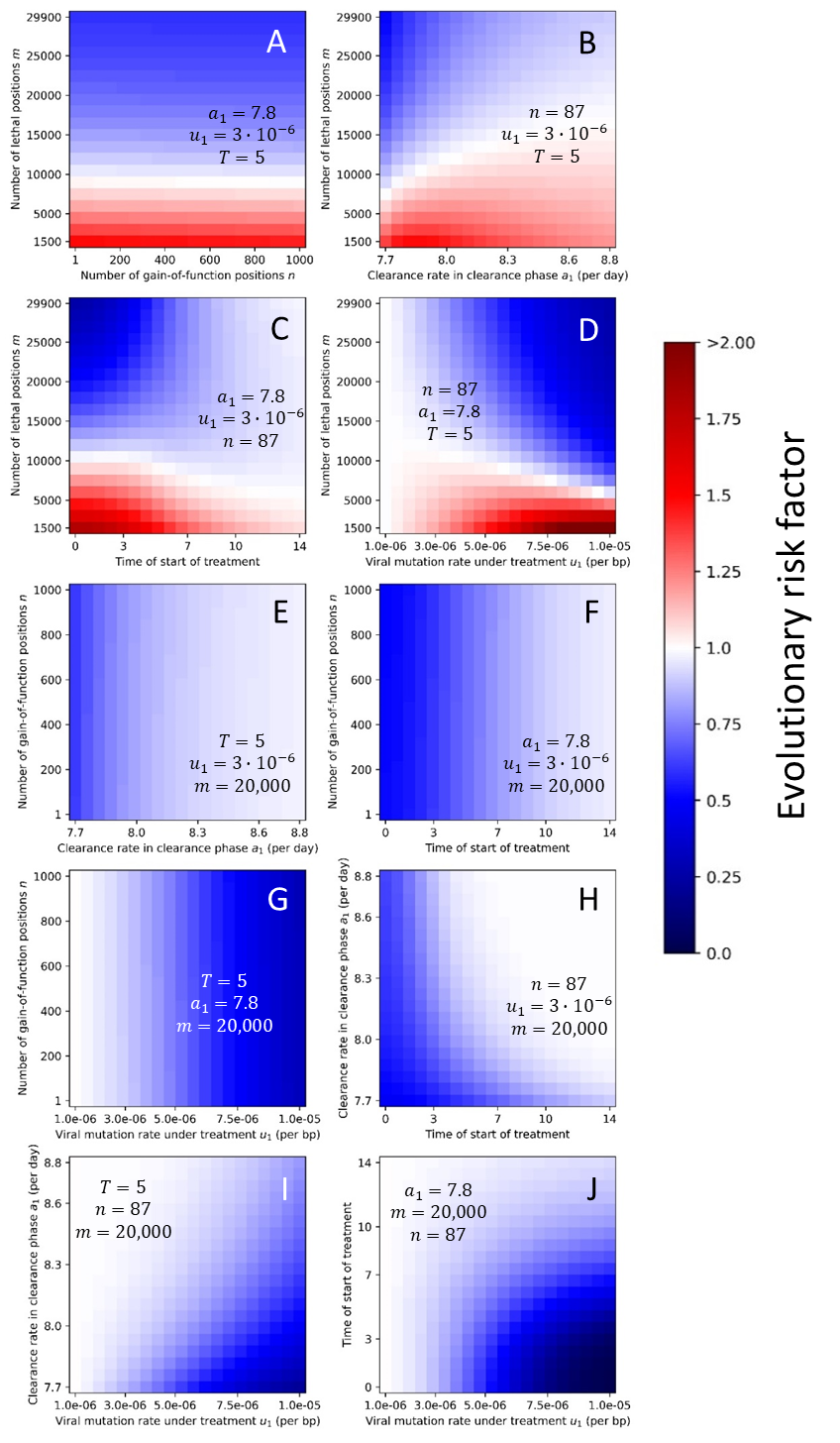


**Fig A6. Evolutionary risk factor for 5-day treatment starting at day 2.** For each pair of parameters, we numerically compute the ERF for a range of values, while other parameters are fixed. Treatment increases the virus mutation rate from $u_{0}$ to $u_{1}$ and starts at day 2 unless otherwise specified. Treatment is stopped after five days. We observe no major differences with Figure 5 and Supplementary Figure 3, where treatment continues until the end of infection. Initial condition: $x_{0}=1$ and $y_{0}=0$. Parameters: $b=7.61$ per day, $a_{0}=3$ per day, $T=5$ days. The code used to generate this figure can be found at DOI: 10.5281/zenodo.8017992.

**
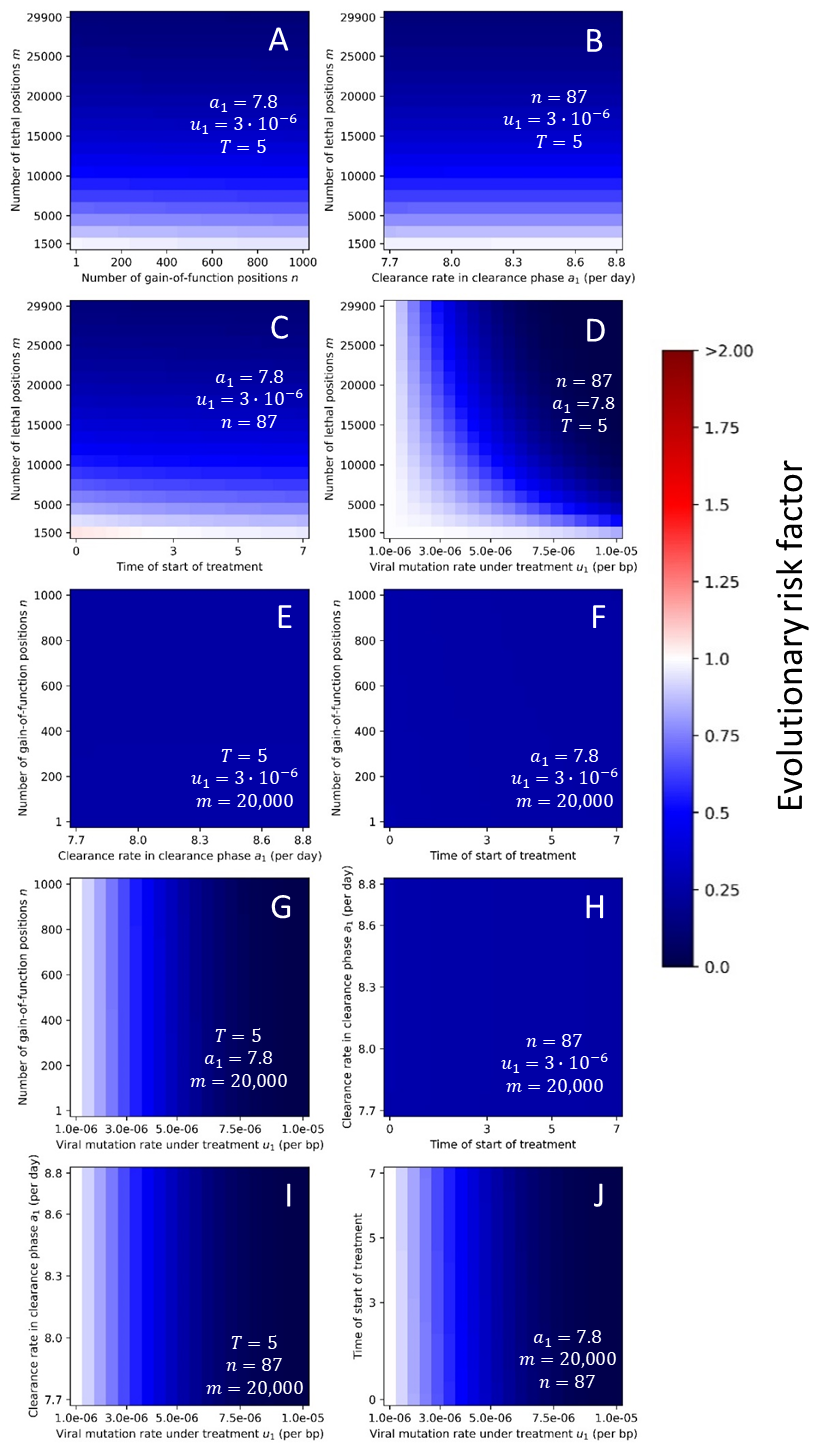
**

**Fig A7. Evolutionary risk factor for mutation rate** $\boldsymbol{u}_{\boldsymbol{0}}\boldsymbol{=5\cdot1}\boldsymbol{0}^{\boldsymbol{-6}}$**.** For each pair of parameters, we numerically compute the ERF for a range of values, while other parameters are fixed. We observe that many more regions of the parameter space are safe compared to $u_{0}={10}^{-6}$. Initial condition: $x_{0}=1$ and $y_{0}=0$. Parameters: $b=7.61$ per day, $a_{0}=3$ per day, $T=5$ days. The code used to generate this figure can be found at DOI: 10.5281/zenodo.8017992.


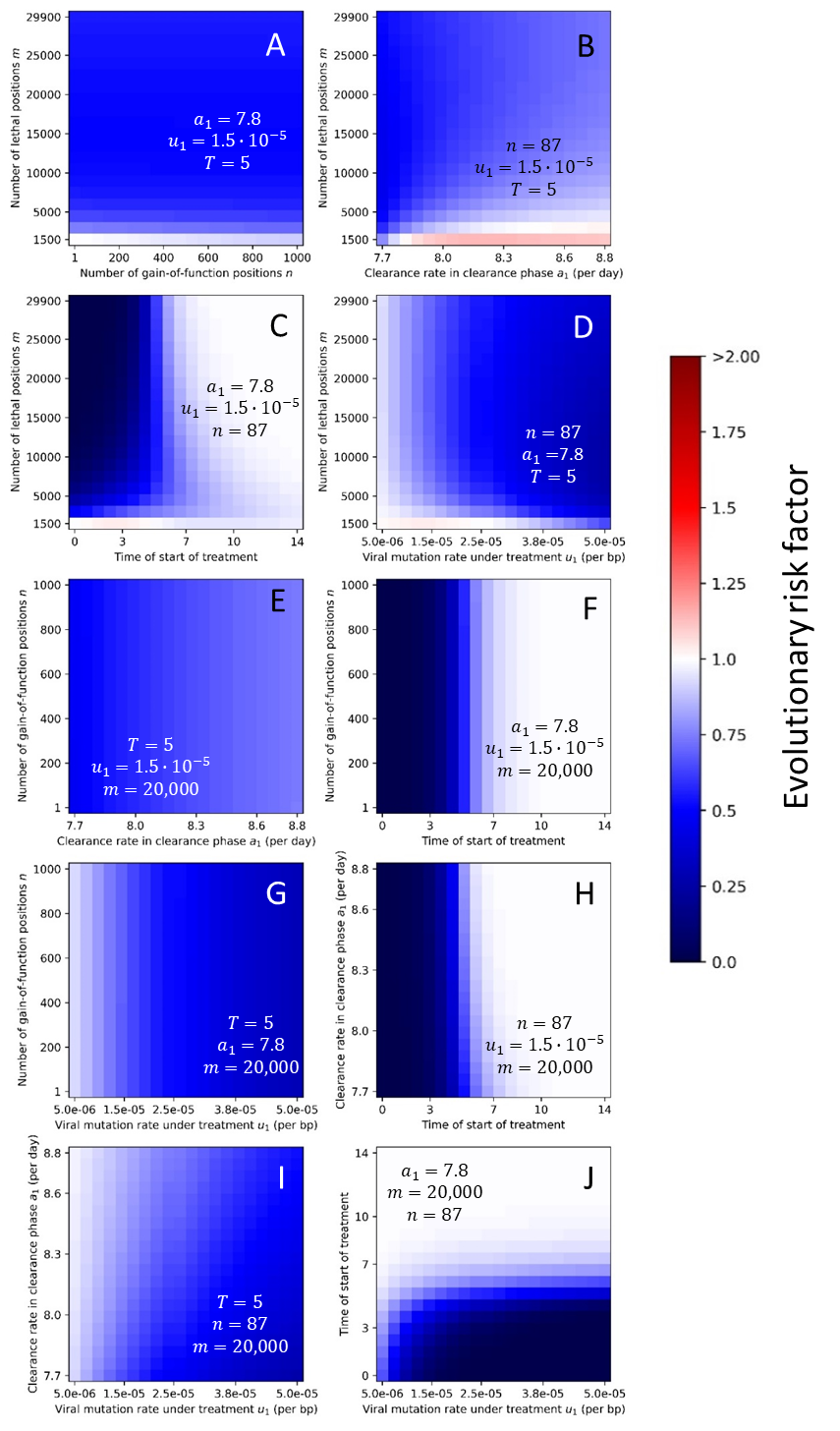


**Fig A8. Evolutionary risk factor for mutation rate** $\boldsymbol{u}_{\boldsymbol{0}}\boldsymbol{=1}\boldsymbol{0}^{\boldsymbol{-5}}$**.** For each pair of parameters, we numerically compute the ERF, while other parameters are fixed. We observe that many more regions of the parameter space are safe compared to $u_{0}={10}^{-6}$ or $u_{0}=5\cdot{10}^{-6}$. Initial condition: $x_{0}=1$ and $y_{0}=0$. Parameters: $b=7.61$ per day, $a_{0}=3$ per day, $T=5$ days. The code used to generate this figure can be found at DOI: 10.5281/zenodo.8017992.

**
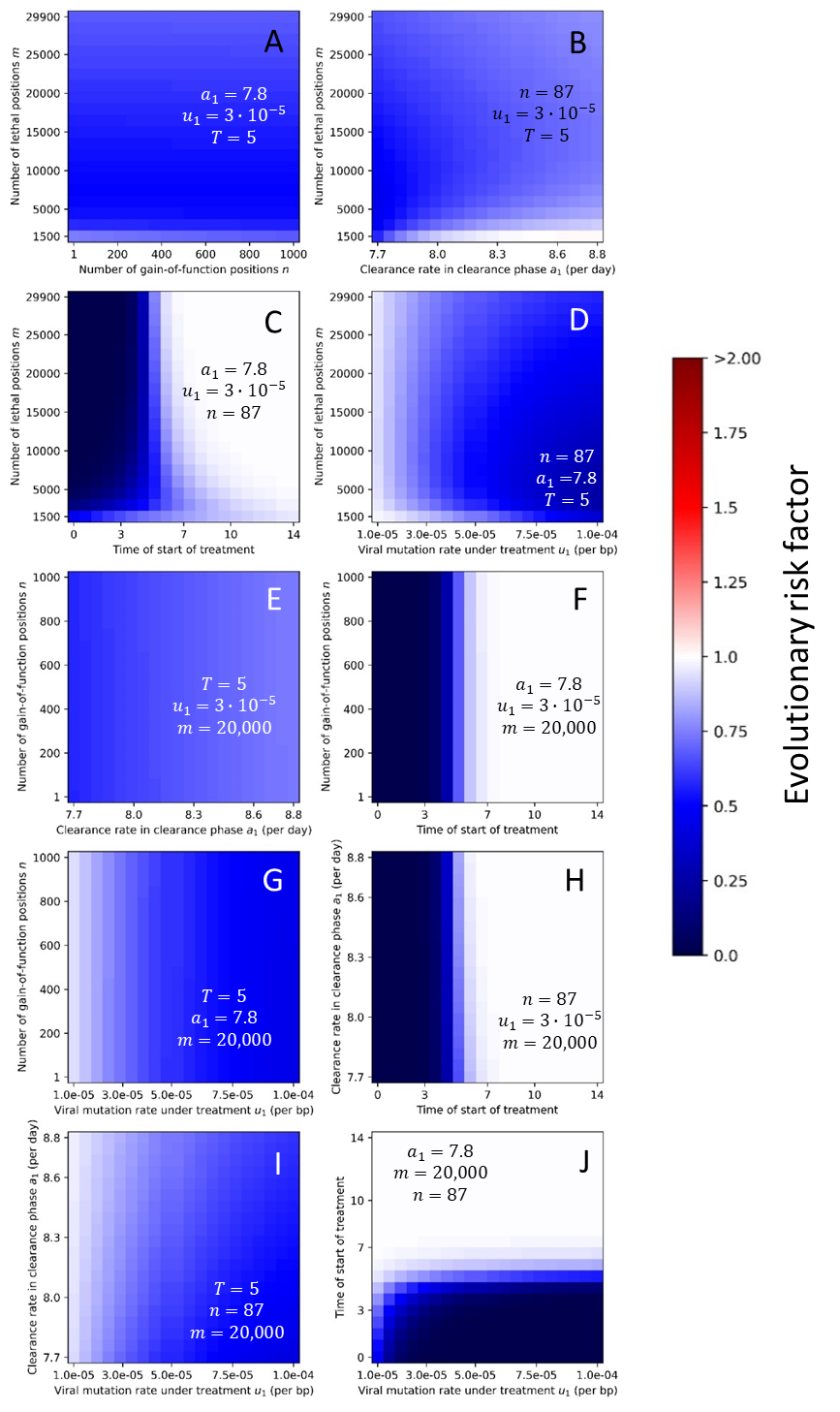
**

**Fig A9. Evolutionary risk factor for the virus load accumulated between the end of treatment and virus clearance.** For each pair of parameters, we numerically compute the ERF for a range of values, while other parameters are fixed. Treatment begins at peak virus load (5 days) and lasts for 5 days. We observe that for all parameter sets, the ERF is substantially below 1. This is because the treatment reduces the number of potential ancestors for the mutant virus, and hence has lasting effects on the ERF even after treatment has ceased. Initial condition: $x_{0}=1$ and $y_{0}=0$. Parameters: $b=7.61$ per day, $a_{0}=3$ per day, $u_{0}={10}^{-6}$ per bp, $T=5$ days. The code used to generate this figure can be found at DOI: 10.5281/zenodo.8017992.


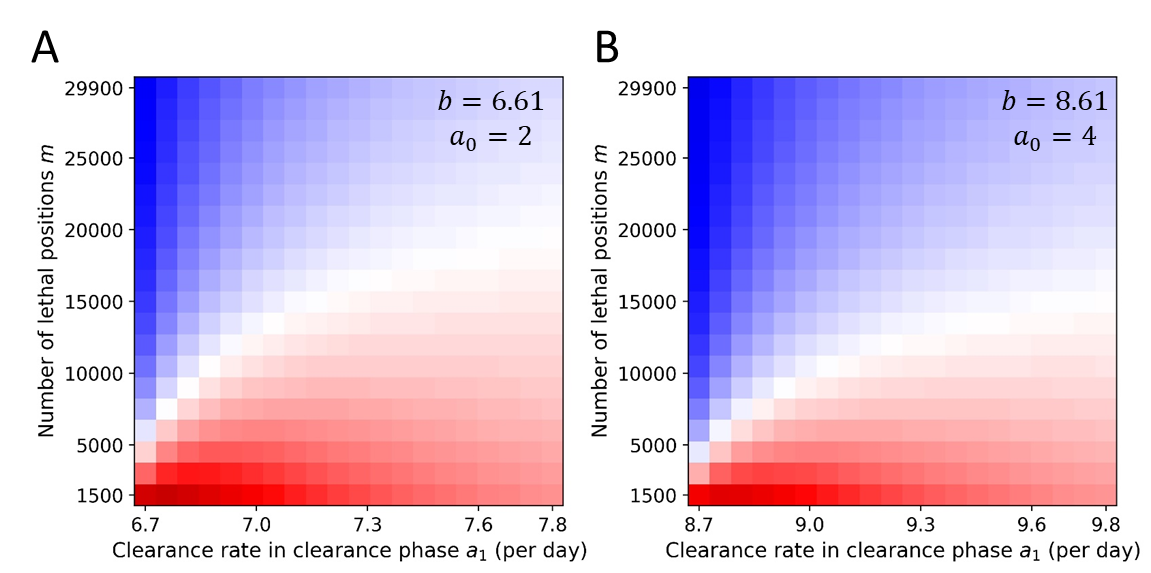


**Fig A10. Evolutionary risk factor (ERF) for other values of** $\boldsymbol{b}$ **and** $\boldsymbol{a}_{\boldsymbol{0}}$**.** Our estimates for viral birth and death rates in the growth phase are $b=7.61$ per day and $a_{0}=3$per day. Here we compute a parameter grid of ERF versus $m$ and $a_{1}$for two other choices of $b$ and $a_{0}$, which maintain the same net growth rate (ignoring lethal mutations). For $b=6.61$ per day and $a_{0}=2$ per day we observe slightly higher ERF values. For $b=8.61$ per day and $a_{0}=4$ per day we observe slightly lower ERF values. The code used to generate this figure can be found at DOI: 10.5281/zenodo.8017992.


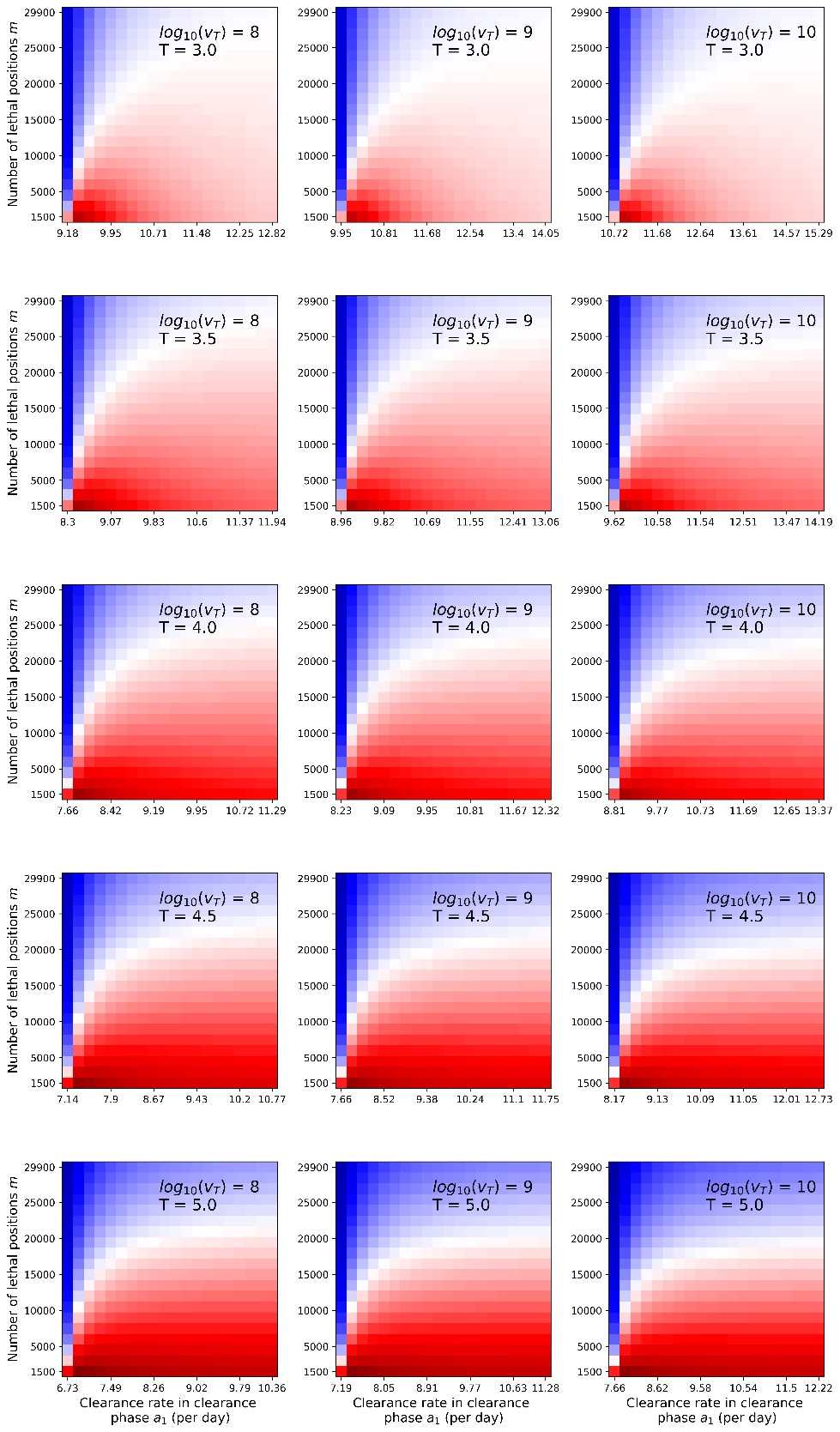


**Fig A11. Sensitivity analysis of the evolutionary risk factor on the time to virus peak and the virus load at peak.** We calculated the birth rate $b$ for each value of the peak of the virus load and the time to peak of the virus load. For each birth rate $b$, we adjusted the clearance rate in the clearance phase $a_{1}$ to reflect clearance times between 5 and 30 days. Parameters: $u_{0}={10}^{-6}$, $u_{1}=3\cdot{10}^{-6}$, $n=87$, $a_{0}=3$. Initial conditions: $x\left( 0 \right)=1$, $y\left( 0 \right)=0$. The code used to generate this figure can be found at DOI: 10.5281/zenodo.8017992.


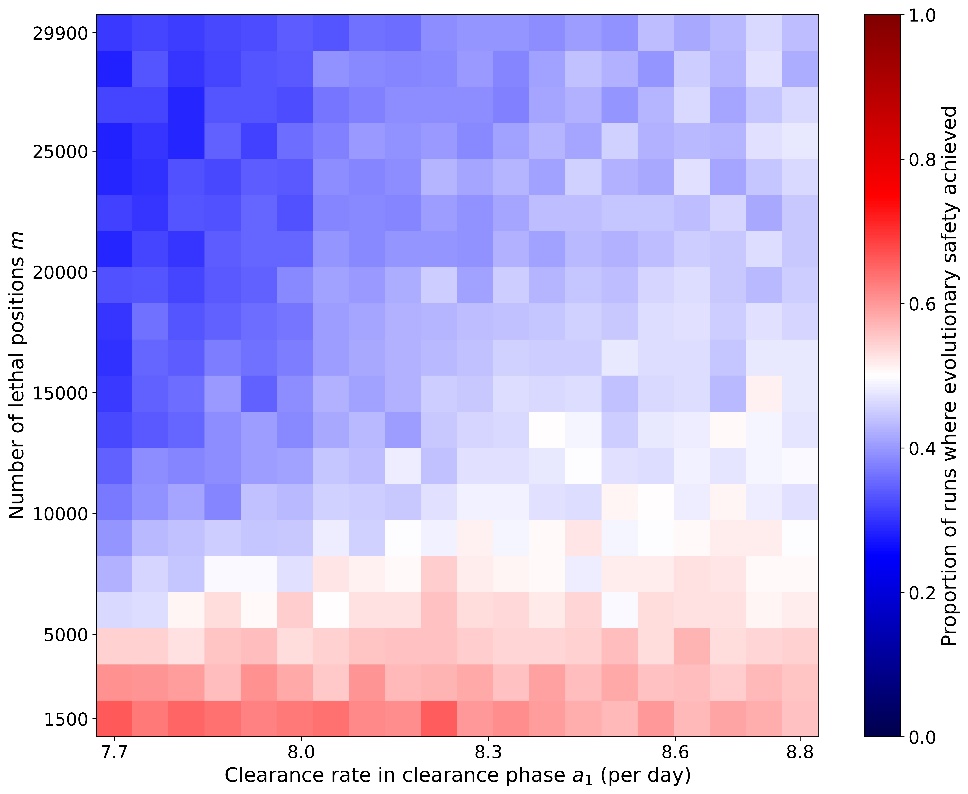


**Fig A12. Probability that evolutionary safety is achieved given a value of the number of lethal positions** $\boldsymbol{m}$ **and the clearance rate in clearance phase** $\boldsymbol{a}_{\mathbf{1}}$**.** For each pair of values, we ran 1000 runs of the Gillespie algorithm. The value plotted is the proportion of runs where the cumulative sum of the potentially concerning mutant with treatment was higher than the cumulative sum of the potentially concerning mutant without treatment, that is, the proportion of runs where the ERF exceeded 1. Treatment starts at peak of the virus load. Parameters: $b=7.61$, $a_{0}=3$, $n=87$, $T=5$, $u_{0}={10}^{-6}$, $u_{1}=3\cdot{10}^{-6}$. Initial condition: $x\left( 0 \right)=5$, $y\left( 0 \right)=0$. The code used to generate this figure can be found at DOI: 10.5281/zenodo.8017992.


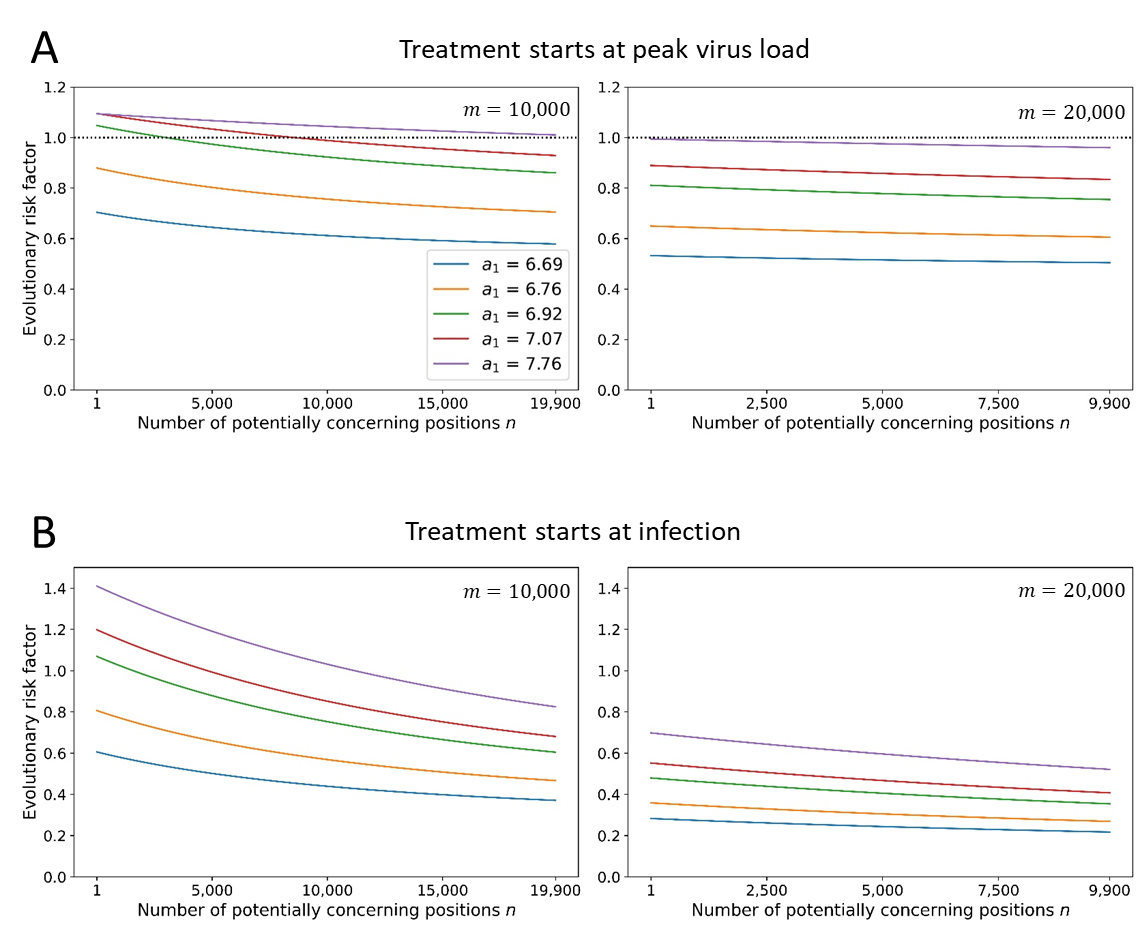


**Fig A13. Evolutionary risk factor versus the number of concerning (or viable) positions, *n* , for a lower value of birth rate,** $\boldsymbol{b}$**.** Here the ERF is slightly higher than for Figure 5, which uses b = 7.61 per day, but it is still a declining function of the number of positions, $n$. Parameters: $b=6.61$ per day, $a_{0}=2$ per day, $u_{0}={10}^{-6}$ per bp, $u_{1}=3\cdot{10}^{-6}$ per bp, $T=5$ days. Initial condition: $x_{0}=1$ and $y_{0}=0$. The code used to generate this figure can be found at DOI: 10.5281/zenodo.8017992.


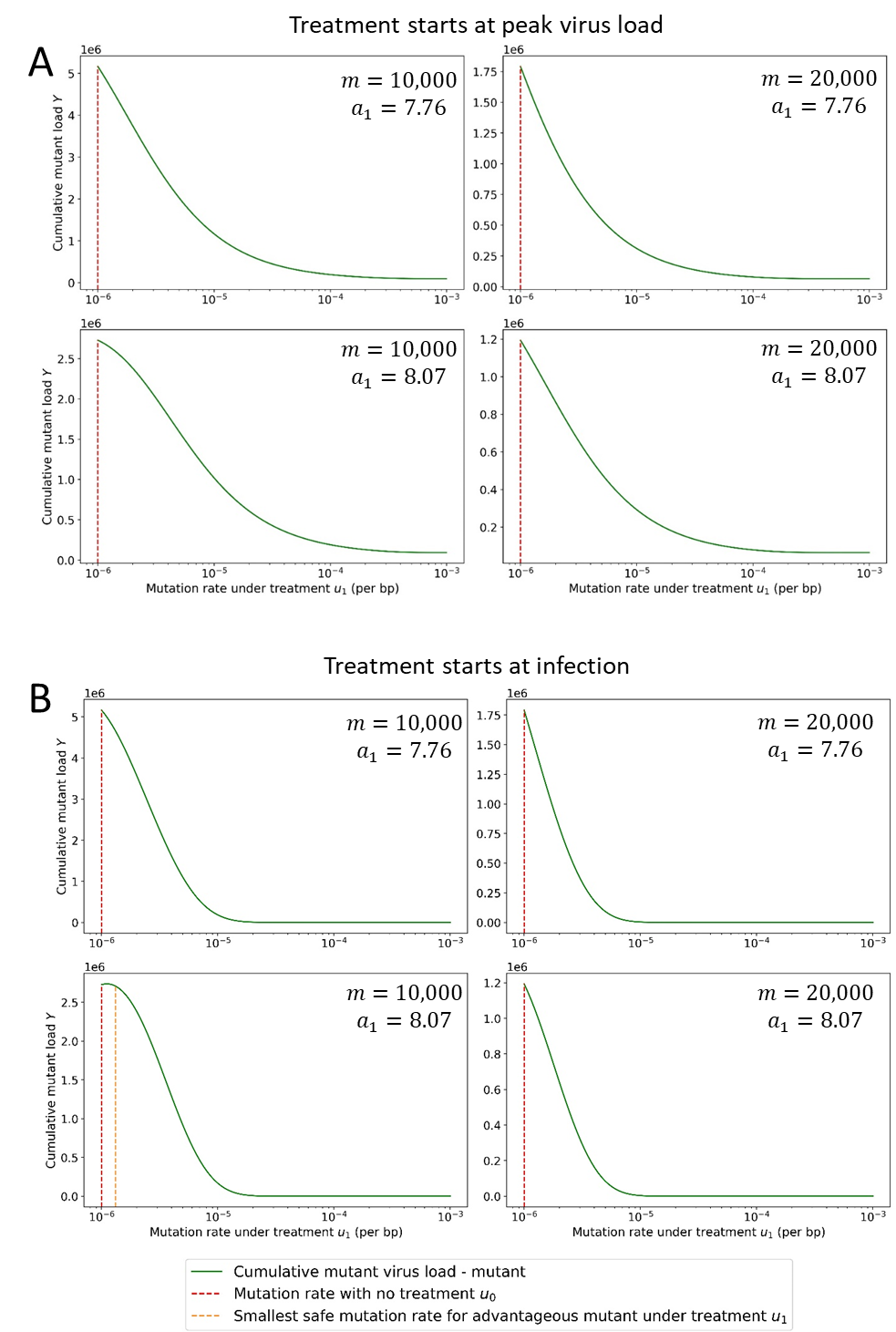


**Fig A14. Cumulative mutant load Y versus mutation rate** $\boldsymbol{u}_{\boldsymbol{1}}$ **for the case of an advantageous mutant.** We consider a concerning mutant with a 0.5% advantage in the birth rate. As expected, we observe a higher cumulative mutant load for the advantageous mutant (green line) compared to the neutral mutant (blue line). But the minimum mutation rate under treatment which is required for evolutionary safety is slightly lower for the advantageous mutant. Parameters: $b=7.61$, $b_{MT}=1.005\cdot b, a_{0}=3$, $n=1$, $T=5$, $m$and $a_{1}$ as shown. The code used to generate this figure can be found at DOI: 10.5281/zenodo.8017992.

**
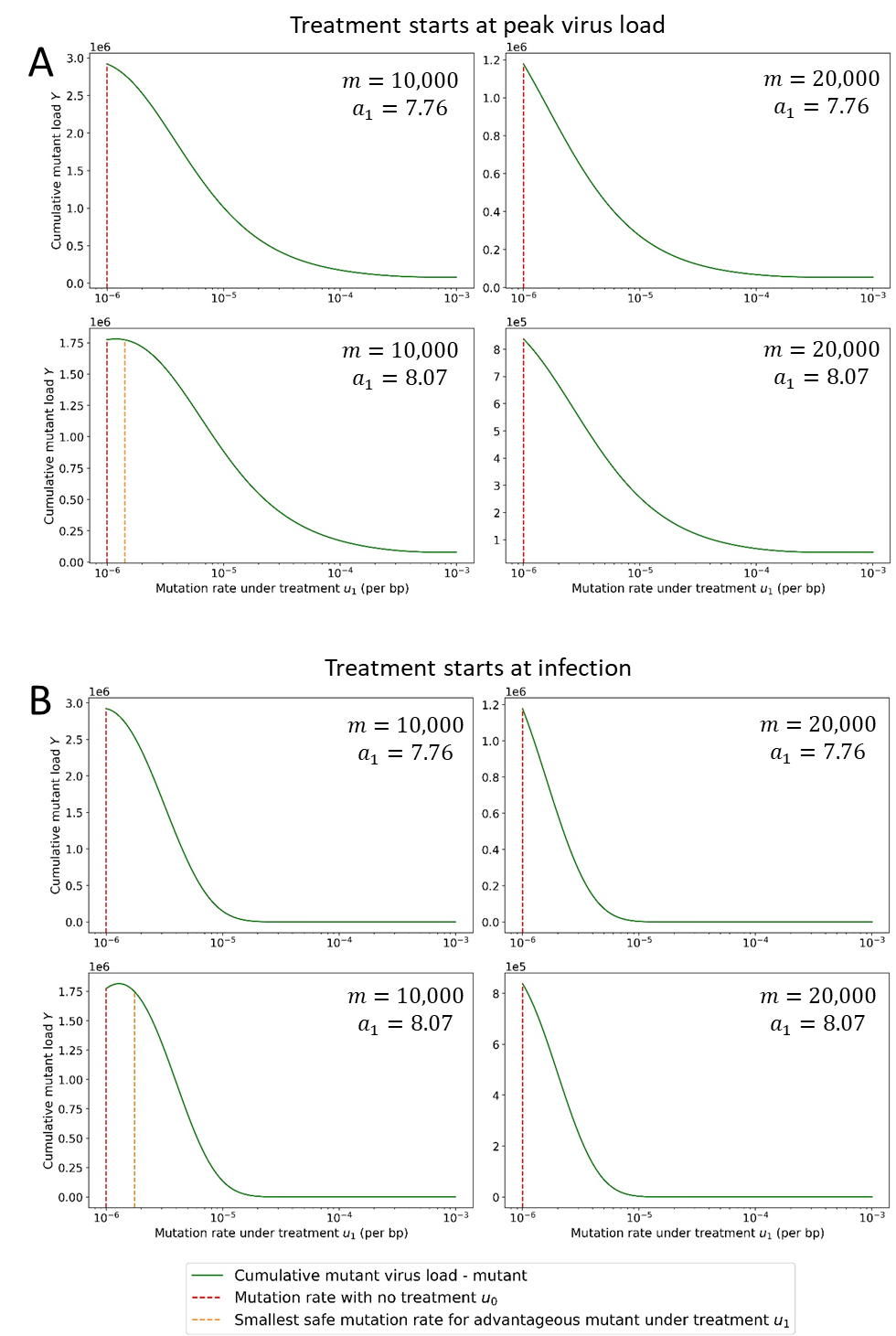
**

**Fig A15. Cumulative mutant load Y versus mutation rate** $\boldsymbol{u}_{\boldsymbol{1}}$ **for the case of a disadvantageous mutant.** Same as Supplementary Figure 12, except $b_{MT}=0.995\cdot b$. The code used to generate this figure can be found at DOI: 10.5281/zenodo.8017992.


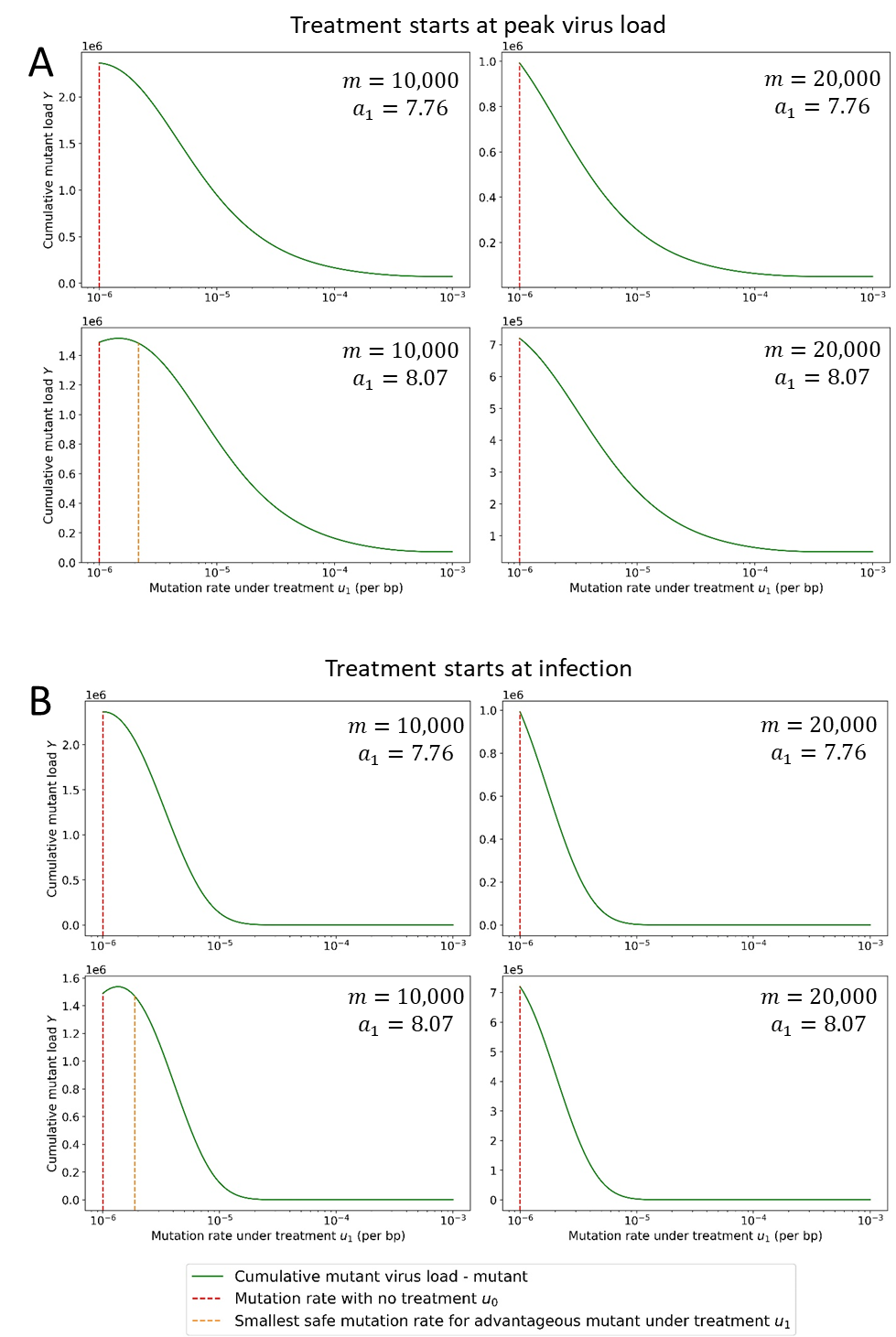


**Fig A16. Cumulative mutant load Y versus mutation rate** $\boldsymbol{u}_{\boldsymbol{1}}$ **for the case of a disadvantageous mutant.** Same as Supplementary Figure 12, except $b_{MT}=0.99\cdot b$. The code used to generate this figure can be found at DOI: 10.5281/zenodo.8017992.


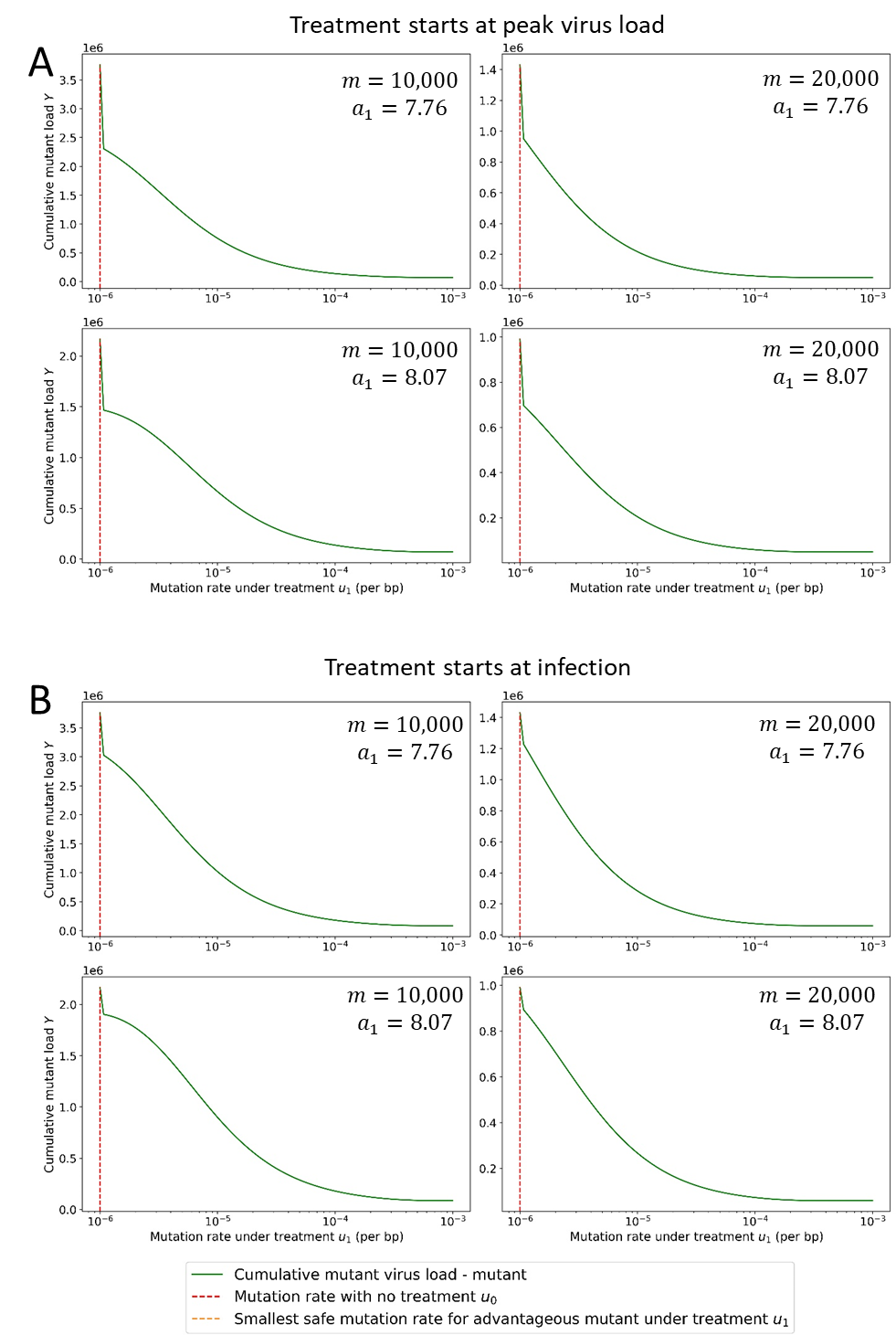


**Fig A17. Cumulative mutant load Y versus mutation rate** $\boldsymbol{u}_{\boldsymbol{1}}$ **for the case of a disadvantageous effect if the treatment only on the wild-type virus.** The wild-type virus only birth rate is decreased by 1% under treatment, that is when $u_{1}>u_{0}$. The code used to generate this figure can be found at DOI: 10.5281/zenodo.8017992.


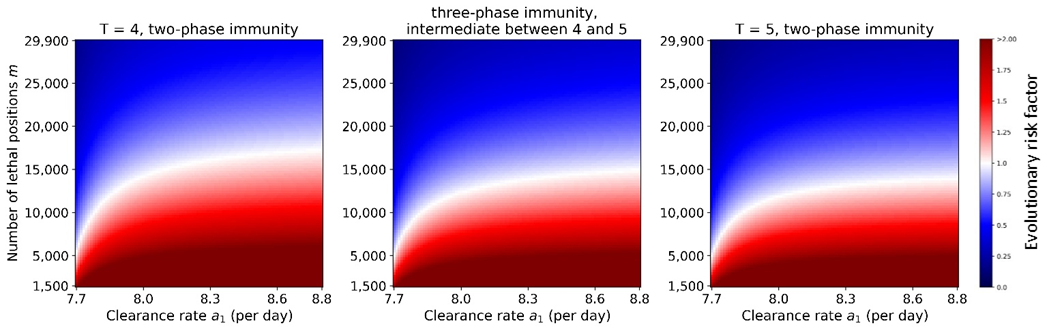


**Fig A18. Evolutionary risk factor versus the number of lethal positions,** $\boldsymbol{m,}$**and the clearance rate,** $\boldsymbol{a}_{\boldsymbol{1}}$**, for a three-phase immune response.** The values of ERF for a three-phase immunity scenario – where the clearance rate equals to the arithmetic average of $a_{0}$ and $a_{1}$ between days 4 and 5 – is bounded from below by the ERF values of two-phase immunity with T = 5 and is bounded from above by the ERF values of two-phase immunity with $T = 4$ days. Treatment starts at infection. Parameters: $n=1$ position, $u_{1}=3\cdot{10}^{-6}$ per bp. Initial condition: $x_{0}=1$ and $y_{0}=0$. The code used to generate this figure can be found at DOI: 10.5281/zenodo.8017992.


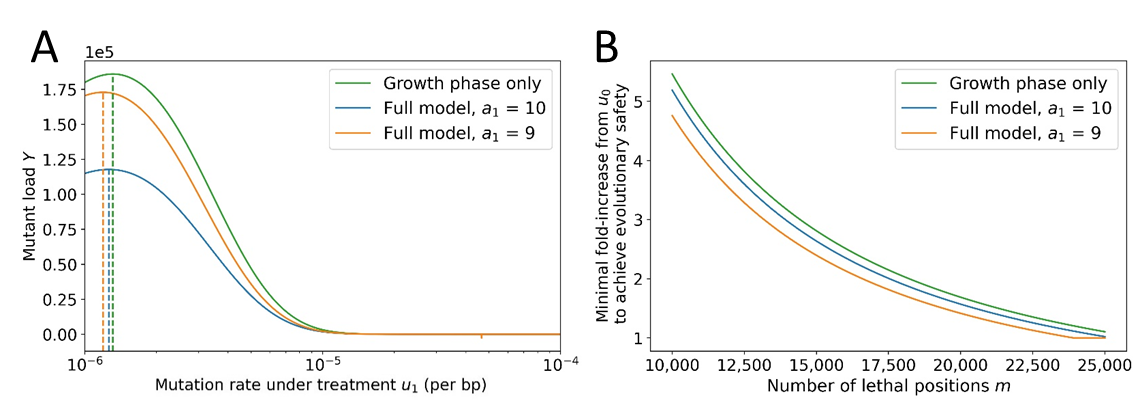


**Fig A19. Comparing the simplified and the full model.** In the simplified model, we only consider the growth phase of the virus, and we use for evaluation the abundance of mutant virus at time T. (A) Comparison between abundance of mutant virus at the end of the growth phase (green line) and the cumulative mutant virus load of the full model (orange and blue lines). The mutation rates at peak are indicated with a dashed line and are very close. (B) Minimum fold increase of mutation rate which treatment must induce to be evolutionarily safe. The simple model (green line) is a good approximation for the full model with fast clearance rates. Parameters: $b=7.61$ per day, $a_{0}=3$ per day, $n=1$, $T=5$ days, and $m=20,000$ positions. Initial condition: $x_{0}=1$ and $y_{0}=0$. The code used to generate this figure can be found at DOI: 10.5281/zenodo.8017992.


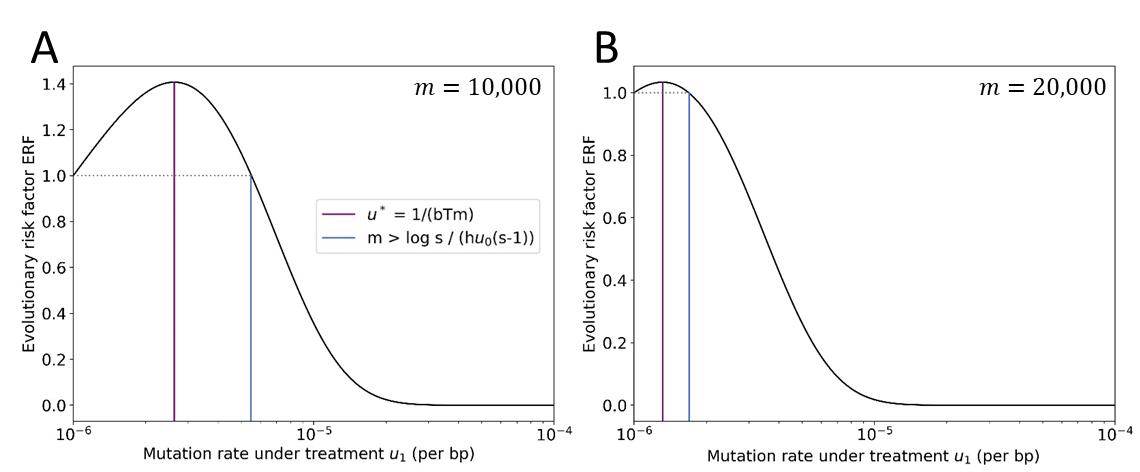


**Fig A20. Agreement between the analytical formulas for** $\boldsymbol{u}^{\boldsymbol{*}}$ **and for the minimum evolutionarily safe mutation rate under treatment considering growth phase only.** The formulas represented by the purple and blue line correspond to Eqs. 27 and Eq. 33 in the Methods. We observe perfect agreement. Parameters: $u_{0}={10}^{-6}$ per bp, $u_{1}=3\cdot{10}^{-6}$ per bp, $b=7.61$ per day, $a=3$ per day, $T=5$ days and $m$ as shown. The code used to generate this figure can be found at DOI: 10.5281/zenodo.8017992.


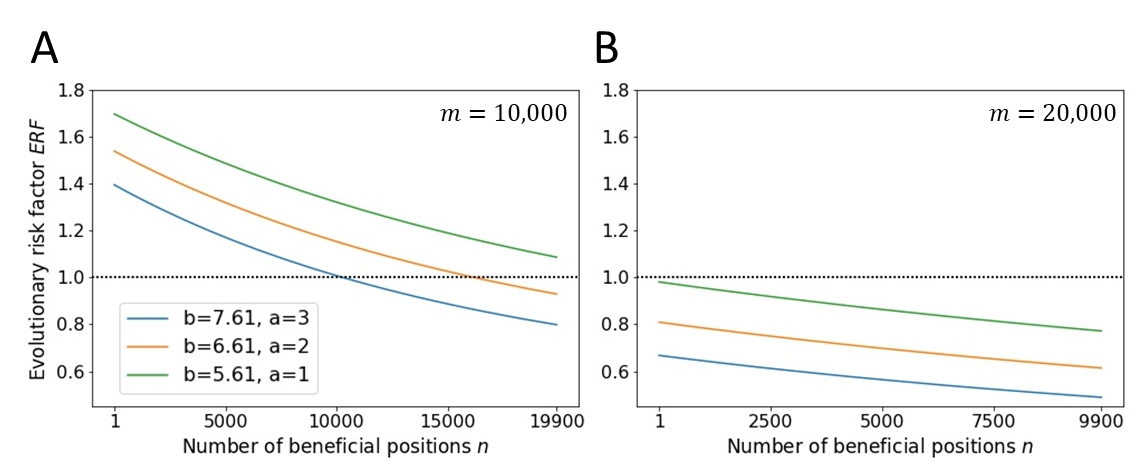


**Fig A21. The evolutionary risk factor is a declining function of the number of positions** $\boldsymbol{n}$ **also in the simplified setting.** ERF (as given by Eq. 31 in Methods) is a declining function of $n$. Parameters: $u_{0}={10}^{-6}$ per bp, $u_{1}=3\cdot{10}^{-6}$ per bp, $b$, $m$, and $a$ as shown. The code used to generate this figure can be found at DOI: 10.5281/zenodo.8017992.

**
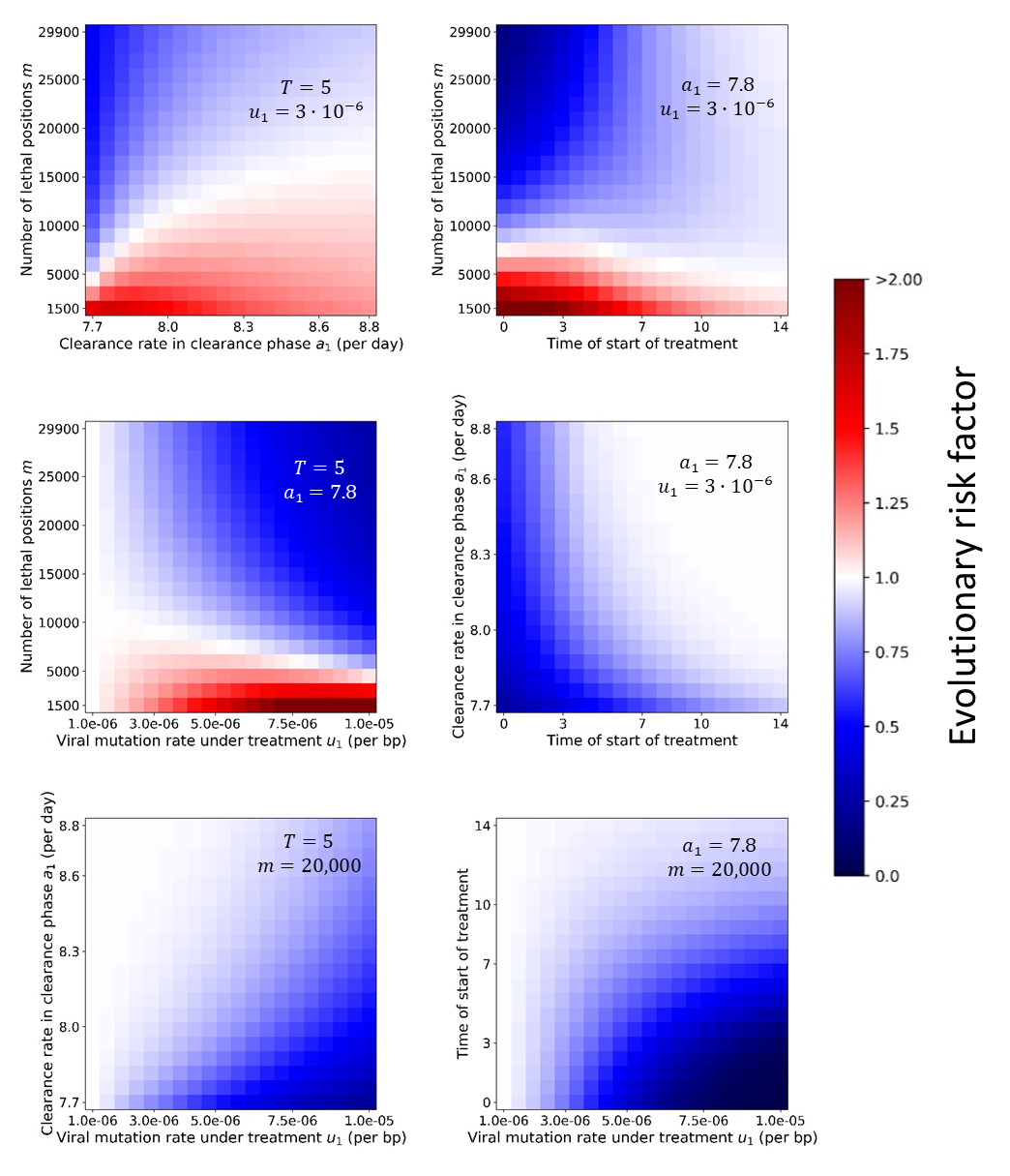
**

**Fig A22. The evolutionary risk factor (ERF) for higher-order mutants calculated with Method I.** For each pair of parameters, we numerically compute the ERF for a range of values, while all other parameters are fixed. In order to obtain the ERF, we compute the solution to Eq. 5 with the Euler method and from there, the cumulative sum of mutants $y_{01}$, $y_{10}$ and $y_{11}$ over the course of an infection with and without treatment. The ERF is then calculated as the ratio of the sum of all mutants produced with treatment to the sum of all mutants produced without treatment. We observe no difference with Figure 4, which was computed for single mutants only. Hence, under this definition of the ERF, one can establish the evolutionary safety of a treatment by considering single mutants only. Parameters: ($s_{01}, s_{10}, s_{11}$) = ($0,0,0$), $a_{0}=3$ per day and $u_{0}={10}^{-6}$ per bp. Initial condition: $y_{00}=1$, $y_{01}=0, y_{10}=0,$and $y_{11}=0$. The code used to generate this figure can be found at DOI: 10.5281/zenodo.8017992.


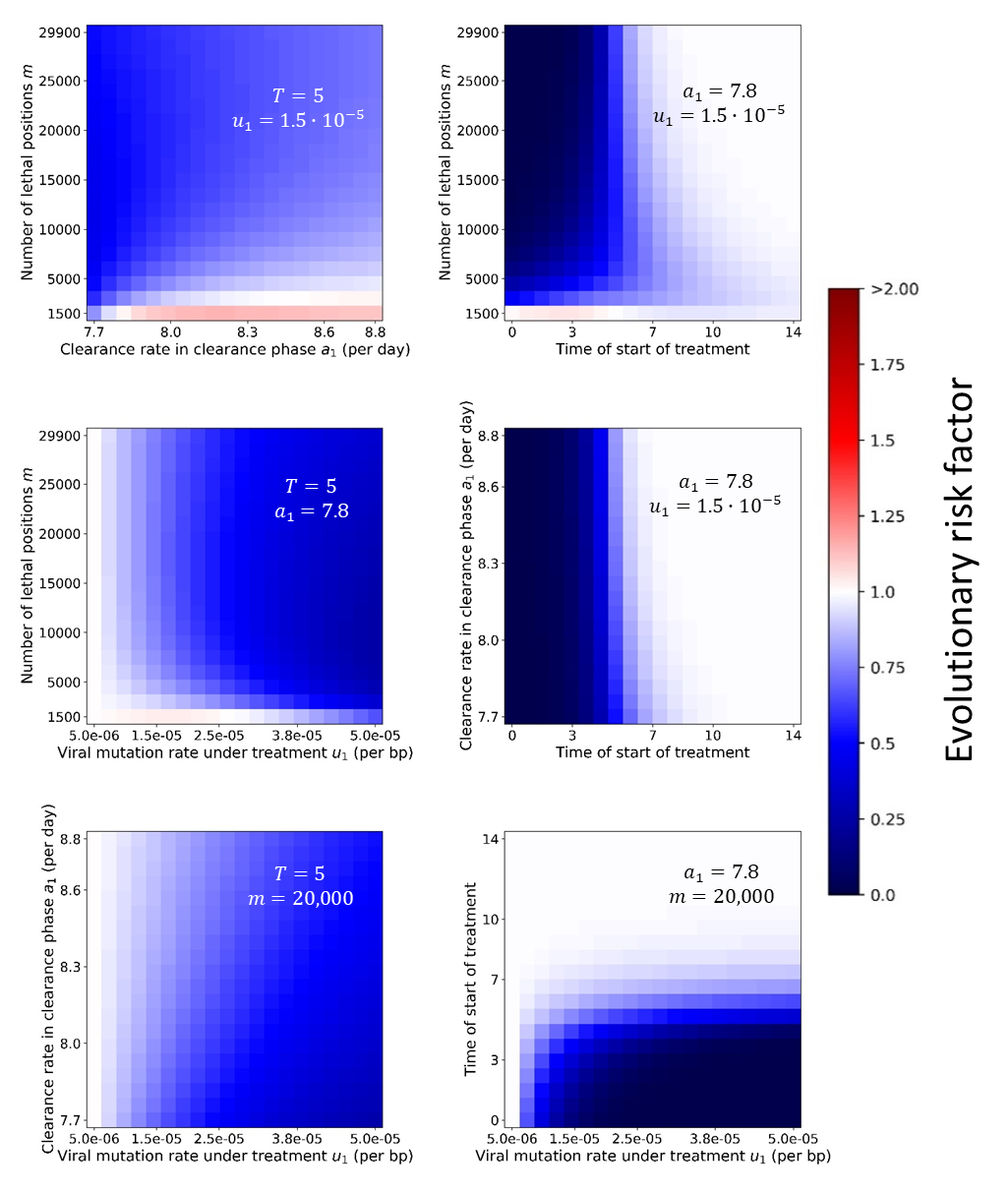


**Fig A23. The evolutionary risk factor (ERF) for higher-order mutants, when defining the ERF as the sum of all cumulative mutant abundances produced with treatment to the sum of all cumulative mutant abundances produced without treatment.** Same as Supplementary Figure 20, but $u_{0}=5\cdot{10}^{-6}$. The code used to generate this figure can be found at DOI: 10.5281/zenodo.8017992.

**
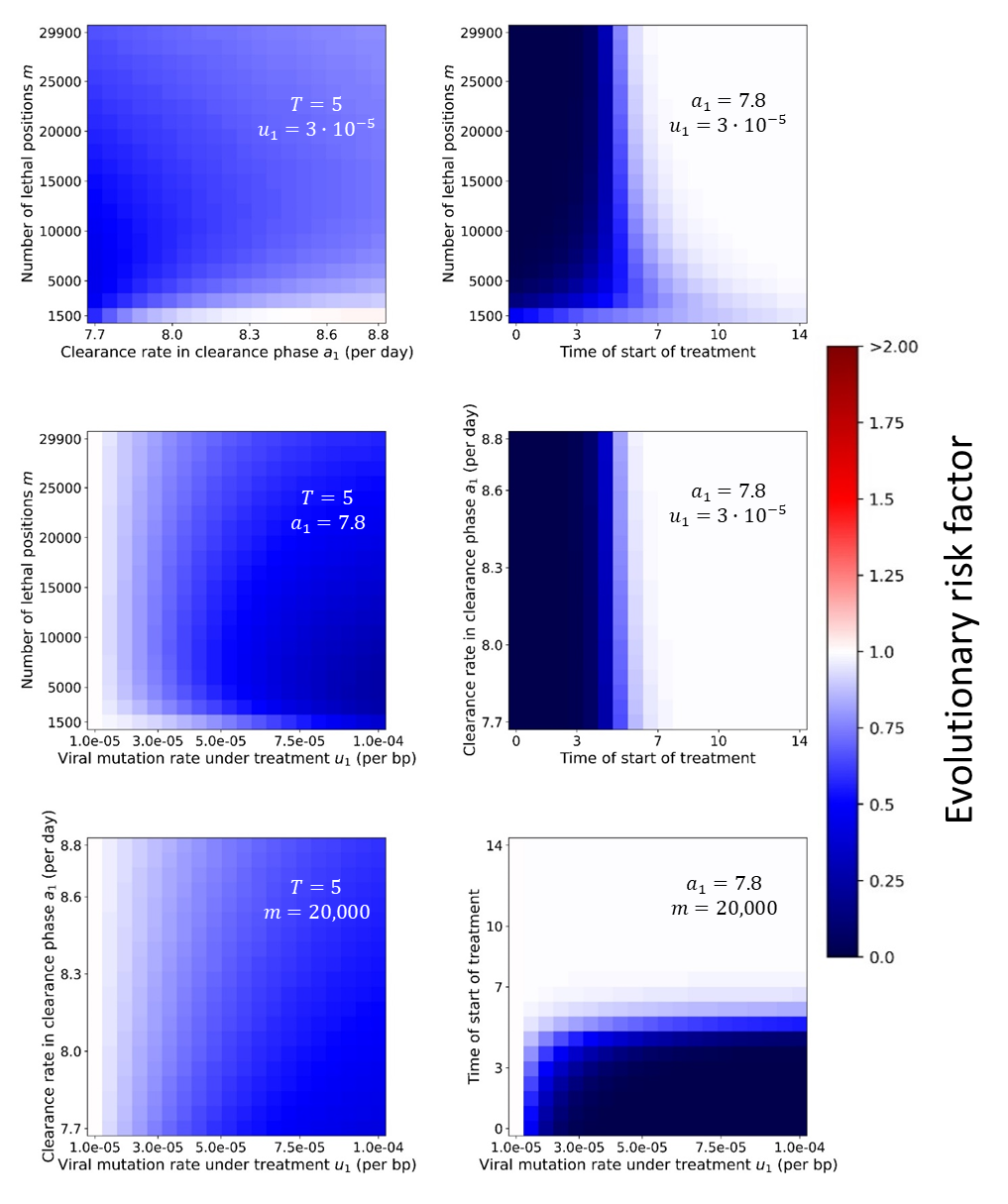
**

**Fig A24. The evolutionary risk factor (ERF) for higher-order mutants, when defining the ERF as the sum of all cumulative mutant abundances produced with treatment to the sum of all cumulative mutant abundances produced without treatment.** Same as Supplementary Figure 20, but $u_{0}={10}^{-5}$. The code used to generate this figure can be found at DOI: 10.5281/zenodo.8017992.


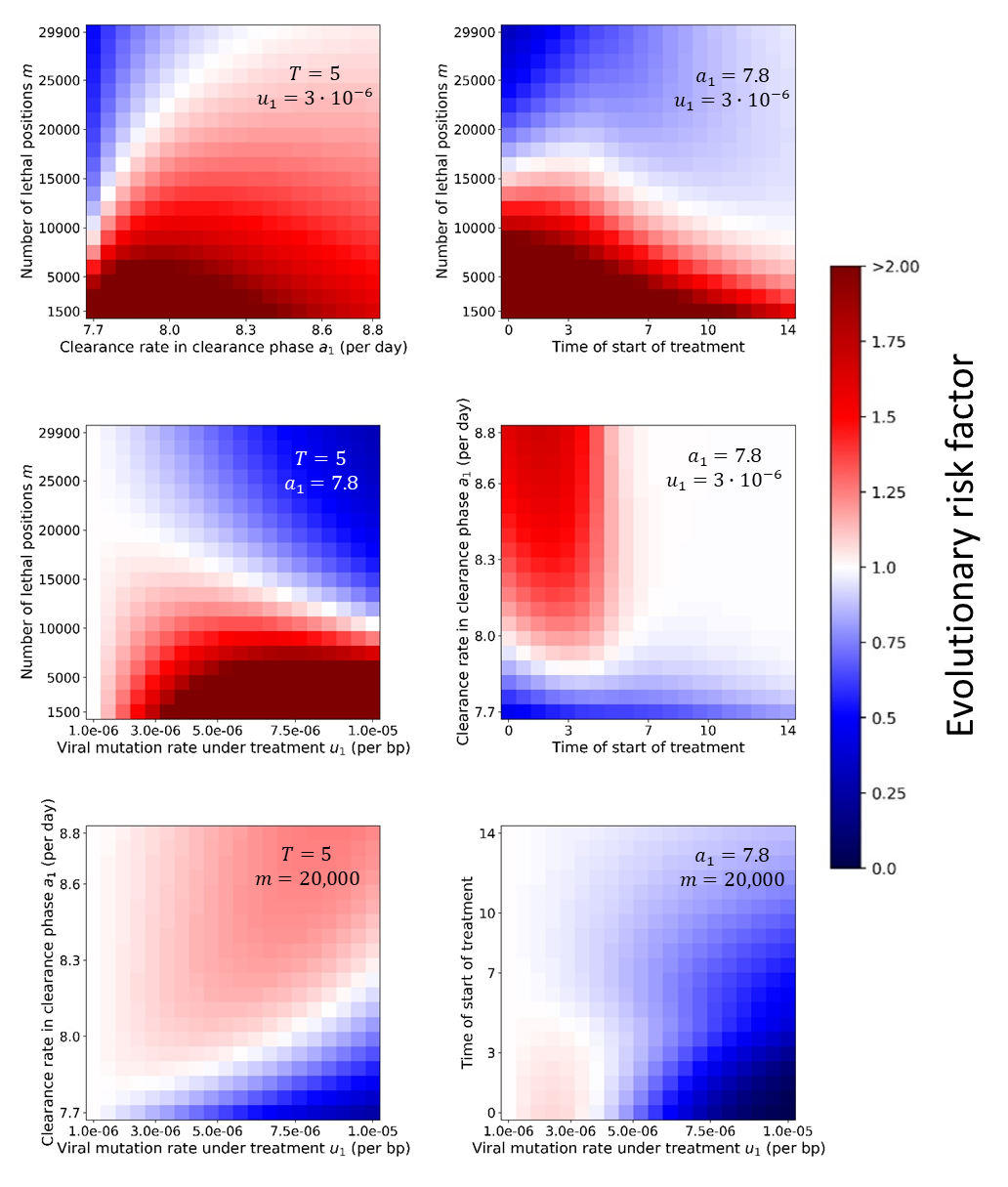


**Fig A25. The evolutionary risk factor (ERF) for higher-order mutant calculated with Method II.** For each pair of parameters, we numerically compute the ERF for a range of values, while all other parameters are fixed. In order to obtain the ERF, we compute the solution to Eq. 5 with the Euler method and from there, the cumulative sum of $y_{11}$ over the course of an infection with and without treatment. The ERF is then calculated as the ratio of the cumulative sum of $y_{11}$ produced with treatment to the sum of all mutants produced without treatment. The ERF for single mutants is plotted in Figure 3 and Supplementary Figure 5. In some cases, evolutionary safety requires a larger increase in mutation rate in order to reduce the amount of double mutant. Parameters: ($s_{01}, s_{10}, s_{11}$) = ($0,0,0$), $a_{0}=3$ per day and $u_{0}={10}^{-6}$. Initial condition: $x_{0}=1$ and $y_{0}=0$. The code used to generate this figure can be found at DOI: 10.5281/zenodo.8017992.

**
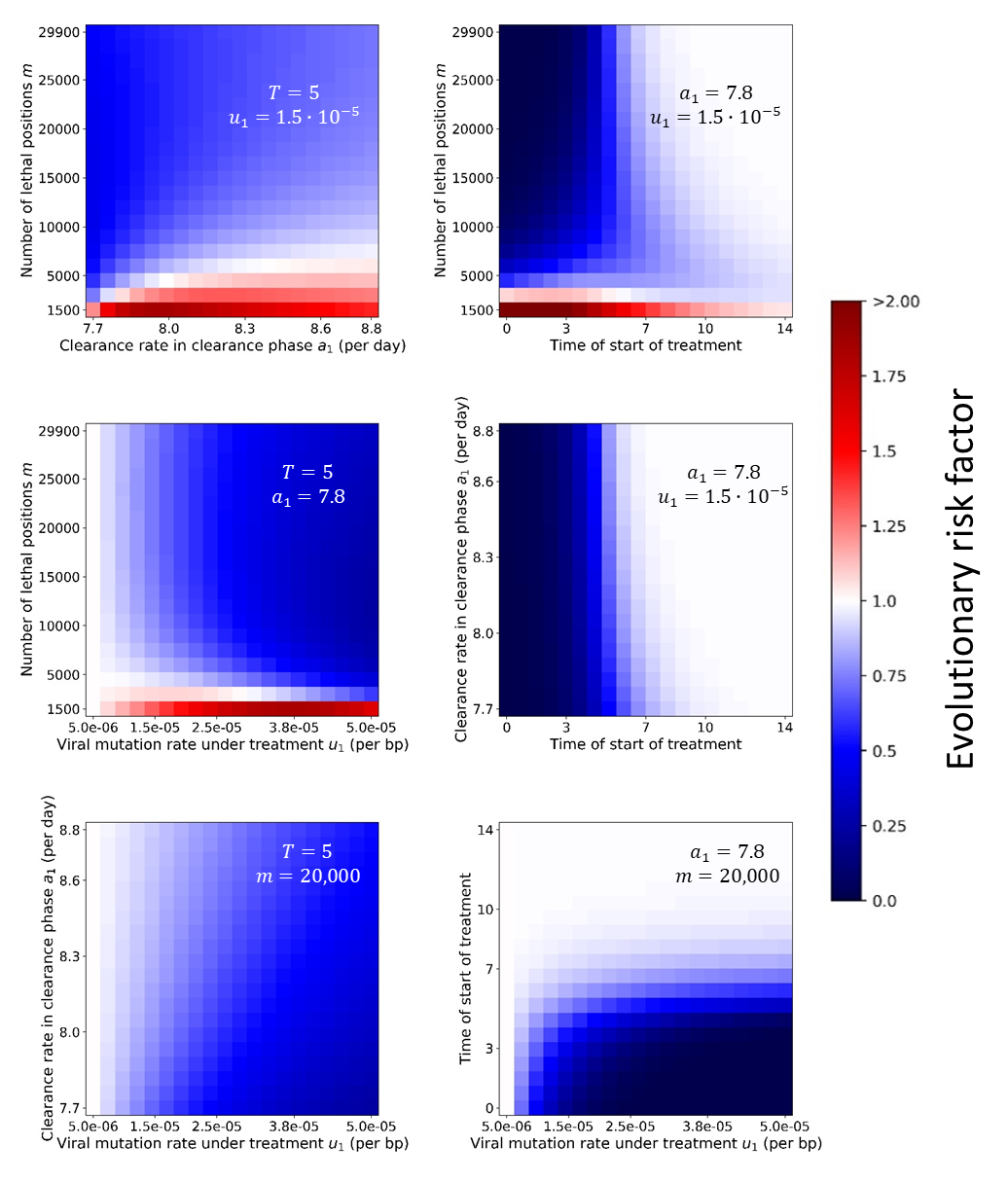
**

**Fig A26. The evolutionary risk factor (ERF) for the double mutant only.** Same as Supplementary Figure 23, except $u_{0}=5\cdot{10}^{-6}$. The code used to generate this figure can be found at DOI: 10.5281/zenodo.8017992.


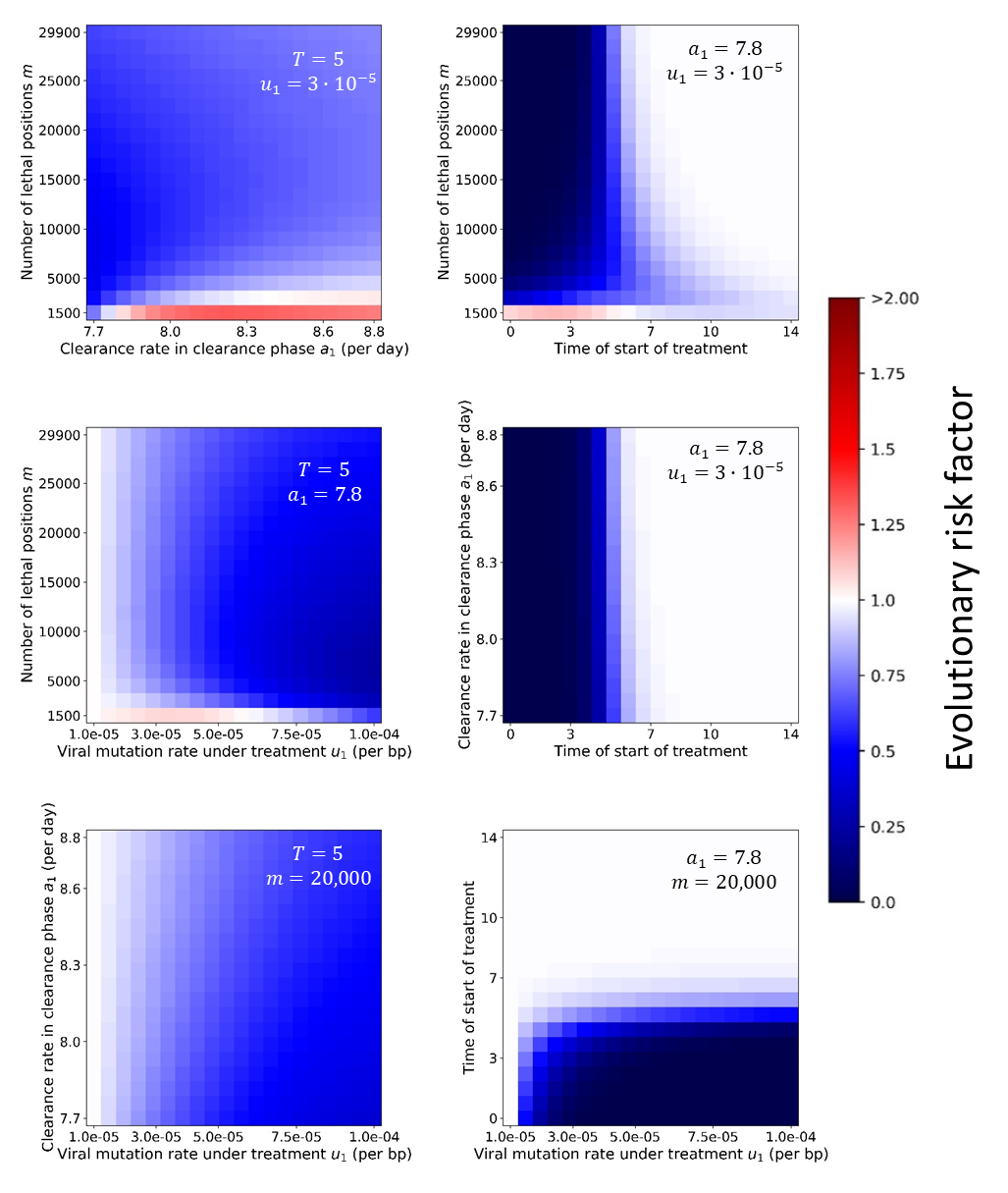


**Fig A27. The evolutionary risk factor (ERF) for the double mutant only.** Same as Supplementary Figure 23, except $u_{0}={10}^{-5}$. The code used to generate this figure can be found at DOI: 10.5281/zenodo.8017992.


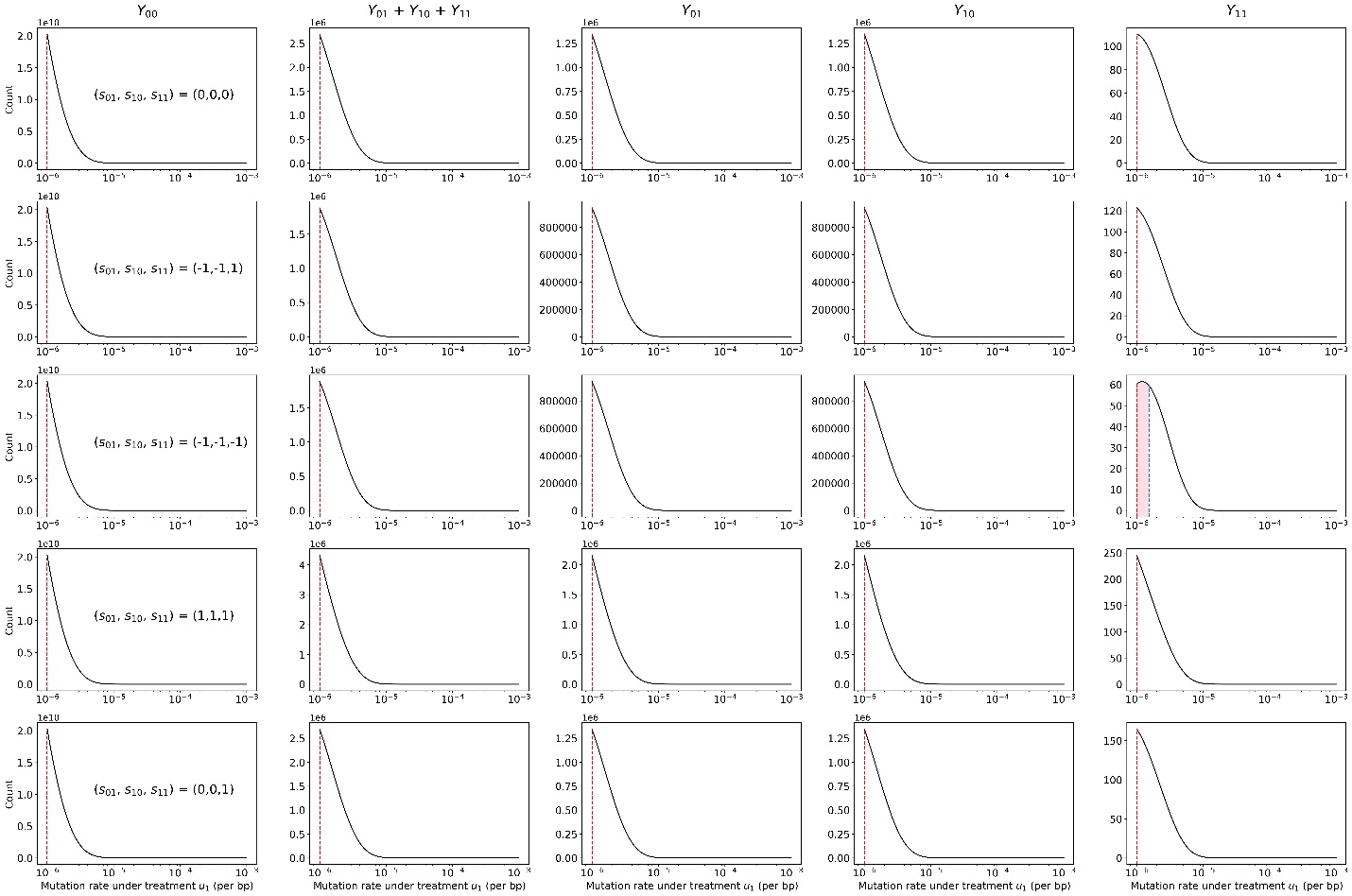


**Fig A28. Cumulative mutant virus load for wild-type, two single mutants and the corresponding double mutant.** We simulate Eq. 5 with the Euler method. We then calculate the integral of the obtained numerical solution, which corresponds to the cumulative mutant load over the course of an infection, and plot it against the virus mutation rate under treatment. The shaded area indicates mutation rates which enhance the cumulative abundance of specific virus mutants. We notice that the fitness landscape has a very moderate effect on the cumulative mutant load along mutation rate. Note that the abundance of the double mutant is roughly 1000-fold lower than that of single mutants. Treatment starts at infection. Initial condition: $y_{00}=1$; $y_{01}=y_{10}=y_{11}=0$. Parameters: $b=7.61$ per day, $a_{0}=3$ per day, $u_{0}={10}^{-6}$ per bp, $m=20,000$, $a_{1}=7.7$, $T=5$ days, $d=0.01$. The code used to generate this figure can be found at DOI: 10.5281/zenodo.8017992.


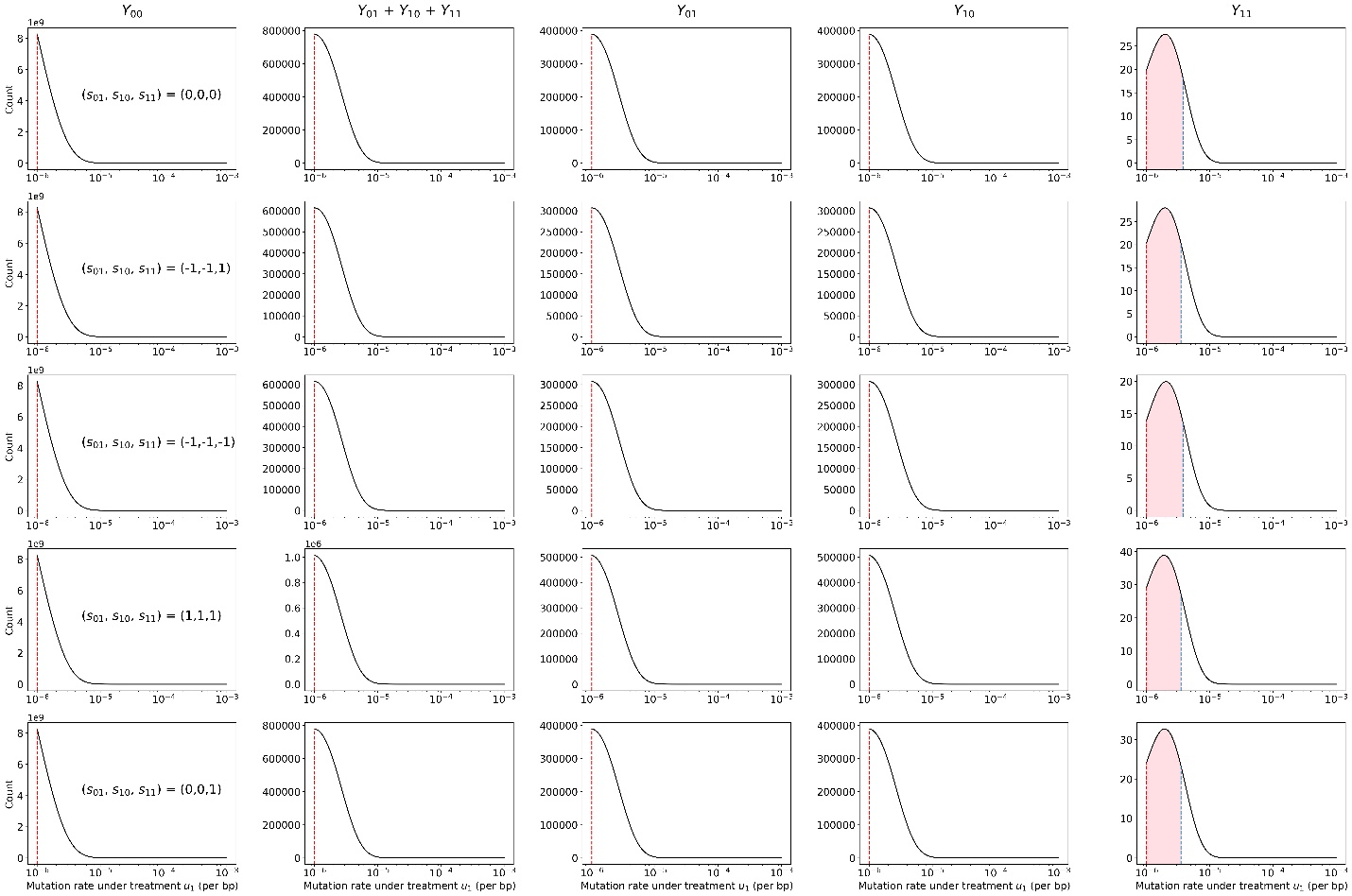


**Fig A29. Cumulative mutant virus load for wild-type, two single mutants and the corresponding double mutant.** Same as Supplementary Figure 26, but with $a_{1}=8.1$. The code used to generate this figure can be found at DOI: 10.5281/zenodo.8017992.


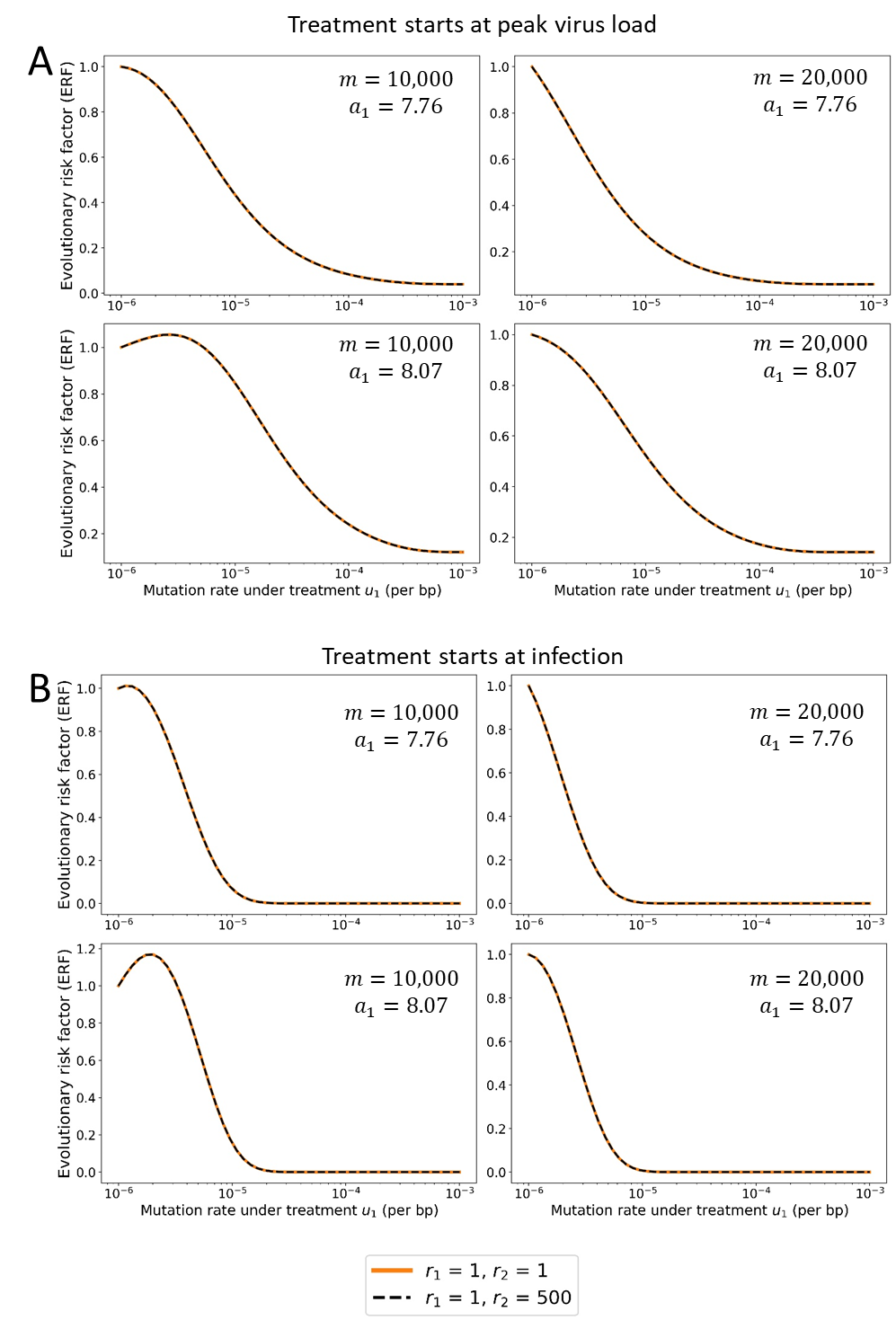


**Fig A30. Evolutionary risk factor (ERF) versus mutation rate under treatment.** Here we consider two types of mutants. The second one is considered twice as concerning as the first one. In the main text, and here for the black dashed line, the ERF is calculated as the ratio between the cumulative mutant load with treatment to the cumulative mutant load without treatment. We compute the cumulative mutant load with the Euler method applied to Eq. 1 and calculated the weighted version of ERF using Eq. 39 (when treatment begins at peak) and Eq. 40 (when treatment begins at infection). In the weighted version of the ERF, some more concerning mutants are weighted by a factor of 500 in order to give them a higher importance in the computation of the ERF. We observe that the computations give almost indistinguishable results. This is because the two mutants are differentiated by the number of positions in the genome of the virus where they are mutated. As seen in Figure 5, the number of positions that give rise to viable mutants when mutated has a very small effect on the ERF. Parameters: $b=7.61$ per day, $a_{0}=3$ per day, $T=5$ days, $u_{0}={10}^{-6}$ per bp, $n_{1}=50$ positions, $n_{2}=50$ positions. The code used to generate this figure can be found at DOI: 10.5281/zenodo.8017992.


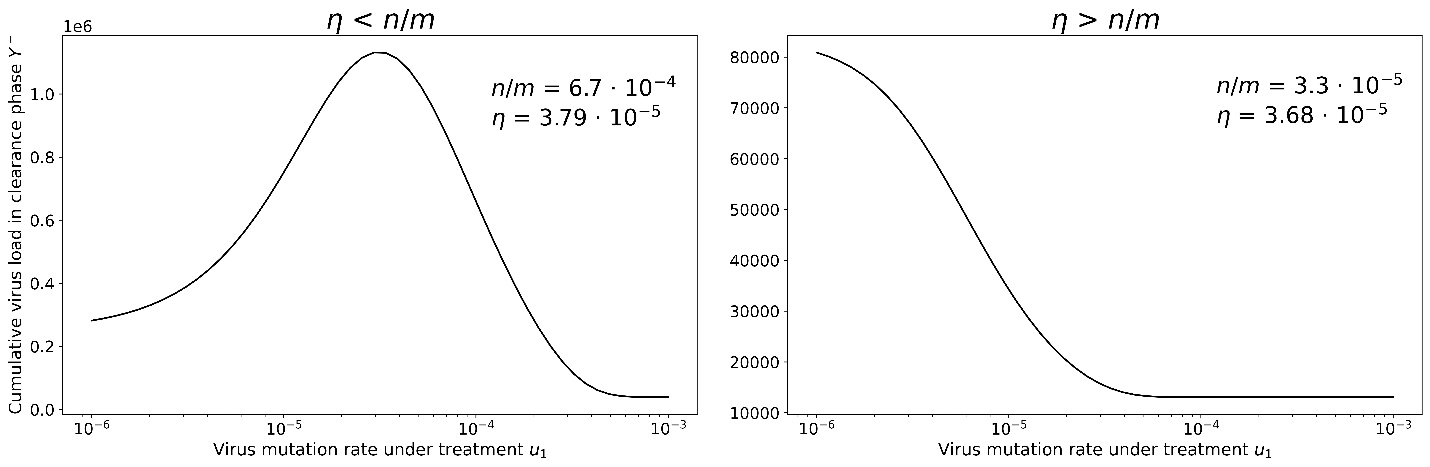


**Fig A31. Behavior of the cumulative load of the mutant virus in clearance phase along the mutation rate under treatment** $\boldsymbol{u}_{\mathbf{1}}$**.** Left: When $\eta<n/m$, the function has a single maximum. Right: When $\eta>n/m$, the function is a declining function along $u_{1}$. Parameters: $b=7.6$, $a_{0}=3$, $u_{0}={10}^{-6}$, $T=5$, $a_{1}=9$. Initial condition: $x_{0}=1$, $y_{0}=0$. The code used to generate this figure can be found at DOI: 10.5281/zenodo.8017992.

# Supplementary Tables

| Method of estimation | Parameter | | | Reference |
| --- | --- | --- | --- | --- |
|  | Peak of virus load | Time until reaching the peak of virus load | Time from peak of virus load until clearance |  |
| SARS-CoV2 challenge in young adults (pre-Alpha strain) | ${10}^{9}$ RNA copies/mL | 5 days | 7-14 days | Killingley et al. [6] |
| Virus load measurement in hospitalized patients + modelling | ${10}^{10}$ RNA copies/mL | 5 days | 9 days (younger)  16 days (older) | Neant et al. [7] |
| Virus load measurement in hospitalized patients + modelling | ${10}^{6}-{10}^{8}$ RNA copies/mL | ~3-8 days | ~10 days | Ke et al.  [8] |
| Virus load measurement in hospitalized patients + modelling  (Delta variant) | - | ~4-6 days | 8-14 days | Wang et al. [9] |
| Virus load measurement in patients | - | 4 days | 9-14 days | Lim et al. [10] |
| Virus load measurements + modelling | ${10}^{8}$ /swab | ~ 5 days | 10-30 days | Jones et al. [11] |

**Table A1. Summary of existing literature about estimates of the peak of the virus load, of the time to reach the peak of the virus load, and the time between the peak of virus load until clearance.** In cases where the paper only reported clearance from symptom onset, we assumed that the symptom onset was concomitant with the peak of the virus load. Data was taken from Refs [4,6–11].


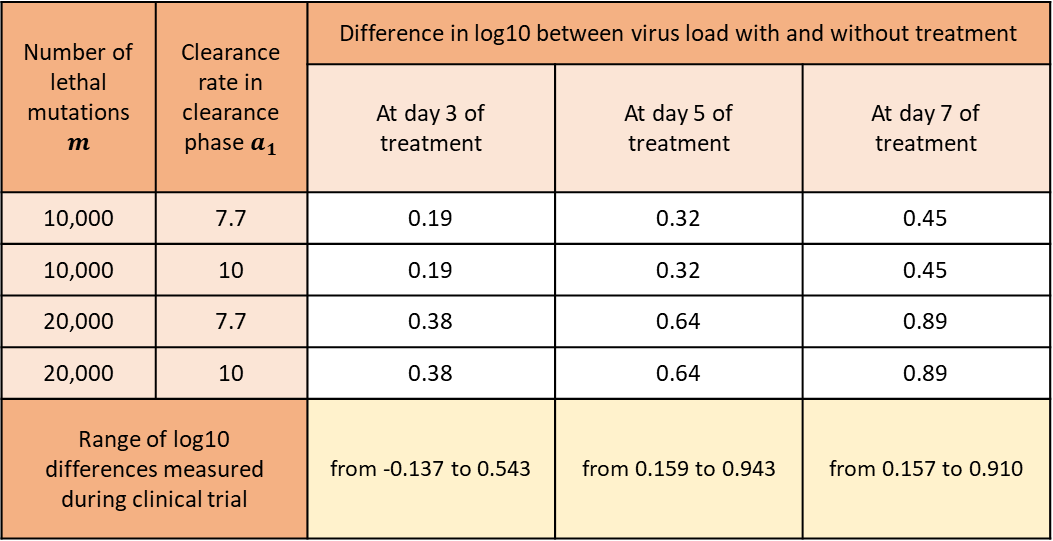


**Table A2. Difference in log10 virus load with and without Molnupiravir treatment for a range of parameters.** In the majority of cases, the log10 differences fall within the range estimated in the Molnupiravir clinical trial. Therefore, our estimates for the parameters of the model are realistic. Treatment begins at 5 days, which corresponds to the peak of the virus load. Initial condition: $x_{0}=1$ and $y_{0}=0$. Parameters: $b=7.61$ per day, $a_{0}=3$ per day, $T=5$ days.

| Fitness landscape | Peak of $Y_{01}$  or $Y_{10}$ | Peak $Y_{11}$ | Minimum fold increase for evolutionary safety $Y_{01}$ | Minimum fold increase for evolutionary safety $Y_{10}$ | Minimum fold increase for evolutionary safety $Y_{11}$ |
| --- | --- | --- | --- | --- | --- |
| ($0,0,0$) | 1,347,430 | 110 | 1 | 1 | 1 |
| ($0,0,1$) | 1,347,430 | 165 | 1 | 1 | 1 |
| ($0,0,-1$) | 1,347,430 | 82 | 1 | 1 | 1.2 |
| ($0,1,0$) | 1,347,430 | 138 | 1 | 1 | 1 |
| ($0,1,1$) | 1,347,430 | 205 | 1 | 1 | 1 |
| ($0,1,-1$) | 1,347,430 | 103 | 1 | 1 | 1 |
| ($0,-1,0$) | 1,347,430 | 96 | 1 | 1 | 1 |
| ($0,-1,1$) | 1,347,430 | 143 | 1 | 1 | 1 |
| ($0,-1,-1$) | 1,347,430 | 71 | 1 | 1 | 1.3 |
| ($1,1,0$) | 2,161,799 | 165 | 1 | 1 | 1 |
| ($1,1,1$) | 2,161,799 | 245 | 1 | 1 | 1 |
| ($1,1,-1$) | 2,161,799 | 124 | 1 | 1 | 1 |
| ($1,-1,0$) | 941,700 | 123 | 1 | 1 | 1 |
| ($1,-1,1$) | 2,161,799 | 184 | 1 | 1 | 1 |
| ($1,-1,-1$) | 941,700 | 92 | 1 | 1 | 1 |
| ($-1,-1,0$) | 941,700 | 81 | 1 | 1 | 1.2 |
| ($-1,-1,1$) | 941,700 | 122 | 1 | 1 | 1 |
| ($-1,-1,-1$) | 941,700 | 61 | 1 | 1 | 1.5 |

**Table A3. Minimum-fold increase in the virus mutation rate required for evolutionary safety for single and double mutants for the epistatic model (see Section “Evolutionary safety for higher-order mutants”).** We simulate the frequencies of the wild-type $y_{00}$ and mutants ($y_{01}$, $y_{10}$ and $y_{11}$) by applying the Euler method on Eq. 5. We then compute the integrals for each mutant ($Y_{01}$, $Y_{10}$ and $Y_{11}$) for a range of virus mutation rates under treatment. The minimum fold-increase in mutation rate that is evolutionarily safe is the minimum mutation rate under treatment for which the cumulative mutant load with treatment equal the cumulative mutant load without treatment. If the cumulative mutant load with treatment is always smaller than the cumulative mutant load without treatment, then this increase is 1. We notice that the double mutant requires a higher-fold increase in the mutation rate under treatment in order to achieve evolutionary safety. Considering mutants with a fitness advantage increases evolutionary safety but also increases the absolute mutant virus load generated over the course of an infection. Treatment starts at infection. Parameters: $b=7.61$ per day, $u_{0}={10}^{-6}$ per bp, $a_{0}=3$ per day, $m=20,000$ and $a_{1}=7.7$. Initial condition: $y_{00}=1$; $y_{01}=0$; $y_{10}=0$; and $y_{11}=0$.

| Fitness landscape | Peak of $Y_{01}$  or $Y_{10}$ | Peak $Y_{11}$ | Minimum fold increase for evolutionary safety $Y_{01}$ | Minimum fold increase for evolutionary safety $Y_{10}$ | Minimum fold increase for evolutionary safety $Y_{11}$ |
| --- | --- | --- | --- | --- | --- |
| ($0,0,0$) | 389,393 | 27 | 1 | 1 | 3.8 |
| ($0,0,1$) | 389,393 | 32 | 1 | 1 | 3.5 |
| ($0,0,-1$) | 389,393 | 23 | 1 | 1 | 3.8 |
| ($0,1,0$) | 389,393 | 30 | 1 | 1 | 3.5 |
| ($0,1,1$) | 389,393 | 35 | 1 | 1 | 3.5 |
| ($0,1,-1$) | 389,393 | 25 | 1 | 1 | 3.8 |
| ($0,-1,0$) | 389,393 | 25 | 1 | 1 | 3.8 |
| ($0,-1,1$) | 389,393 | 30 | 1 | 1 | 3.5 |
| ($0,-1,-1$) | 389,393 | 21 | 1 | 1 | 3.8 |
| ($1,1,0$) | 507,340 | 32 | 1 | 1 | 3.5 |
| ($1,1,1$) | 507,340 | 38 | 1 | 1 | 3.5 |
| ($1,1,-1$) | 507,340 | 28 | 1 | 1 | 3.5 |
| ($1,-1,0$) | 307,195 | 28 | 1 | 1 | 3.5 |
| ($1,-1,1$) | 507,340 | 33 | 1 | 1 | 3.5 |
| ($1,-1,-1$) | 307,195 | 24 | 1 | 1 | 3.8 |
| ($-1,-1,0$) | 307,195 | 23 | 1 | 1 | 3.8 |
| ($-1,-1,1$) | 307,195 | 27 | 1 | 1 | 3.5 |
| ($-1,-1,-1$) | 307,195 | 19 | 1 | 1 | 3.8 |

**Table A4. Minimum-fold increase in the virus mutation rate required for evolutionary safety for single and double mutants for the epistatic model (see Section “Evolutionary safety for higher-order mutants”).** Same as Supplementary Table 3, but for $a_{1}=8.1$.

**References**

1. Kim KS, Ejima K, Iwanami S, Fujita Y, Ohashi H, Koizumi Y, et al. A quantitative model used to compare within-host SARS-CoV-2, MERS-CoV, and SARS-CoV dynamics provides insights into the pathogenesis and treatment of SARS-CoV-2. PLOS Biol. 2021;19: e3001128. doi:10.1371/journal.pbio.3001128

2. Young BE, Ong SWX, Kalimuddin S, Low JG, Tan SY, Loh J, et al. Epidemiologic Features and Clinical Course of Patients Infected With SARS-CoV-2 in Singapore. JAMA. 2020;323: 1488–1494. doi:10.1001/jama.2020.3204

3. Zou L, Ruan F, Huang M, Liang L, Huang H, Hong Z, et al. SARS-CoV-2 Viral Load in Upper Respiratory Specimens of Infected Patients. N Engl J Med. 2020;382: 1177–1179. doi:10.1056/NEJMc2001737

4. Kim ES, Chin BS, Kang CK, Kim NJ, Kang YM, Choi JP, et al. Clinical Course and Outcomes of Patients with Severe Acute Respiratory Syndrome Coronavirus 2 Infection: a Preliminary Report of the First 28 Patients from the Korean Cohort Study on COVID-19. J Korean Med Sci. 2020;35: e142–e142. doi:10.3346/jkms.2020.35.e142

5. Wölfel R, Corman VM, Guggemos W, Seilmaier M, Zange S, Müller MA, et al. Virological assessment of hospitalized patients with COVID-2019. Nature. 2020;581: 465–469. doi:10.1038/s41586-020-2196-x

6. Killingley B, Mann AJ, Kalinova M, Boyers A, Goonawardane N, Zhou J, et al. Safety, tolerability and viral kinetics during SARS-CoV-2 human challenge in young adults. Nat Med. 2022;28: 1031–1041. doi:10.1038/s41591-022-01780-9

7. Néant N, Lingas G, Le Hingrat Q, Ghosn J, Engelmann I, Lepiller Q, et al. Modeling SARS-CoV-2 viral kinetics and association with mortality in hospitalized patients from the French COVID cohort. Proc Natl Acad Sci. 2021;118: e2017962118. doi:10.1073/pnas.2017962118

8. Ke R, Zitzmann C, Ho DD, Ribeiro RM, Perelson AS. In vivo kinetics of SARS-CoV-2 infection and its relationship with a person’s infectiousness. Proc Natl Acad Sci. 2021;118: e2111477118. doi:10.1073/PNAS.2111477118/-/DCSUPPLEMENTAL

9. Wang Y, Chen R, Hu F, Lan Y, Yang Z, Zhan C, et al. Transmission, viral kinetics and clinical characteristics of the emergent SARS-CoV-2 Delta VOC in Guangzhou, China. eClinicalMedicine. 2021;40. doi:10.1016/J.ECLINM.2021.101129/ATTACHMENT/75433689-84ED-4F43-90B7-C8516E80D9E2/MMC3.PDF

10. Lim AY, Cheong HK, Oh YJ, Lee JK, So JB, Kim HJ, et al. Modeling the early temporal dynamics of viral load in respiratory tract specimens of COVID-19 patients in Incheon, the Republic of Korea. Int J Infect Dis. 2021;108: 428. doi:10.1016/J.IJID.2021.05.062

11. Jones TC, Guido B, Barbara M, Talitha V, Julia S, Jörn B-S, et al. Estimating infectiousness throughout SARS-CoV-2 infection course. Science (80- ). 2021;373: eabi5273. doi:10.1126/science.abi5273
