# Supplementary material for: Evolutionary safety of lethal mutagenesis driven by antiviral treatment
Source: PLoS Biol. 2023 Aug 8;21(8):e3002214. doi: 10.1371/journal.pbio.3002214 (PMC10409280; doi:10.1371/journal.pbio.3002214)
Supplement: S2 Text — (PDF) [file pbio.3002214.s002.pdf]

Evolutionary safety of lethal mutagenesis –S2 Text File: Step-by-step derivation of Eq. 22

Gabriela Lobinska[1], Yitzhak Pilpel[1]*, Martin A Nowak[2]*

1 Department of Molecular Genetics, Weizmann Institute of Science, Rehovot 76100, Israel

2 Department of Mathematics, Department of Organismic and Evolutionary Biology, Harvard University, Cambridge MA 02138, USA

*corresponding authors

$$V = \frac{(a_1 - a_0)e^{(bq^m - a_0)T}}{(bq^m - a_0)(a_1 - bq^m)}$$

$$X = \frac{(a_1 - a_0)e^{(bq^{m+n} - a_0)T}}{(bq^{m+n} - a_0)(a_1 - bq^{m+n})}$$

$$Y = V - X$$

Let us introduce the function:

$$F(x) = \frac{e^{bTq^x}}{(q^x - g_0)(g_1 - q^x)} \tag{1}$$

Here $g_i = a_i/b$. Notice that $g_0 < 1 < g_1$. We have

$$V = F(m)\,(g_1 - g_0)\,e^{-a_0T}$$

$$X = F(m+n)\,(g_1 - g_0)\,e^{-a_0T}$$

$$Y = [F(m) - F(m+n)](g_1 - g_0)\,e^{-a_0T}$$

Approximating $q^x = (1-u)^x \approx 1 - ux$ we get

$$F(x) = \frac{e^{bT(1-ux)}}{(1 - ux - g_0)(g_1 - 1 + ux)} \tag{2}$$

Let $f_0 = 1 - g_0 > 0$ and $f_1 = g_1 - 1 > 0$. We get

$$F(x) = \frac{e^{bT(1-ux)}}{(f_0 - ux)(f_1 + ux)} \tag{3}$$

For $ux \ll 1$ we get

$$F(x) = \frac{e^{-bTux}e^{bT}}{f_0f_1 + ux(f_0 - f_1)} \tag{4}$$

Let $h = bT$ and $k = (f_0 - f_1)/(f_0f_1)$.

$$F(x) = \frac{e^{-hux}}{1 + kux}\frac{e^h}{f_0f_1} \tag{5}$$

The derivative of $F(m) - F(m+1)$ with respect to u is $[G(m+1) - G(m)]e^{h}/(f_0 f_1)$ where

$$G(x) = \frac{x(h + k + hkxu)e^{-hxu}}{(1 + kxu)^2}$$

The optimum of u is found by solving $G(m) = G(m+1)$ for u. We have

$$\frac{m(h + k + hkmu)e^{-hmu}}{(1 + kmu)^2} = \frac{(m+1)(h + k + hk(m+1)u)e^{-h(m+1)u}}{(1 + k(m+1)u)^2}$$

Canceling e^{-hmu} we get

$$\frac{m(h + k + hkmu)}{(1 + kmu)^2} = \frac{(m+1)(h + k + hk(m+1)u)e^{-hu}}{(1 + k(m+1)u)^2}$$

Approximating $e^{-hu} \approx 1 - hu$ we get

$$\frac{m(h + k + hkmu)}{(1 + kmu)^2} = \frac{(m+1)(h + k + hk(m+1)u)(1 - hu)}{(1 + k(m+1)u)^2}$$

Rearranging we get

$$\left(\frac{1 + kmu + ku}{1 + kmu}\right)^2 = \left(1 + \frac{1}{m}\right)(1 - hu)\frac{h + k + hkmu + hku}{h + k + hkmu}$$

Which is

$$\left(1 + \frac{ku}{1 + kmu}\right)^2 = \left(1 + \frac{1}{m}\right)(1 - hu)\frac{h + k + hkmu + hku}{h + k + hkmu}$$

Assuming $\left(1 + \frac{ku}{1+kmu}\right)^2 \approx 1 + \frac{2ku}{1+kmu}$ we get

$$1 + \frac{2ku}{1 + kmu} = \left(1 + \frac{1}{m}\right)(1 - hu)(1 + \frac{hku}{h + k + hkmu})$$

Multiplying on the RHS and neglecting higher terms we get

$$\frac{2ku}{1 + kmu} = \frac{1}{m} - hu + \frac{hku}{h + k + hkmu}$$

Multiplying with m we get

$$\frac{2kmu}{1 + kmu} = 1 - hmu + \frac{hkmu}{h + k + hkmu}$$

Rearranging we get

$$hmu + \frac{2kmu}{1 + kmu} - \frac{hkmu}{h + k + hkmu} = 1$$

Let $x = mu$

$$hx + \frac{2kx}{1 + kx} - \frac{hkx}{h + k + hkx} = 1$$

Which is equivalent to

$$h + k + x(h^2 + k^2) + x^2 hk(2h + k) + x^3 h^2 k^2 = 0$$
