# Supplementary material for: Evolutionary safety of lethal mutagenesis driven by antiviral treatment
Source: PLoS Biol. 2023 Aug 8;21(8):e3002214. doi: 10.1371/journal.pbio.3002214 (PMC10409280; doi:10.1371/journal.pbio.3002214)
Supplement: S3 Text — (DOCX) [file pbio.3002214.s003.docx]

**Evolutionary safety of lethal mutagenesis –S3 Text File: Relationship to previous literature and model extensions**

Gabriela Lobinska^1^, Yitzhak Pilpel^1*^, Martin A Nowak^2*^

1 Department of Molecular Genetics, Weizmann Institute of Science, Rehovot 76100, Israel

2 Department of Mathematics, Department of Organismic and Evolutionary Biology, Harvard University, Cambridge MA 02138, USA

*corresponding authors

**Table of Contents**

[Relationship to previous literature 3](#_Toc129430745)

[Estimating the mutation rate 6](#_Toc129430746)

[Stamping vs. linear replication 6](#_Toc129430747)

[Plus-minus-plus replication cycle 8](#_Toc129430748)

[RNA editing 10](#_Toc129430749)

[Non-lethal deleterious mutations 10](#_Toc129430750)

[Lethal defection 12](#_Toc129430751)

# **Relationship to previous literature**

Killingley et al. (2022)[1] purposely infected 18 healthy volunteers with the pre-alpha variant of SARS-COV2. They then monitored symptom progression, viral kinetics and serological conversion until viral clearance. Their study enables the tracking of the viral kinetics of SARS-COV2 from the moment of infection, which is otherwise impossible. Their estimates for the peak of the virus load, the virus load at peak infection and the time to first negative PCR are in agreement with the parameters that we used in our model.

Kim et al. (2021)[2] sought to understand the differences in the viral of kinetics of SARS-COV2 in asymptomatic, symptomatic and non-surviving patients. They measured the virus load of patients at different times of the infection course. They found the time to viral clearance is significantly different between symptomatic and asymptomatic patients and varies between 18-33 days after symptom onset, which corresponds to our lower estimates for the rate of viral clearance during the clearance phase.

Neant et al. (2021)[3] analyzed viral dynamics of 655 hospitalized patients. Using a target cell-limited model with three compartments, they are able to reproduce the observed viral dynamics and distill features predictive of the risk of complications and death. Neant et al. found that the time to viral clearance is about 13-16 days after symptom onset, but with considerable variability between patients. These estimates are in line with our estimate for the rate of clearance in the clearance phase.

Ke et al. (2021)[4] link the virus load of SARS-COV2 with infectiousness. They use two infection datasets containing estimated times of transmission and virus load along time. The dynamics are then fitted to several models in order to find the one that best describes the data. The link with infectiousness is the established through a modelling approach including the viral load of each patients, the proportion of viable virus in each swab, as well as the estimated number of contacts of each individual. The times to viral clearance considered in this paper are in agreement with our estimates for the rate of clearance during the clearance phase.

Wang et al. (2021)[5] is among the first works characterizing the Delta subvariant of SARS-COV2, appeared in spring 2021. Wang et al. estimate the incubation time, the virus load at peak of infection and the time to viral clearance for patients infected with the Delta subvariant. They also analyze the clinical data of these patients to find that the risk of progression to severe disease as well as the transmissibility of the virus is markedly higher for the Delta subvariant.

Kim et al. (2021)[6] provide an analysis of SARS-COV2 temporal dynamics in 138 patients, measured from symptom onset until viral clearance. They provide estimates for the peak of the virus load and the time to viral clearance. Due to the extensive SARS-COV-2 surveillance program in South Korea, Kim et al. were able to obtain data from very early on in the disease course.

He et al. (2020)[7] estimate the infectiousness profile of individuals infected with SARS-COV2 based on temporal data of virus dynamics along infection as well as infector-infected pairs. In particular, they are able to infer the serial interval between infections and the time to symptom onset. Based on their data of Ct values of PCR tests performed on patients along time, He et al. estimate the time to virus clearance to be about 21 days after symptom onset. This is in line with our estimates for the virus clearance rate in the clearance phase.

Jones et al. (2021)[8] provide an analysis of virological data from over 25,000 patients. Their huge dataset includes pre-symptomatic, asymptomatic and mildly symptomatic as well as hospitalized patients. Next, they associate this data with estimate for viable virus isolation. Thus, they are able to infer the infectiousness profile of the studied individuals along time. Their estimates for the time to peak of the virus load, the virus load at peak, and the time to clearance of the virus are all in line with the estimates that we have used throughout the paper.

Kawasuji et al. (2020)[9] provide viral trends for 28 patients infected with SARS-COV2, both symptomatic and asymptomatic. They found that patients who transmitted the infection to at least one other individual had a significantly higher virus load than individuals that did not cause any secondary infections. Based on their measurement of the virus load along time, the time to virus clearance is about 10-30 days after symptom onset, which is in line with our values for the rate of viral clearance during the clearance phase.

Patel et al. (2021)[10] simulate within-person virus dynamics with a four compartment ODE model describing the evolution over time of target cells, infectious cells, non-productive infectious cells and free virus. They estimate several important features of the virus, including the reproductive ratio of SARS-COV2 within the body. They then proceed to simulate viral treatment and make several predictions on viral treatment efficiency based on their mechanism of action (e.g. stimulation of elimination of infected cells).

Rodriguez and Dobrovolny (2021)[11] fit the virus dynamics of SARS-COV2 in young and aged macaques to a standard model describing virus kinetics over time. They then compare the best-fit parameter distributions for virus kinetics in young and old macaques. Finally, they use the model to simulate the effect of antiviral treatment on the virus load.

Ejima et al. (2021)[12] set out to estimate the incubation time of SARS-COV2. To this aim, they collected virus dynamics from several sources and fitted a simple model describing virus kinetics within the patient. They estimate that between infection and the virus peak, the virus load grows by about 10 order of magnitude, which is consistent with the values that we used in our analysis. They also calculate the time between infection and the peak of the virus load to be about 5 days, which is also in agreement with the estimates that we used in our analysis.

Kern et al. (2021)[13] construct a model describing the within-patient virus kinetics and fit it to existent data of temporal virus dynamics from patients. Next, they model the pharmacokinetics and the effects of several treatments. There are able to reproduce the empirically observed efficacy of Ivermectin and the lack of efficacy of several other drugs. They suggest that modelling pharmacokinetics could be a successful method for predicting the efficacy of putative treatment for COVID-19.

Challenger et al. (2022)[14] establish a complex mechanistic model of the progression of SARS-COV2 infection within the upper respiratory tract. They fit their model to a large combined dataset of virus kinetics collected from many sources. They then examine the statistical association between the virus load in the upper respiratory tract and disease severity, age, and sex. They find no significant association.

Shannon et al. (2020)[15] study the accuracy and speed of the SARS-COV2 RNA polymerase in the presence of Favipiravir, an adenosine analogue. They find that Favipiravir is readily incorporated into nascent RNA chains and causes C-to-U and G-to-A mutations. They conclude that Favipiravir can induce lethal mutagenesis of SARS-COV2 by substantially decreasing the accuracy of the viral polymerase. Hence, Favipiravir is another example of mutagenic antiviral treatment.

Hay et al. (2021)[16] present a method to draw epidemiological conclusions from Ct values obtained from PCR tests of recently diagnosed individuals. Since the Ct value is correlated with the time from infection, the progression of an epidemic can be inferred from the obtained distribution of Ct values. Hay et al.’s approach was able to predict correctly the progression of the epidemic in Massachusetts.

Kim et al. (2021)[17] model the within-patient virus dynamics of three related viruses: SARS-COV, SARS-COV2 and MERS-COV. They estimate the time of virus peak, the virus load at peak and at symptom onset, as well as the time to virus clearance. They then use their model to simulate the effect of several antiviral drugs based on their mechanism of action.

# **Estimating the mutation rate**

## **Stamping vs. linear replication**

We simulated the replication of one virion to obtain a 100 progeny virions assuming either stamping or linear replication over three generations within the same cell. A cartoon illustrating these two modes of replication is presented in **Fig A1**.


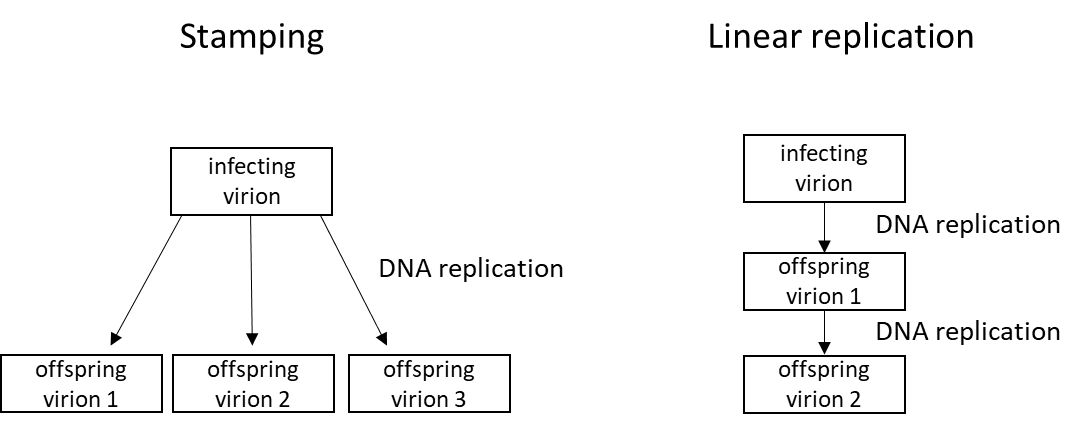


**Fig A1: Schematic representation of two modes of replication: stamping and linear replication.** During stamping, the genome of the infecting virion serves as a template for the synthesis of all offspring virions. During linear replication, the genomes of the offspring virions that have already been synthetized can serve as templates for the replication of additional viral offspring. Each mode of replication can potentially lead to a different distribution of the number of mutants arising from an infection event.

Stamping refers to replication of the 100 progeny virions using the genome of the virion that entered the cell as the template. We simulated it by randomly sampling a 100 times from a binomial distribution with parameters $n=1$ and $p=\mu$, where $\mu$ is the mutation rate.

With linear replication, the virion that entered the cell is used as a template to generate the first generation of virions, and these can be then used themselves as templates for the second generation of virions. This process can extend over up to 3 generations within a cell [18].

To simulate this mode of replication, we randomly sampled the number of mutants in the first generation from a binomial distribution with parameters $n=1$ and $p=\mu/3$, where $\mu$ is the mutation rate, similarly to what we performed for the stamping mode. We divide the mutation rate by 3 to correct for the three replication events occurring within the cell. However, instead of sampling 100 progeny virions, we sampled 4 first generation virions, a number we chose arbitrarily.

We then chose 20 as the number of virions in the second generation and performed a random sampling with replacement of the first generation mutants to obtain the parents’ of the second generation virions. We then simulated replication through the random sampling from the binomial distribution with parameters $(1,\mu/3)$. The number of mutants in the second generation was the sum of the mutations present in their parent and the mutations acquired during replication.

Lastly, we repeated this procedure to obtain the third and last generation: a 100 parent virions were chosen through random sampling with replacement of the second generation, and replicated was simulated through random sampling from a binomial distribution with parameters $(1,\mu/3)$. The number of mutations in the progeny was the sum of the number of mutations in the parent and the number of mutations generated during replication.

For each mode of replication – stamping or linear replication over three generations – we plotted the distribution of the number of mutants for 5,000,000 simulation runs, and for several mutation rates. The lowest mutation rate, $\mu={10}^{-6}$ per infection cycle, represents a situation with no mutagenic treatment. Higher mutation rates represent treatments inducing different levels of mutagenesis in the virus.

We observe that the distributions are very different between the two modes of replication (see **Fig A2**). However, the shape of the distributions looks similar between the different levels of the mutation rates for each mode of replication separately. Importantly, the average number of mutants is unaffected (see **Fig A3**).


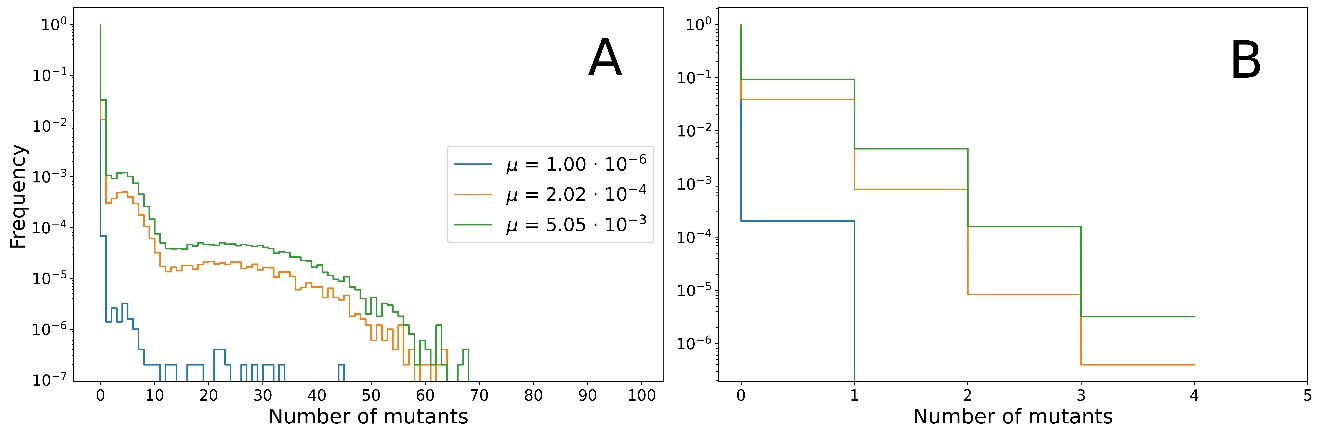


**Fig A2: Distribution of the number of mutants depending on the mode of replication: stamping (panel A) or linear replication with three generations within one cell (panel B).** Although the distribution of the number of mutants differs depending on the mode of replication, the shape of the distributions is similar for different mutations rates.


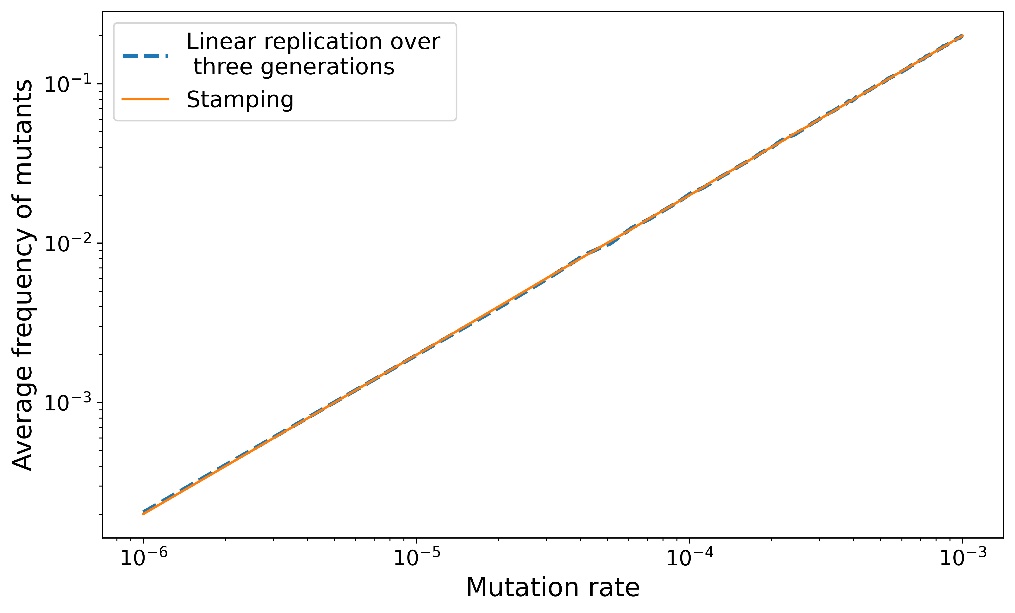


**Fig A3: Average frequency of mutants depending on the mutation rate.** It is identical for both of the considered modes of replication. Since our model is concerned with the expected number of mutations per patient, the mode of replication does not affect our conclusions.

## **Plus-minus-plus replication cycle**

SARS-CoV2 is a plus-strand RNA virus. In order to replicate, it first synthetizes an intermediate minus strand, which then serves as a template for offspring plus-strands. A cellular infection event gives rise to about 100 progeny virions [19]. Within each patient, between ${10}^{4}$ and ${10}^{6}$ cells will be infected [20].

We constructed a simulation of a cellular infection event, and compute the distribution of mutants within a patient depending on the number of intermediate minus strands, which we denote by $c_{1}$. The variable $c_{1}$ can range between 1, if all progeny virions are synthetized from the same minus template, and 100, if each progeny virion in synthetized from a different minus template. To the best of our knowledge, the value of $c_{1}$ for SARS-CoV is not known.

Our simulation runs as follows. First, we sample the number of mutations that occurred during the synthesis of the minus strand from the plus strand. We use a binomial distribution with parameters $n=L$ and $p={10}^{-6}$, and sample $c_{1}$ random variables. $L$ is the length of the genome. In practice, we do not expect more than 2 mutations per infection cycle. Hence, to reduce computation time, we used $L=2$. We thus obtain the distribution of the number of minus strands that are wild-type, single mutants or double mutants. We expect $2 \left( 1-p \right) p c_{1}$ template strands to be single mutants.

We then use this distribution to sample $c_{2}$ strands that will serve as templates to synthetize plus strands. We neglect the probability of back mutations. Hence, if a mutation occurred during the synthesis of the minus strand, it will be ensured to propagated to all plus strands synthetized from that mutant template RNA molecule. Hence, we expect $2 \left( 1-p \right) p c_{2}$ plus strands to be synthetized from a minus strand that is a single mutant.

The number of mutations occurring during the synthesis of plus strands from each parent minus strands can be obtained through another sampling of a binomial distribution, with parameters $n=L$, $p={10}^{-6}$ and size $c_{2}$. Out of $\left( 1-p \right) c_{2}$ plus strands that are synthetized from a wild-type template, $p c_{2}$ will become at least single mutants. The total number of mutations in the cell will be the sum of the number of mutations in the template strand used for the synthesis of each $c_{2}$ of plus strands and the number of mutations that occurred during the synthesis of each $c_{2}$ plus strand from the template minus strand. Hence, we expect a total of $c_{2} p+\left( 1-p \right) p c_{2}$ mutant virions in the cell. Note that this expression is independent from $c_{1}$.

We simulated this model for $c_{1}=1$, $c_{1}=25$, $c_{1}=50$ and $c_{1}=100$. In **Fig A4**, we plotted the histograms of the number of single mutants obtained from each infected individual.

Although the variance of the number of single mutants per patients varied considerably, the sum of the number of single mutants remained constant, regardless of the value of $c_{1}$.


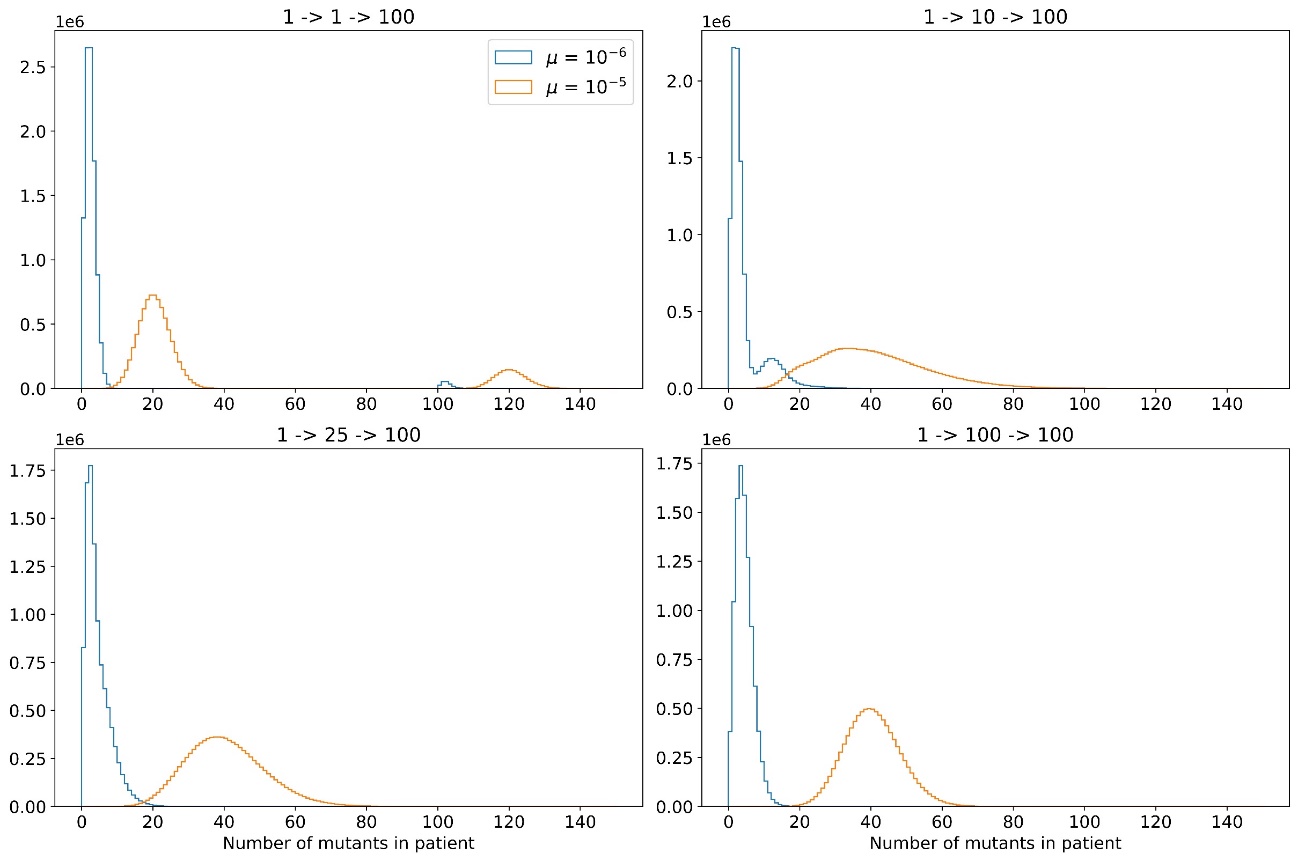


**Fig A4: Distribution of the number of mutants in the progeny genomes depending on the number of negative-strand templates.** Although the distributions are very different, they all result in the same average number of mutant in patient. Moreover, the shape of the distribution is conserved for different mutation rates, representing absence or presence of treatment.

Hence, the expected number of mutations is invariant to whether the mutations occur in the plus or in the minus strand.

This analysis of replication mode is now introduced in the main text, page 6 lines 150-155, and a supplemental file “Estimating the mutation rate” is now added to the paper with this entire analysis.

## **RNA editing**

As mentioned before, mutations stemming from RNA editing are likely to have been included in the measured mutation rate which we used. This is because these estimates were established in cell culture already capturing their RNA editing levels.

However, we acknowledge that RNA editing can be present at various levels across cell types and cell conditions [21,22].

Note that we already considered the possibility that our estimate for the mutation rate underestimated the true value of the mutation rate without treatment. In the main text, we consider the mutation rate $\mu_{0}$ without treatment to be ${10}^{-6}$ per nucleotide per cellular infection cycle. In **Figure A7** in **S1 Text**, we consider $\mu_{0}=5\cdot{10}^{-5}$. In **Figure A8** in **S1 Text**, we consider $\mu_{0}={10}^{-5}$. Following this comment, we now explicitly mention that RNA editing, with its potential variable extent across cell types, could module the apparent mutation rate of the virus.

# **Non-lethal deleterious mutations**

In addition to the abundance of wild-type, $x$, and the abundance of the potentially concerning mutant, $y_{1}$, we now also consider the abundance of deleterious mutants, $y_{2}$, and the abundance of mutants that are both deleterious and potentially concerning, $y_{3}$.

Mutation in any one of $n_{1}$ positions leads from $x$ to $y_{1}$. Mutation in any one of $n_{2}$ positions leads from $x$ to $y_{2}$. Mutation in any one of $n_{3}$ positions leads from $x$ to $y_{3}$. Mutation in any one of $n_{2}+n_{3}$ positions leads from $y_{1}$ to $y_{3}$. Mutation in any one of $n_{1}+n_{3}$ positions leads from $y_{2}$ to $y_{3}$. Back mutations are ignored. As in our original model, mutation in any one of $m$ mutations is lethal. Deleterious mutations have a birth rate $b'$ which is less than $b$. The subscript $j$ in $a_{j}$ denotes the absence ($j=0$) or presence ($j=1$) of an adaptive immune response. Let $M=m+n_{1}+n_{2}+n_{3}$. Virus dynamics are now described by

$$\dot{x}=x(bq^{M}-a_{j})$$

$$\dot{y_{1}}=xbq^{M-n_{1}}\left( 1-q^{n_{1}} \right)+y_{1}(bq^{M-n_{1}}-a_{j})$$

$$\dot{y_{2}}=xbq^{M-n_{2}}\left( 1-q^{n_{2}} \right)+y_{2}(b^{'}q^{M-n_{2}}-a_{j})$$

$$\dot{y_{3}}= xbq^{m}\left( 1-q^{n_{3}} \right)+y_{1}bq^{m}\left( 1-q^{n_{2}+n_{3}} \right)+y_{2}b^{'}q^{m}\left( 1-q^{n_{1}+n_{3}} \right)+y_{3}(b^{'}q^{m}-a_{j})$$

We computed the abundance of each category of virus over time and plotted it in **Fig A5**.


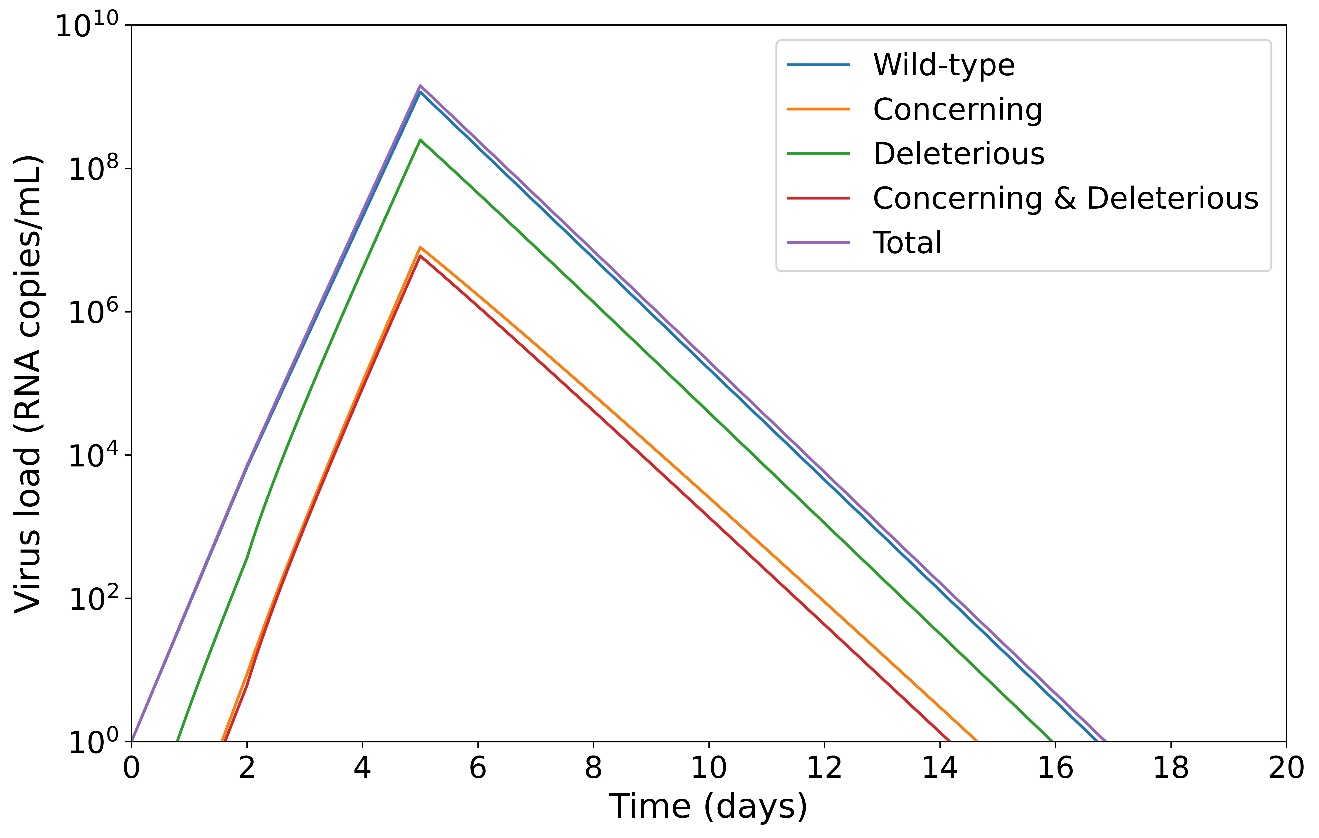


**Fig A5: Time series of total virus (**$\boldsymbol{v=x+}\boldsymbol{y}_{\boldsymbol{1}}\boldsymbol{+}\boldsymbol{y}_{\boldsymbol{2}}\boldsymbol{+}\boldsymbol{y}_{\boldsymbol{3}}$**), wild-type virus (**$\boldsymbol{x}$**), concerning virus (**$\boldsymbol{y}_{\boldsymbol{1}}$**), deleterious virus (**$\boldsymbol{y}_{\boldsymbol{2}}$**), concerning and deleterious virus (**$\boldsymbol{y}_{\boldsymbol{3}}$**).** Parameters: $b=7.61$, $b_{1}=0.9\cdot b$, $a_{0}=3$, $a_{1}=8.8$, $u_{0}={10}^{-6}$, $u_{1}=3\cdot{10}^{-6}$, $m=20,000$, $n_{1}=87$, $n_{2}=6713$, $n_{3}=100$, $T=5$. Treatment starts after 2 days. Initial condition: $x\left( 0 \right)=1$, $y_{1}\left( 0 \right)=y_{2}\left( 0 \right)=y_{3}\left( 0 \right)=0$.

The wild-type virus is always the major category. Deleterious mutants are roughly an order of magnitude less abundant than the wild-type at peak point. Concerning mutants are roughly three orders of magnitude less abundant than the wild-type. Lastly, deleterious concerning mutants are roughly two and a half orders of magnitude less abundant than the wild-type.

We then computed the ERF for a range of values of the number of lethal positions $m$ and of the clearance rate in the clearance phase $a_{1}$. The number of concerning positions is $n_{1}=87$, as previously. Additionally, we consider arbitrarily $n_{3}=100$ positions that are both deleterious and concerning. About 10% of positions in the genome are assumed that be neutral. SARS-CoV2’s genome is 29,900 nt in length, hence about 3000 positions are estimated to be neutral when mutated. Therefore, the number of positions that are deleterious when mutated is $n_{2}=29,900-87-100-m$. We plotted the results in **Fig A6**.


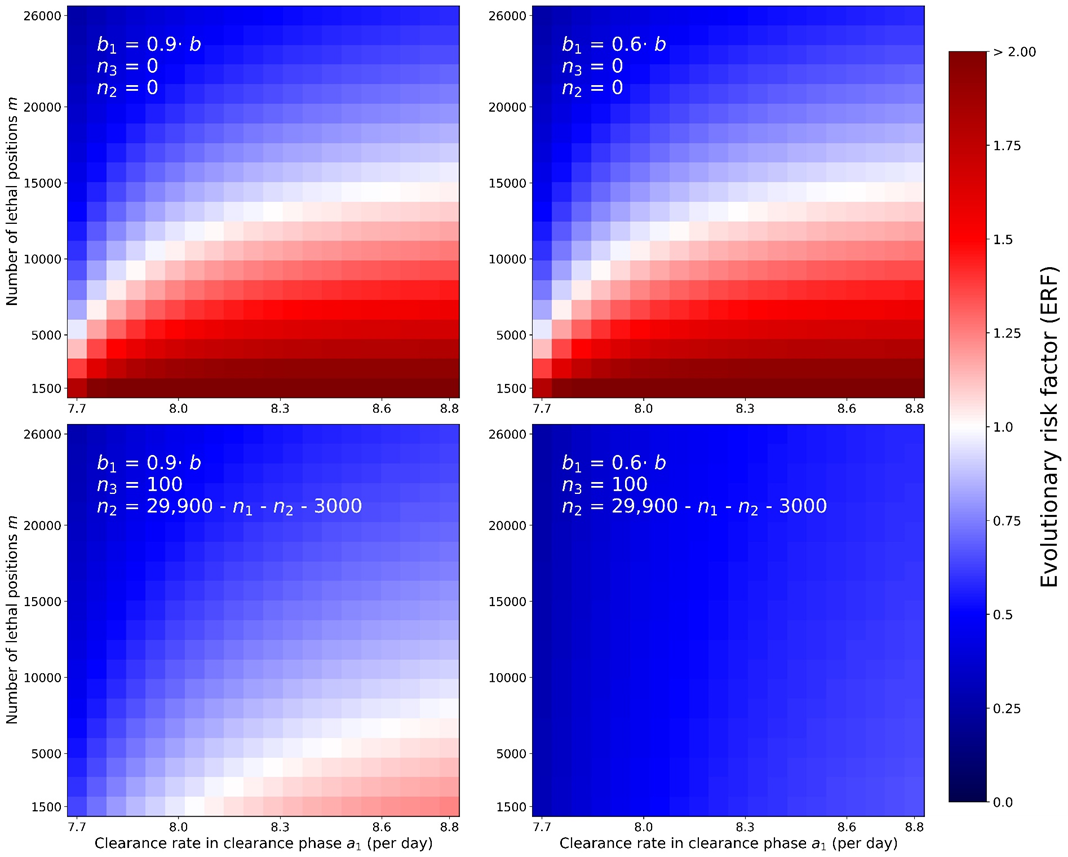


**Fig A6: Comparison of the model with and without considering non-lethal deleterious mutations.** Including non-lethal deleterious mutations increases the evolutionary safety of the treatment. The ERF is computed as the cumulative sum of the concerning mutant $y_{1}$ and the concerning and deleterious mutant $y_{3}$ with treatment, normalized by the corresponding sum without treatment. Parameters: $b=7.61$, $a_{0}=3$, $u_{0}={10}^{-6}$, $u_{1}=3\cdot{10}^{-6}$, $n_{1}=87$, $T=5$. Treatment starts after 2 days. Initial condition: $x\left( 0 \right)=1$, $y_{1}\left( 0 \right)=y_{2}\left( 0 \right)=y_{3}\left( 0 \right)=0$.

# **Lethal defection**

In our extension, an additional variable, $z$, represents the abundance of dead virus. The dead virus may interfere with the growth of the wild-type $x$ and the mutant $y$ in a frequency-dependent manner, and with rate $\beta$.

We have:

$$\dot{x}=x(bq^{m+n}-a_{j} -\beta z)$$

$$\dot{y}=xbq^{m}\left( 1-q^{n} \right)+y\left( bq^{m}-a_{j}-\beta z \right)$$

$$\dot{z}=\left( x+y \right)b\left( 1-q^{m} \right)-a_{j}z$$

The virus dynamics of the wild-type $x$ along time are shown in **Fig A7**. We notice that for high values of $\beta$, the peak of the virus load becomes a plateau. Moreover, the decrease of the virus load in the clearance phase becomes convex.


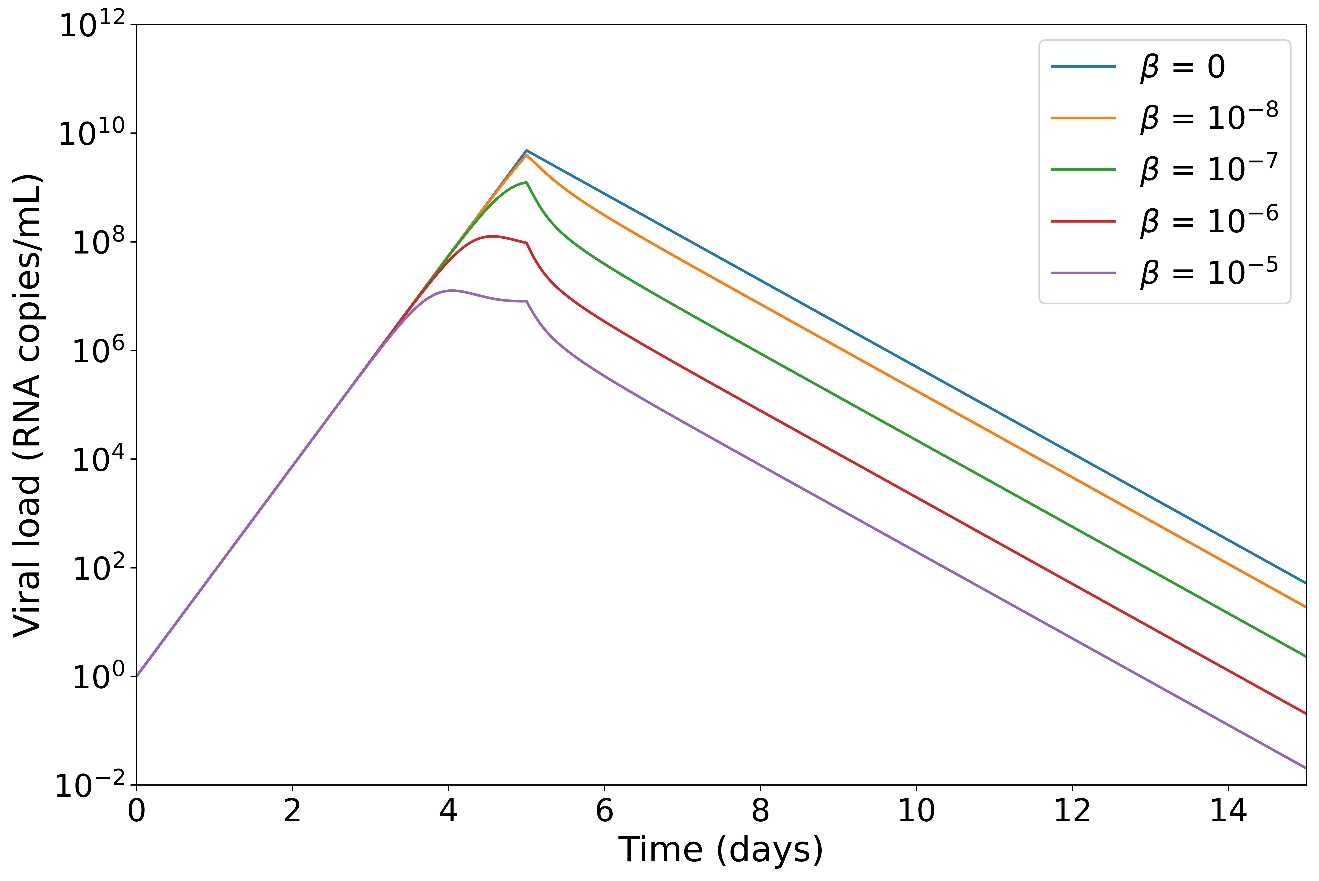


**Fig A7: Time series of wild-type virus** $\boldsymbol{x}$ **for various intensities of interference of the dead virus in the wild-type replication.** With increasing intensity of interference from the dead virus, the peak of the virus load decreases. Parameters: $b=7.61$, $a_{0}=3$, $a_{1}=9$, $u_{0}={10}^{-6}$, $u_{1}=3\cdot{10}^{-6}$, $m=20,000$, $n=87$, $T=5$. Treatment starts after 5 days. Initial condition: $x\left( 0 \right)=1$, $y\left( 0 \right)=z\left( 0 \right)=0$.

Next, we computed the ERF for a grid of parameters and for this model in order to assess how the inclusion of the phenomenon of lethal defection affects evolutionary safety of the treatment. The results are shown in **Fig A8** for $\beta={10}^{-8}$ and in **Fig A9** for $\beta={10}^{-7}$.


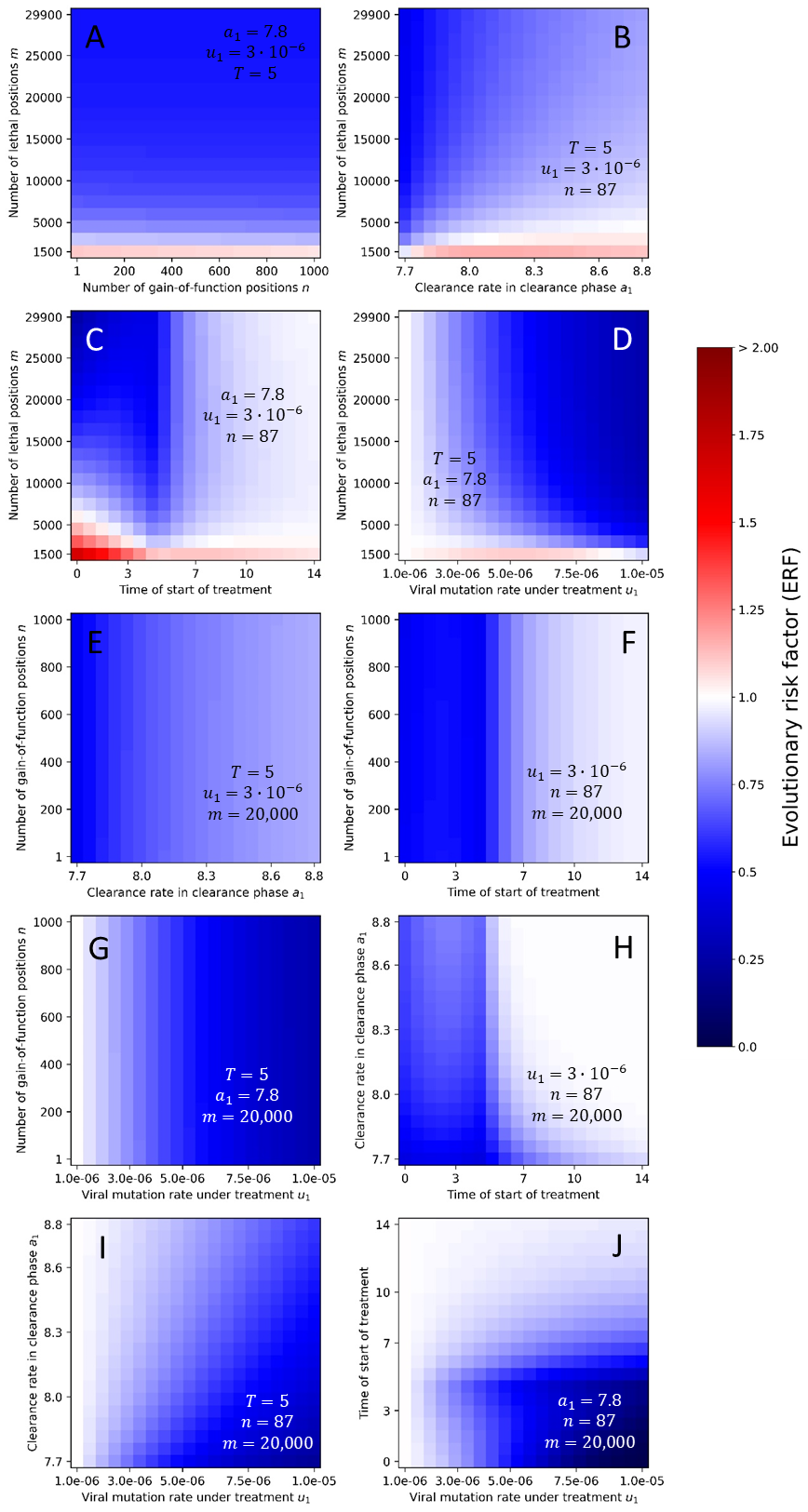


**Fig A8: Evolutionary risk factor for model with lethal defection,** $\boldsymbol{\beta=}\boldsymbol{10}^{\boldsymbol{-8}}$**.** For each pair of parameters, we numerically compute the ERF for a range of values, while the other parameters are fixed. We observe increased evolutionary safety with regards to the case with no lethal defection. Parameters: $b=7.61$, $a_{0}=3$, $T=5$. Initial condition: $x\left( 0 \right)=1$, $y\left( 0 \right)=z\left( 0 \right)=0$.


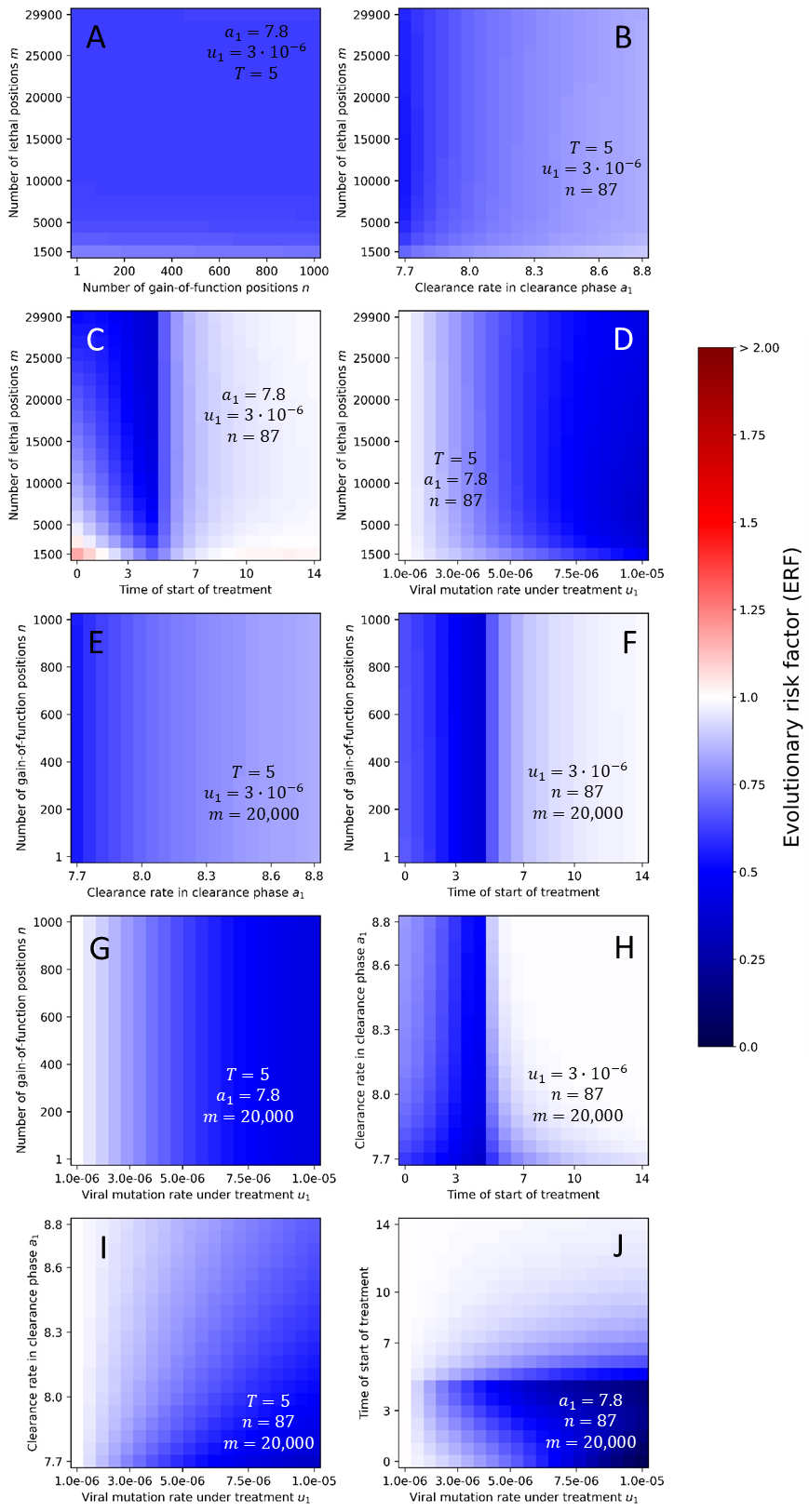


**Fig A9: Evolutionary risk factor for model with lethal defection,** $\boldsymbol{\beta=}\boldsymbol{10}^{\boldsymbol{-7}}$**.** Same as Figure 5, but with $\beta={10}^{-7}$. Parameters: $b=7.61$, $a_{0}=3$, $T=5$. Initial condition: $x\left( 0 \right)=1$, $y\left( 0 \right)=z\left( 0 \right)=0$.

We find that evolutionary safety of a treatment increases when including the interference of defective virus in the replication of the wild-type and potentially concerning mutants. We reasoned that the increase in evolutionary safety of treatment due to incorporation of lethal defection simply stems from the fact that this inhibition of the properly infective sub-population is enhanced further by the treatment-induced generation of the defective sub-population.

**References**

1. Killingley B, Mann AJ, Kalinova M, Boyers A, Goonawardane N, Zhou J, et al. Safety, tolerability and viral kinetics during SARS-CoV-2 human challenge in young adults. Nat Med. 2022;28: 1031–1041. doi:10.1038/s41591-022-01780-9

2. Kim ES, Chin BS, Kang CK, Kim NJ, Kang YM, Choi JP, et al. Clinical Course and Outcomes of Patients with Severe Acute Respiratory Syndrome Coronavirus 2 Infection: a Preliminary Report of the First 28 Patients from the Korean Cohort Study on COVID-19. J Korean Med Sci. 2020;35: e142–e142. doi:10.3346/jkms.2020.35.e142

3. Néant N, Lingas G, Le Hingrat Q, Ghosn J, Engelmann I, Lepiller Q, et al. Modeling SARS-CoV-2 viral kinetics and association with mortality in hospitalized patients from the French COVID cohort. Proc Natl Acad Sci. 2021;118: e2017962118. doi:10.1073/pnas.2017962118

4. Ke R, Zitzmann C, Ho DD, Ribeiro RM, Perelson AS. In vivo kinetics of SARS-CoV-2 infection and its relationship with a person’s infectiousness. Proc Natl Acad Sci U S A. 2021;118. doi:10.1073/PNAS.2111477118

5. Wang Y, Chen R, Hu F, Lan Y, Yang Z, Zhan C, et al. Transmission, viral kinetics and clinical characteristics of the emergent SARS-CoV-2 Delta VOC in Guangzhou, China. eClinicalMedicine. 2021;40. doi:10.1016/J.ECLINM.2021.101129/ATTACHMENT/75433689-84ED-4F43-90B7-C8516E80D9E2/MMC3.PDF

6. Kim JY, Ko JH, Kim Y, Kim YJ, Kim JM, Chung YS, et al. Viral Load Kinetics of SARS-CoV-2 Infection in First Two Patients in Korea. J Korean Med Sci. 2020;35: e86–e86. doi:10.3346/jkms.2020.35.e86

7. He X, Lau EHY, Wu P, Deng X, Wang J, Hao X, et al. Temporal dynamics in viral shedding and transmissibility of COVID-19. Nat Med. 2020;26: 672–675. doi:10.1038/s41591-020-0869-5

8. Jones TC, Guido B, Barbara M, Talitha V, Julia S, Jörn B-S, et al. Estimating infectiousness throughout SARS-CoV-2 infection course. Science (80- ). 2021;373: eabi5273. doi:10.1126/science.abi5273

9. Kawasuji H, Takegoshi Y, Kaneda M, Ueno A, Miyajima Y, Kawago K, et al. Transmissibility of COVID-19 depends on the viral load around onset in adult and symptomatic patients. PLoS One. 2020;15: e0243597. doi:10.1371/journal.pone.0243597

10. Patel K, Dodds M, Gonçalves A, Kamal MA, Rayner CR, Kirkpatrick CM, et al. Using in silico viral kinetic models to guide therapeutic strategies during a pandemic: An example in SARS-CoV-2. Br J Clin Pharmacol. 2021;87: 3425–3438. doi:10.1111/BCP.14718

11. Rodriguez T, Dobrovolny HM. Estimation of viral kinetics model parameters in young and aged SARS-CoV-2 infected macaques. R Soc Open Sci. 2021;8. doi:10.1098/RSOS.202345

12. Ejima K, Kim KS, Ludema C, Bento AI, Iwanami S, Fujita Y, et al. Estimation of the incubation period of COVID-19 using viral load data. Epidemics. 2021;35: 100454. doi:10.1016/J.EPIDEM.2021.100454

13. Kern C, Schöning V, Chaccour C, Hammann F. Modeling of SARS-CoV-2 Treatment Effects for Informed Drug Repurposing. Front Pharmacol. 2021;12. doi:10.3389/fphar.2021.625678

14. Challenger JD, Foo CY, Wu Y, Yan AWC, Marjaneh MM, Liew F, et al. Modelling upper respiratory viral load dynamics of SARS-CoV-2. BMC Med. 2022;20: 1–20. doi:10.1186/S12916-021-02220-0/FIGURES/6

15. Shannon A, Selisko B, Le N-T-T, Huchting J, Touret F, Piorkowski G, et al. Rapid incorporation of Favipiravir by the fast and permissive viral RNA polymerase complex results in SARS-CoV-2 lethal mutagenesis. Nat Commun. 2020;11: 4682. doi:10.1038/s41467-020-18463-z

16. Hay JA, Kennedy-Shaffer L, Kanjilal S, Lennon NJ, Gabriel SB, Lipsitch M, et al. Estimating epidemiologic dynamics from cross-sectional viral load distributions. Science (80- ). 2021;373. doi:10.1126/science.abh0635

17. Kim KS, Ejima K, Iwanami S, Fujita Y, Ohashi H, Koizumi Y, et al. A quantitative model used to compare within-host SARS-CoV-2, MERS-CoV, and SARS-CoV dynamics provides insights into the pathogenesis and treatment of SARS-CoV-2. PLOS Biol. 2021;19: e3001128. doi:10.1371/journal.pbio.3001128

18. Schulte MB, Draghi JA, Plotkin JB, Andino R. Experimentally guided models reveal replication principles that shape the mutation distribution of RNA viruses. Elife. 2015;4. doi:10.7554/eLife.03753

19. Bar-On YM, Flamholz A, Phillips R, Milo R. Sars-cov-2 (Covid-19) by the numbers. Elife. 2020;9. doi:10.7554/eLife.57309

20. Sender R, Bar-On YM, Gleizer S, Bernshtein B, Flamholz A, Phillips R, et al. The total number and mass of SARS-CoV-2 virions. Proc Natl Acad Sci. 2021;118. doi:10.1073/PNAS.2024815118

21. Picardi E, Manzari C, Mastropasqua F, Aiello I, D’Erchia AM, Pesole G. Profiling RNA editing in human tissues: towards the inosinome Atlas. Sci Rep. 2015;5: 14941. doi:10.1038/srep14941

22. Cuddleston WH, Li J, Fan X, Kozenkov A, Lalli M, Khalique S, et al. Cellular and genetic drivers of RNA editing variation in the human brain. Nat Commun. 2022;13: 2997. doi:10.1038/s41467-022-30531-0
