# Supplementary material for: Evolutionary safety of lethal mutagenesis driven by antiviral treatment
Source: PLoS Biol. 2023 Aug 8;21(8):e3002214. doi: 10.1371/journal.pbio.3002214 (PMC10409280; doi:10.1371/journal.pbio.3002214)
Supplement: Attachment — Submitted filename: rebuttal.pdf [file pbio.3002214.s004.pdf]


REVIEWERS' COMMENTS:

Reviewer #1:

In this manuscript Lobinska et al. develop a mathematical model to investigate the balance between efficacy and safety in the use of molnupiravir for SARS-CoV-2. This nucleoside analog is incorporated by the viral polymerase during replication and templates mutations. Given that SARS-CoV-2 and other RNA viruses have mutation rates that are near the maximum tolerable, raising the mutation rate with this drug will reduce viral viability through the accumulation of additional detrimental and lethal mutations. The theoretical downside is that the virus could hit on a mutation that is beneficial - whether through better receptor binding, replication, immune evasion etc. - be transmitted and lead to a new variant of concern. This has been much discussed since the drug's EUA. Sadly, much of the discussion ignores a long history of study of lethal mutagenesis - albeit in other viral systems - which has explored these issues on a theoretical and experimental basis. Some of that work is cited by the authors. The authors develop a model that incorporates various parameters that are known (or at least bounded) for SARS-CoV-2, including, but not limited to: mutation rate, fraction of lethal mutations, growth rate, clearance rate. The parameter space is explored and the boundaries defined where the goals of viral elimination and avoidance of harmful variants are achieved.

As a virologist, and not a mathematical modeler, I found this manuscript a bit dense and difficult to read. This may be how manuscripts in the subfield are written and presented, but it could detract from the readership at a general interest journal at PLOS Biology.

Thank you for your comment. We have made sure that the mathematics is kept to the minimum in the main text, and the reader is directed to the Methods section for most derivations. Moreover, additional analyses are introduced as Supplementary Figures, and their description in the main text is likewise kept to the strict minimum.

We hope that you will find the reading of the manuscript much easier.

A larger issue is that the model does not account for much of the biology/mechanism of lethal mutagenesis. In this way, it is a bit simplistic in its assumptions and may not really be as informative about the safety and efficacy of lethal mutagenesis as the authors suggest.

Thank you for your comment. We have explored all of the points that you raised in your assessment. Below is a detailed account of the new analyses proposed here, we believe that the theory is now more comprehensive and can be applied in future to a broader set of viruses and mutagenesis drugs.

A few considerations along these lines:

1. Replication mode and number of mutations per generation/cellular infection cycle. The authors should consider the complicating factor of mode of replication. Stamping vs. linear replication (see discussion in cited Sanjuan papers) and whether mutations occur in minus vs. plus strand synthesis can have a profound effect on the number of mutations per genome. See PMID: 25635405. Similarly, RNA editing from APOBEC, which appears to be quite common in SARS-CoV-2, will lead to a higher mutation rate in vivo than the estimates given for the virus passaged in vitro. These issues change the expected number of mutations per genome, perhaps beyond the assumptions of the model.

Thank you for your comment.

Your comment mentions three mechanisms that affect the distribution of mutants generated during an infection cycle: (i) the mode of replication: stamping vs. linear replication; (ii) the plus-minus-plus strand replication cycle; and (iii) RNA editing within the cell.

In our model, the expected number of mutations per genome depends on the mutation rate μ. In the section "Values of parameters" we provide two estimates for μ from the literature.

The first estimate is based on RNA sequencing of infected cells. The sequencing was performed when more than 10% of the cells were involved in syncytia. Much of the sequencing material is therefore expected to have come from mature virions. The second estimate was obtained via RNA sequencing of the virus suspension of cells in culture. Both of the estimates are very similar in value: $\mu \approx 10^{-6}$. Therefore, we have used $\mu = 10^{-6}$ in the main text of our paper. These estimates are based upon the number of mutants found in the cells, or in the virus suspension, after the replication of a wild-type within a cell. Hence, processes such as the mode of replication within the cell (stamping or linear replication), the plus-minus-plus cycle, and RNA editing are already included in the estimates provided by [1–4]. This is now addressed in the revised version in page 6 lines 155-160 and in Supplementary file "Estimating the mutation rate".

We do acknowledge in the revision that the presence of these processes has a profound influence on the distribution of mutants in the virus population within a patient. Below, we detail an analysis of the influence of the mode of replication and the plus-minus-plus replication cycle on the distribution of mutants. In short, we find that the distributions of the number of mutants do look very different depending on the mode of replication and the number of template minus strands. This is in agreement with the studies you cited.

However, the shape of these distributions, even though affected by factors such as the mode of replication, will not be affected by the presence or absence of the drug treatment, which is the variable of focus in our study. Moreover, the expected number of mutations is not affected neither by the mode of replication nor by the number of negative-strand templates during the plus/minus/plus replication cycle (see details below).

The expected number of mutations per genome is proportional to $u_0 = 1 - q_0$ (without treatment) or to $u_1 = 1 - q_1$ (with treatment). Hence, it will not be affected by the mode

of replication or the number of template genomes, which affect the distribution of the number of mutants but not their mean.

Stamping vs. linear replication

We simulated the replication of one virion to obtain a 100 progeny virions assuming either stamping or linear replication over three generations within the same cell. A cartoon illustrating these two modes of replication is presented in **Figure 1**.

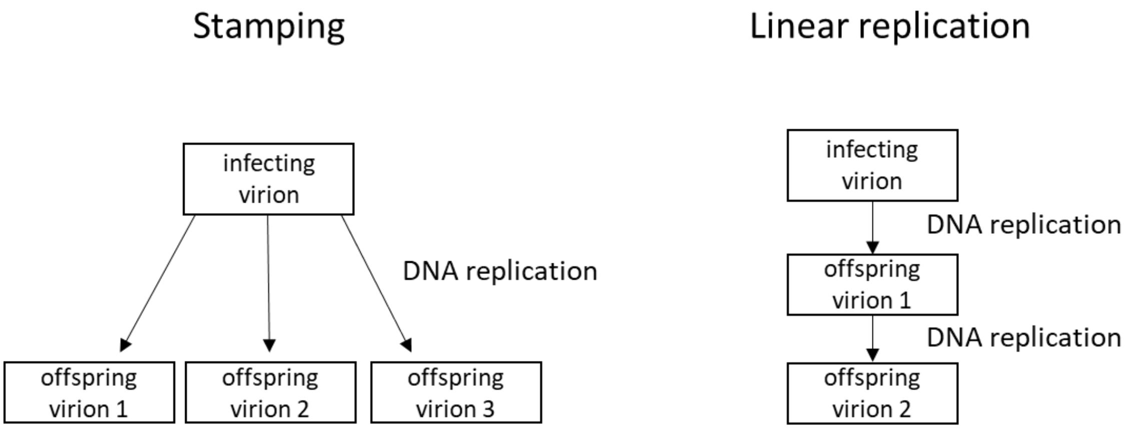

Figure 1: Schematic representation of two modes of replication: stamping and linear replication. During stamping, the genome of the infecting virion serves as a template for the synthesis of all offspring virions. During linear replication, the genomes of the offspring virions that have already been synthetized can serve as templates for the replication of additional viral offspring. Each mode of replication can potentially lead to a different distribution of the number of mutants arising from an infection event.

Stamping refers to replication of the 100 progeny virions using the genome of the virion that entered the cell as the template. We simulated it by randomly sampling a 100 times from a binomial distribution with parameters $n = 1$ and $p = \mu$, where μ is the mutation rate.

With linear replication, the virion that entered the cell is used as a template to generate the first generation of virions, and these can be then used themselves as templates for the second generation of virions. This process can extend over up to 3 generations within a cell [5].

To simulate this mode of replication, we randomly sampled the number of mutants in the first generation from a binomial distribution with parameters $n = 1$ and $p = \mu/3$, where μ is the mutation rate, similarly to what we performed for the stamping mode. We divide the mutation rate by 3 to correct for the three replication events occurring within the cell.

However, instead of sampling 100 progeny virions, we sampled 4 first generation virions, a number we chose arbitrarily.

We then chose 20 as the number of virions in the second generation and performed a random sampling with replacement of the first generation mutants to obtain the parents' of the second generation virions. We then simulated replication through the random sampling from the binomial distribution with parameters $(1, \mu/3)$. The number of mutants in the second generation was the sum of the mutations present in their parent and the mutations acquired during replication.

Lastly, we repeated this procedure to obtain the third and last generation: a 100 parent virions were chosen through random sampling with replacement of the second generation, and replicated was simulated through random sampling from a binomial distribution with parameters $(1, \mu/3)$. The number of mutations in the progeny was the sum of the number of mutations in the parent and the number of mutations generated during replication.

For each mode of replication – stamping or linear replication over three generations – we plotted the distribution of the number of mutants for 5,000,000 simulation runs, and for several mutation rates. The lowest mutation rate, $\mu = 10^{-6}$ per infection cycle, represents a situation with no mutagenic treatment. Higher mutation rates represent treatments inducing different levels of mutagenesis in the virus.

We observe that the distributions are very different between the two modes of replication (see **Figure 2**). However, the shape of the distributions looks similar between the different levels of the mutation rates for each mode of replication separately. Importantly, the average number of mutants is unaffected (see **Figure 3**).

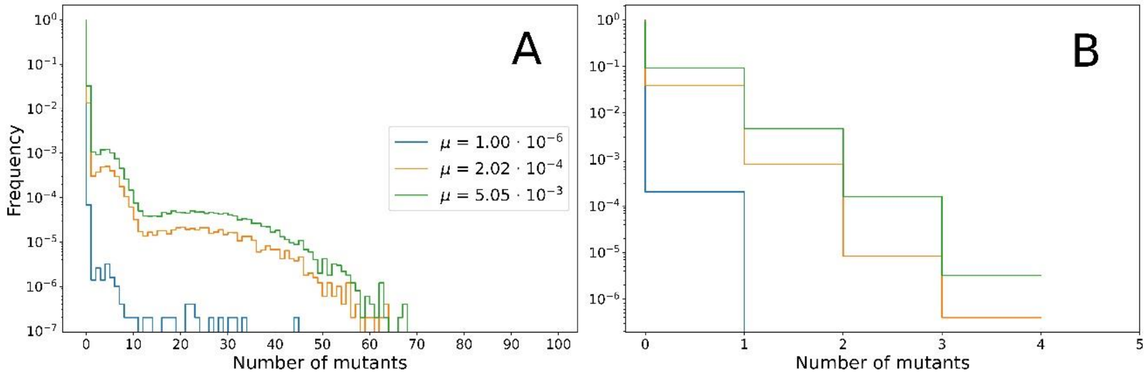

Figure 2: Distribution of the number of mutants depending on the mode of replication: stamping (panel A) or linear replication with three generations within one cell (panel B). Although the distribution of the number of mutants differs depending on the mode of replication, the shape of the distributions is similar for different mutations rates.

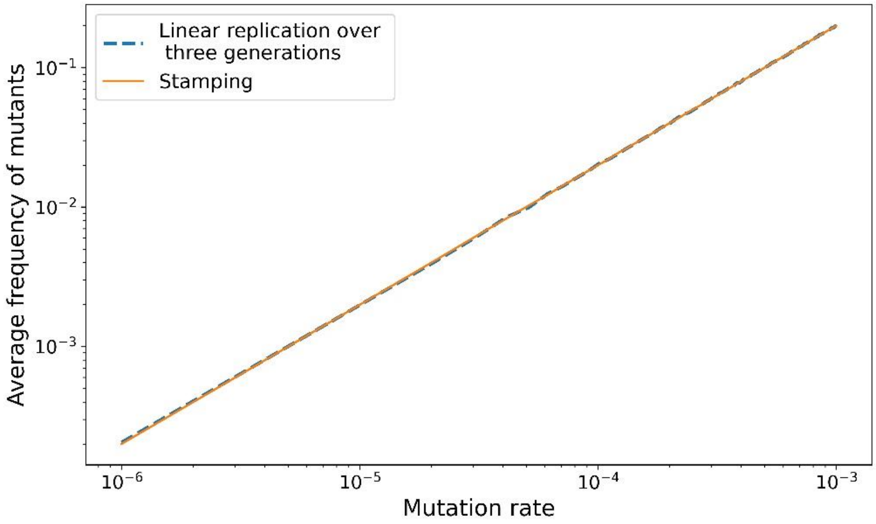

Figure 3: Average frequency of mutants depending on the mutation rate. It is identical for both of the considered modes of replication. Since our model is concerned with the expected number of mutations per patient, the mode of replication does not affect our conclusions.

Plus-minus-plus replication cycle

SARS-CoV2 is a plus-strand RNA virus. In order to replicate, it first synthetizes an intermediate minus strand, which then serves as a template for offspring plus-strands. A cellular infection event gives rise to about 100 progeny virions [3]. Within each patient, between 10^4 and 10^6 cells will be infected [6].

We constructed a simulation of a cellular infection event, and compute the distribution of mutants within a patient depending on the number of intermediate minus strands, which we denote by c_1. The variable c_1 can range between 1, if all progeny virions are synthetized from the same minus template, and 100, if each progeny virion in synthetized from a different minus template. To the best of our knowledge, the value of c_1 for SARS-CoV is not known.

Our simulation runs as follows. First, we sample the number of mutations that occurred during the synthesis of the minus strand from the plus strand. We use a binomial distribution with parameters $n = L$ and $p = 10^{-6}$, and sample c_1 random variables. L is the length of the genome. In practice, we do not expect more than 2 mutations per infection cycle. Hence, to reduce computation time, we used $L = 2$. We thus obtain the distribution of the number of minus strands that are wild-type, single mutants or double mutants. We expect $2\ (1 - p)\ p\ c_1$ template strands to be single mutants.

We then use this distribution to sample c_2 strands that will serve as templates to synthetize plus strands. We neglect the probability of back mutations. Hence, if a mutation occurred during the synthesis of the minus strand, it will be ensured to propagated to all plus strands

synthetized from that mutant template RNA molecule. Hence, we expect $2\,(1-p)\,p\,c_2$ plus strands to be synthetized from a minus strand that is a single mutant.

The number of mutations occurring during the synthesis of plus strands from each parent minus strands can be obtained through another sampling of a binomial distribution, with parameters $n = L, p = 10^{-6}$ and size c_2. Out of $(1-p)\,c_2$ plus strands that are synthetized from a wild-type template, $p\,c_2$ will become at least single mutants. The total number of mutations in the cell will be the sum of the number of mutations in the template strand used for the synthesis of each c_2 of plus strands and the number of mutations that occurred during the synthesis of each c_2 plus strand from the template minus strand. Hence, we expect a total of $c_2\,p + (1-p)\,p\,c_2$ mutant virions in the cell. Note that this expression is independent from c_1.

We simulated this model for $c_1 = 1$, $c_1 = 25$, $c_1 = 50$ and $c_1 = 100$. In **Figure 4**, we plotted the histograms of the number of single mutants obtained from each infected individual.

Although the variance of the number of single mutants per patients varied considerably, the sum of the number of single mutants remained constant, regardless of the value of c_1.

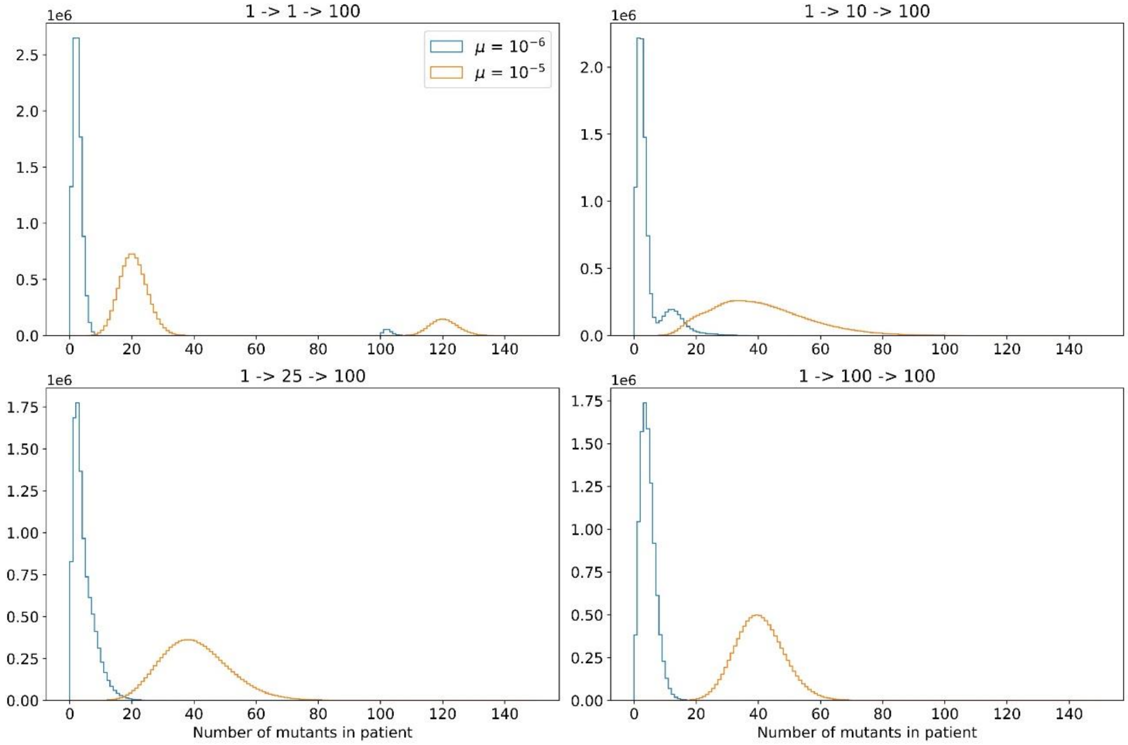

Figure 4: Distribution of the number of mutants in the progeny genomes depending on the number of negative-strand templates. Although the distributions are very different, they all result in the same average number of mutant in patient. Moreover, the shape of the distribution is conserved for different mutation rates, representing absence or presence of treatment.

Hence, the expected number of mutations is invariant to whether the mutations occur in the plus or in the minus strand.

This analysis of replication mode is now introduced in the main text, page 6 lines 150-155, and a supplemental file “Estimating the mutation rate” is now added to the paper with this entire analysis.

RNA editing

As mentioned before, mutations stemming from RNA editing are likely to have been included in the measured mutation rate which we used. This is because these estimates were established in cell culture already capturing their RNA editing levels.

However, we acknowledge that RNA editing can be present at various levels across cell types and cell conditions [7,8].

Note that we already considered the possibility that our estimate for the mutation rate underestimated the true value of the mutation rate without treatment. In the main text, we consider the mutation rate μ_0 without treatment to be 10^{-6} per nucleotide per cellular infection cycle. In **Supplementary Figure 7**, we consider $\mu_0 = 5 \cdot 10^{-5}$. In **Supplementary Figure 8**, we consider $\mu_0 = 10^{-5}$. Following this comment, we now explicitly mention that RNA editing, with its potential variable extent across cell types, could module the apparent mutation rate of the virus.

2. From my reading, the model basically considers the fraction of lethal and non-lethal mutations and the likelihood of the virus making a mutation in either class given its mutation rate. It reads as if any non-lethal mutation is considered potentially beneficial (could lead to a VOC) within the spike RBD or within the rest of the genome either through its direct effects or through establishing a road to additional mutations via higher order epistasis. To me, this ignores some of the recognized complexity. Non-lethal deleterious mutations don't appear to be considered (and also mutations that can reduce fitness through epistasis as well). In the vast majority of situations, these would be outcompeted or cleared faster rather than unmutated wild type viruses. Put another way, the ~60% of mutations with a fitness value of 0.1-0.9 would need to explicitly be considered (see also PMID 27571422 in addition to cited papers from Sanjuan).

Thank you for your comment.

We have now extended our model to explicitly take into account non-lethal deleterious mutations.

In addition to the abundance of wild-type, x, and the abundance of the potentially concerning mutant, y_1, we now also consider the abundance of deleterious mutants, y_2, and the abundance of mutants that are both deleterious and potentially concerning, y_3.

Mutation in any one of n_1 positions leads from x to y_1. Mutation in any one of n_2 positions leads from x to y_2. Mutation in any one of n_3 positions leads from x to y_3. Mutation in any one of $n_2 + n_3$ positions leads from y_1 to y_3. Mutation in any one of $n_1 + n_3$ positions leads from y_2 to y_3. Back mutations are ignored. As in our original model, mutation in any one of m mutations is lethal. Deleterious mutations have a birth rate b' which is less than b. The subscript j in a_j denotes the absence ($j = 0$) or presence ($j = 1$) of an adaptive immune response. Let $M = m + n_1 + n_2 + n_3$. Virus dynamics are now described by

$$\dot{x} = x(bq^M - a_j)$$

$$\dot{y_1} = xbq^{M-n_1}(1 - q^{n_1}) + y_1(bq^{M-n_1} - a_j)$$

$$\dot{y_2} = xbq^{M-n_2}(1 - q^{n_2}) + y_2(b'q^{M-n_2} - a_j)$$

$$\dot{y_3} = xbq^m(1 - q^{n_3}) + y_1bq^m(1 - q^{n_2+n_3}) + y_2b'q^m(1 - q^{n_1+n_3}) + y_3(b'q^m - a_j)$$

We computed the abundance of each category of virus over time and plotted it in **Figure 5**.

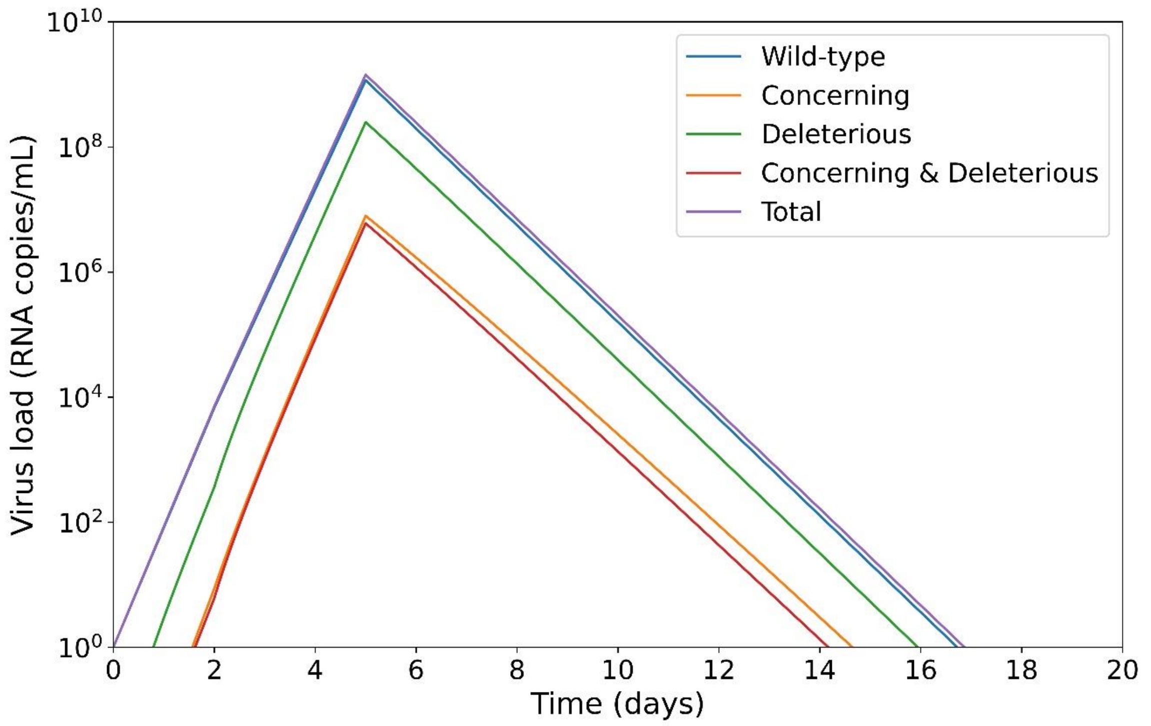

Figure 5: Time series of total virus ($v = x + y_1 + y_2 + y_3$), wild-type virus (x), concerning virus (y_1), deleterious virus (y_2), concerning and deleterious virus (y_3). Parameters: $b = 7.61$, $b_1 = 0.9 \cdot b$, $a_0 = 3$, $a_1 = 8.8$, $u_0 = 10^{-6}$, $u_1 = 3 \cdot 10^{-6}$, $m = 20{,}000$, $n_1 = 87$, $n_2 = 6713$, $n_3 = 100$, $T = 5$. Treatment starts after 2 days. Initial condition: $x(0) = 1$, $y_1(0) = y_2(0) = y_3(0) = 0$.

The wild-type virus is always the major category. Deleterious mutants are roughly an order of magnitude less abundant than the wild-type at peak point. Concerning mutants are roughly three orders of magnitude less abundant than the wild-type. Lastly, deleterious concerning mutants are roughly two and a half orders of magnitude less abundant than the wild-type.

We then computed the ERF for a range of values of the number of lethal positions m and of the clearance rate in the clearance phase a_1. The number of concerning positions is $n_1 = 87$, as previously. Additionally, we consider arbitrarily $n_3 = 100$ positions that are both deleterious and concerning. About 10% of positions in the genome are assumed that be neutral. SARS-CoV2's genome is 29,900 nt in length, hence about 3000 positions are estimated to be neutral when mutated. Therefore, the number of positions that are deleterious when mutated is $n_2 = 29{,}900 - 87 - 100 - m$. We plotted the results in **Figure 6**.

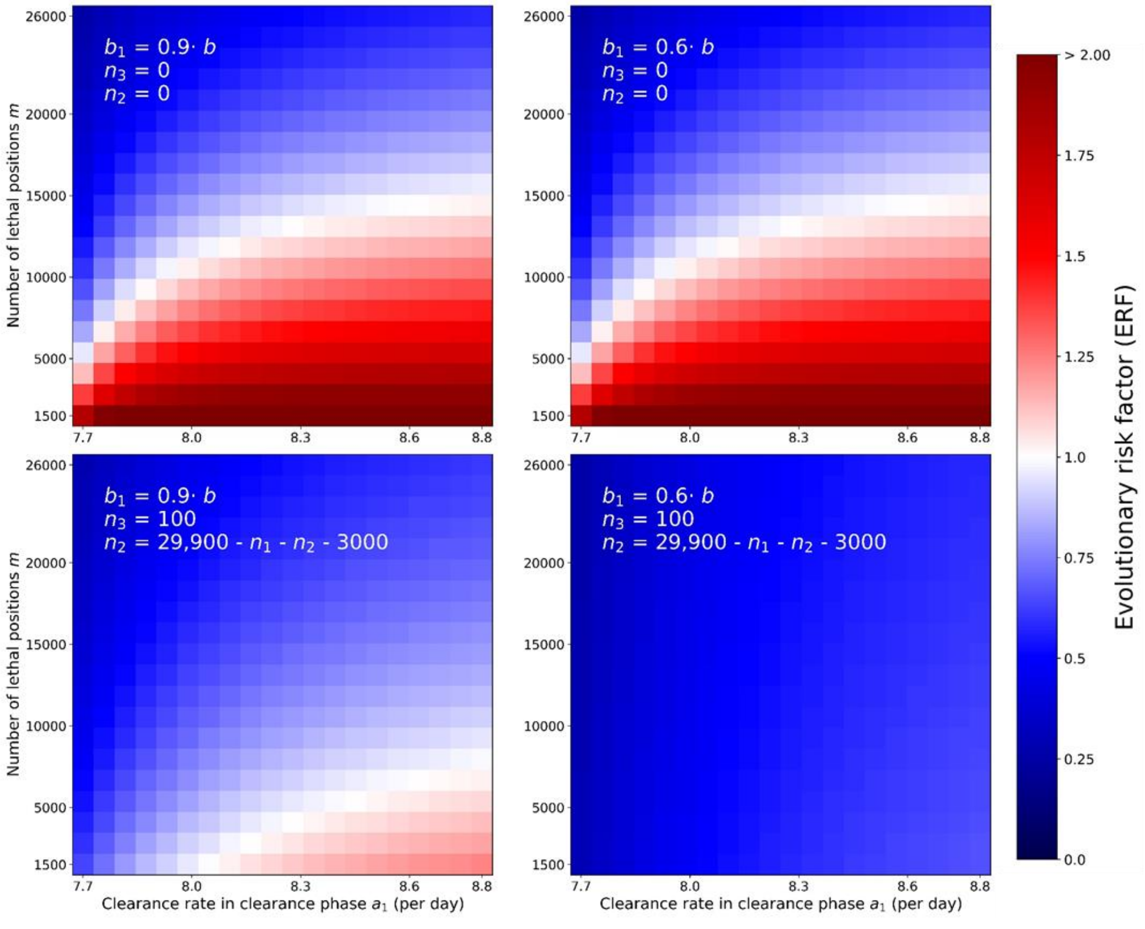

Figure 6: Comparison of the model with and without considering non-lethal deleterious mutations. Including non-lethal deleterious mutations increases the evolutionary safety of the treatment. The ERF is computed as the cumulative sum of the concerning mutant y_1 and the concerning and deleterious mutant y_3 with treatment, normalized by the corresponding sum without treatment. Parameters: $b = 7.61$, $a_0 = 3$,

$u_0 = 10^{-6}$, $u_1 = 3 \cdot 10^{-6}$, $n_1 = 87$, $T = 5$. Treatment starts after 2 days. Initial condition: $x(0) = 1$, $y_1(0) = y_2(0) = y_3(0) = 0$.

We conclude that extending our model to include non-lethal deleterious mutations results in higher evolutionary safety than when we neglect them. This issue and analysis are now highlighted in page 15 line 398-404 and a full account of the extended model and the results are in the Supplementary file "Non-lethal deleterious mutations".

3. As in 2, a non-lethal mutation is considered as a candidate for a VOC when it is clear that most VOC emerge after a process that entails a number of mutations arising over a significant period of time (given the known within-host and global rates of evolution). Clearly, the virus would need to hit on the right mix of non-lethal mutations in a short period of time.

Thank you for your comment.

Variants of concern (VoC) are defined by the World Health Organization (WHO) as variants with increased virulence, transmissibility or resistance to existing treatments and vaccines. In our study, we first attempted to estimate the number of mutations in the viral genome that, when mutated, could increase the fitness of the virus. We acknowledge that a mutant with increased fitness is not necessarily a variant of concern as per the definition of the WHO. Hence, we welcome your comment as highly pertinent and reworded all instances from "variant of concern" to "mutant" or "potentially concerning mutant".

You are also correct in pointing out that many VoCs are not single mutants, but rather are multiple-step mutants that evolved over a period of time, and potentially, multiple hosts. As you pointed out, a mutant is highly unlikely to become a VoC after a short period of time. However, widespread use of mutagenic treatments can increase the standing variation of the virus in the population. This variation can facilitate the evolution of VoC. Therefore, in our study, we choose a conservative definition of evolutionary safety. A treatment is considered evolutionarily safe if no mutant is generated in higher amounts under treatment.

Hence, it is not that we consider every non-lethal mutation as a candidate for a VoC. Rather, we adopt a stringent requirement for evolutionary safety, namely that all the quantity of all generated mutants with treatment be lower or equal than without treatment. This is now explained in the revised Discussion, page 20 lines 514-517.

4. Other factors that influence the dynamics of mutation accumulation and spread (in VOC and viruses in general) are not considered. First, there is a considerable amount of genetic drift involved in mutations increasing in frequency in vivo (from newly generated mutation to a frequency at which it can plausibly have an effect on phenotype and transmit).

Thank you for your comment.

You are right that considering genetic drift introduces a layer of stochasticity which has not been included in our model. This randomness may indeed affect the dynamics of the mutant frequencies as it appears and in the initial stages of its rise in frequency. To address

such this we now implement the Gillespie algorithm to implement a stochastic version of our model. Due to the large number of events (including more than 10^{10} birth events), we used tau-leaping (REF) in order to reduce computational time. We computed the proportion of runs, out 1000, where evolutionary safety is achieved for a range of values for the number of lethal positions m and the clearance rate in the clearance phase a_1. The results are shown in **Figure 7**. We introduce the stochastic version of the model in page 13 lines 353-356 and **Supplementary Figure 12**.

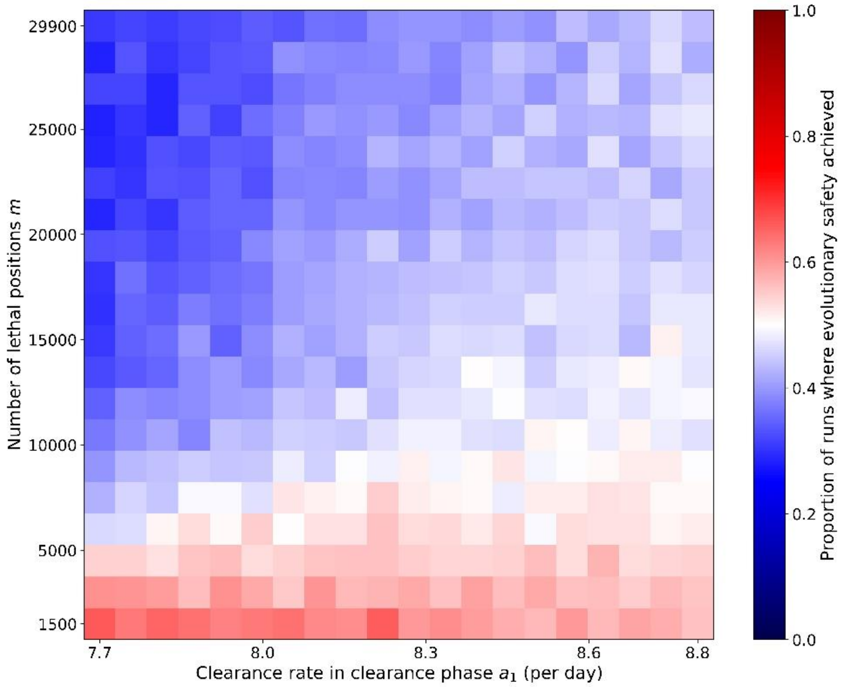

Figure 7: Probability that evolutionary safety is achieved given a value of the number of lethal positions m and the clearance rate in clearance phase a_1. For each pair of values, we ran 1000 runs of the Gillespie algorithm. The value plotted is the proportion of runs where the cumulative sum of the potentially concerning mutant with treatment was higher than the cumulative sum of the potentially concerning mutant without treatment, that is, the proportion of runs where the ERF exceeded 1. Treatment starts at peak of the virus load. Parameters: $b = 7.61$, $a_0 = 3$, $n = 87$, $T = 5$, $u_0 = 10^{-6}$, $u_1 = 3 \cdot 10^{-6}$. Initial condition: $x(0) = 5$, $y(0) = 0$.

We report a good agreement between the deterministic and the stochastic version of the model. In particular we note that range of parameter plane for which evolutionary safety is not achieved in the deterministic model largely overlaps with the range in which evolutionary safety is not achieved by the stochastic version.

Second, through the process of lethal defection (first described in PMID: 15767582), mutated viral genomes can act as dominant negatives and interfere with the replication and progression of non-lethally mutated genomes.

Thank you also for raising the subject of lethal defection, which was indeed not included in our original model.

We now address this important issue at the level of Introduction, where the above mentioned paper is properly introduced (page 2 l.40), and also by an extended version of our model that considers lethal defection. In our extension, an additional variable, z, represents the abundance of dead virus. The dead virus may interfere with the growth of the wild-type x and the mutant y in a frequency-dependent manner, and with rate β.

We have:

$$\dot{x} = x(bq^{m+n} - a_j - \beta z)$$

$$\dot{y} = xbq^m(1 - q^n) + y\left(bq^m - a_j - \beta z\right)$$

$$\dot{z} = (x + y)b(1 - q^m) - a_j z$$

The virus dynamics of the wild-type x along time are shown in **Figure 8**. We notice that for high values of β, the peak of the virus load becomes a plateau. Moreover, the decrease of the virus load in the clearance phase becomes convex.

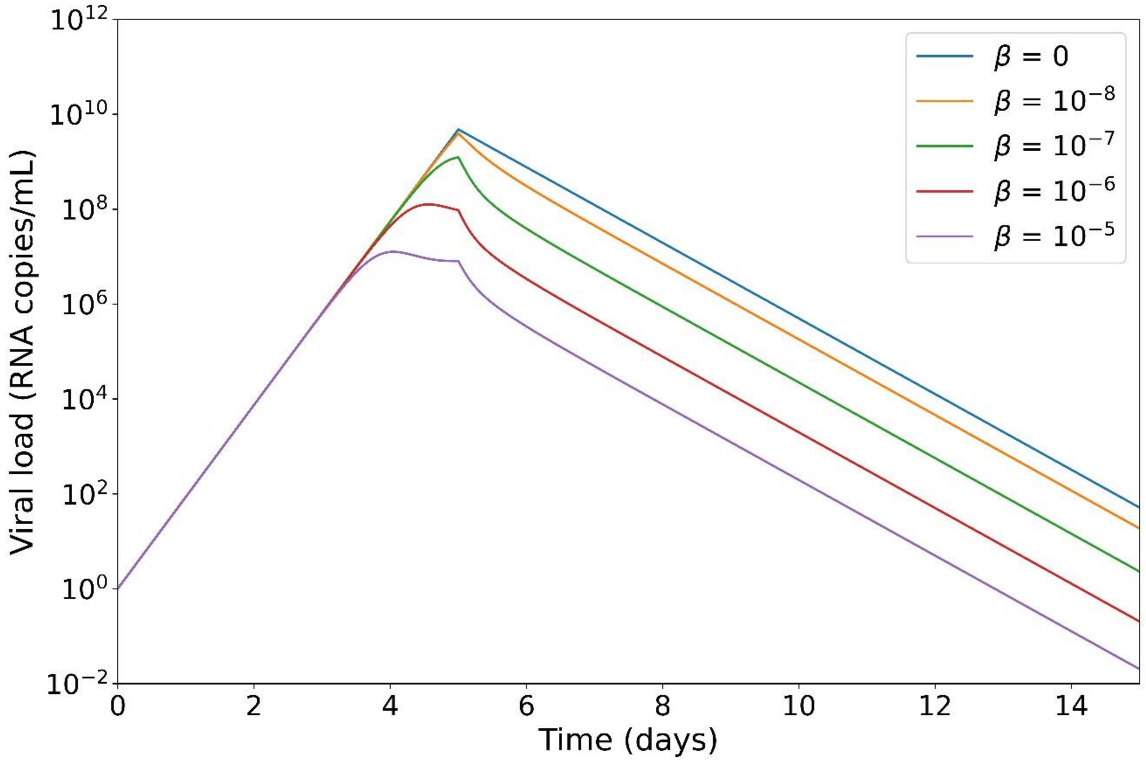

Figure 8: Time series of wild-type virus x for various intensities of interference of the dead virus in the wild-type replication. With increasing intensity of interference from the dead virus, the peak of the virus load decreases. Parameters: $b = 7.61$, $a_0 = 3$, $a_1 = 9$, $u_0 = 10^{-6}$, $u_1 = 3 \cdot 10^{-6}$, $m = 20{,}000$, $n = 87$, $T = 5$. Treatment starts after 5 days. Initial condition: $x(0) = 1$, $y(0) = z(0) = 0$.

Next, we computed the ERF for a grid of parameters and for this model in order to assess how the inclusion of the phenomenon of lethal defection affects evolutionary safety of the treatment. The results are shown in **Figure 9** for $\beta = 10^{-8}$ and in **Figure 10** for $\beta = 10^{-7}$.

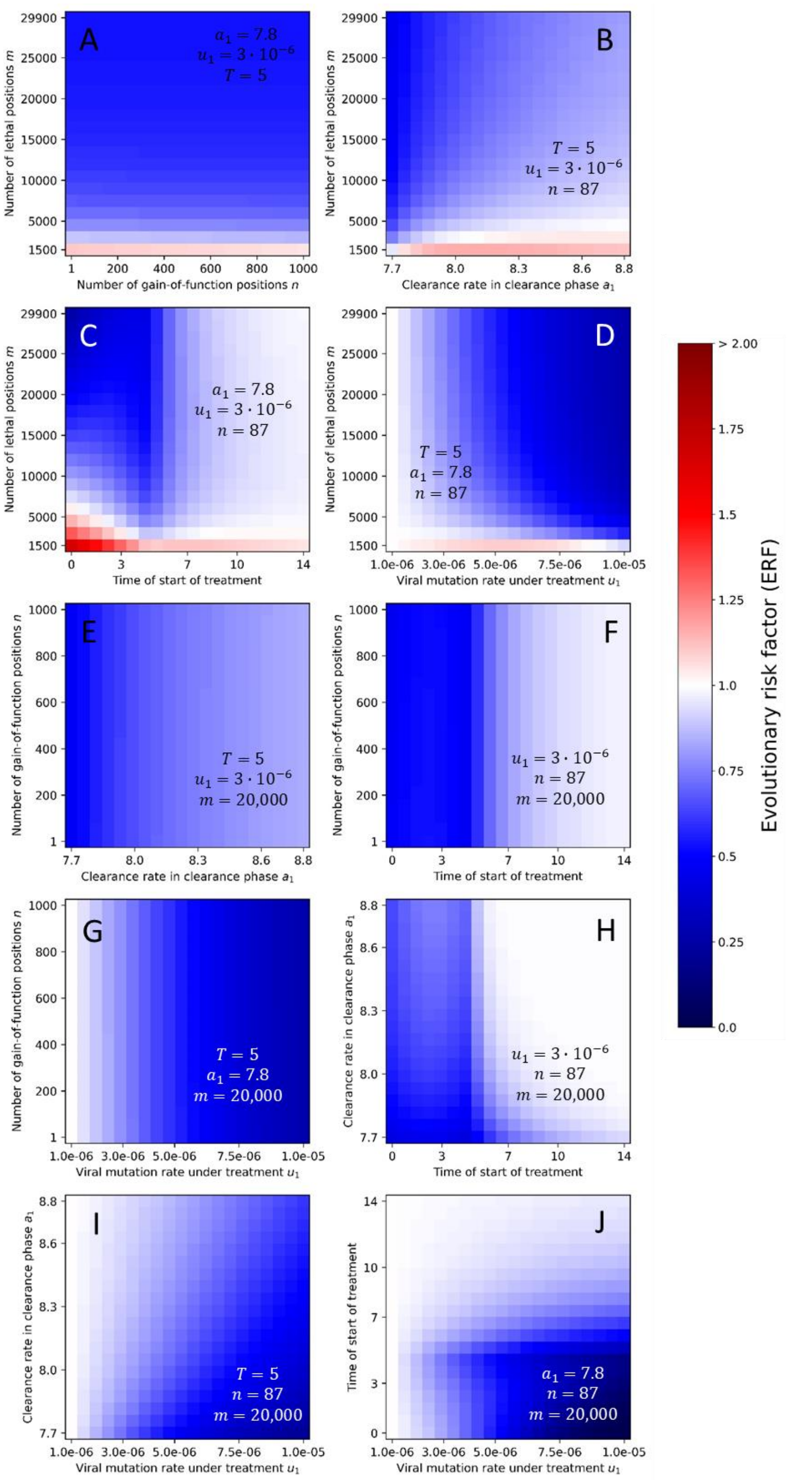

Figure 9: Evolutionary risk factor for model with lethal defection, $\beta = 10^{-8}$. For each pair of parameters, we numerically compute the ERF for a range of values, while the other parameters are fixed. We observe increased evolutionary safety with regards to the case with no lethal defection. Parameters: $b = 7.61$, $a_0 = 3$, $T = 5$. Initial condition: $x(0) = 1$, $y(0) = z(0) = 0$.

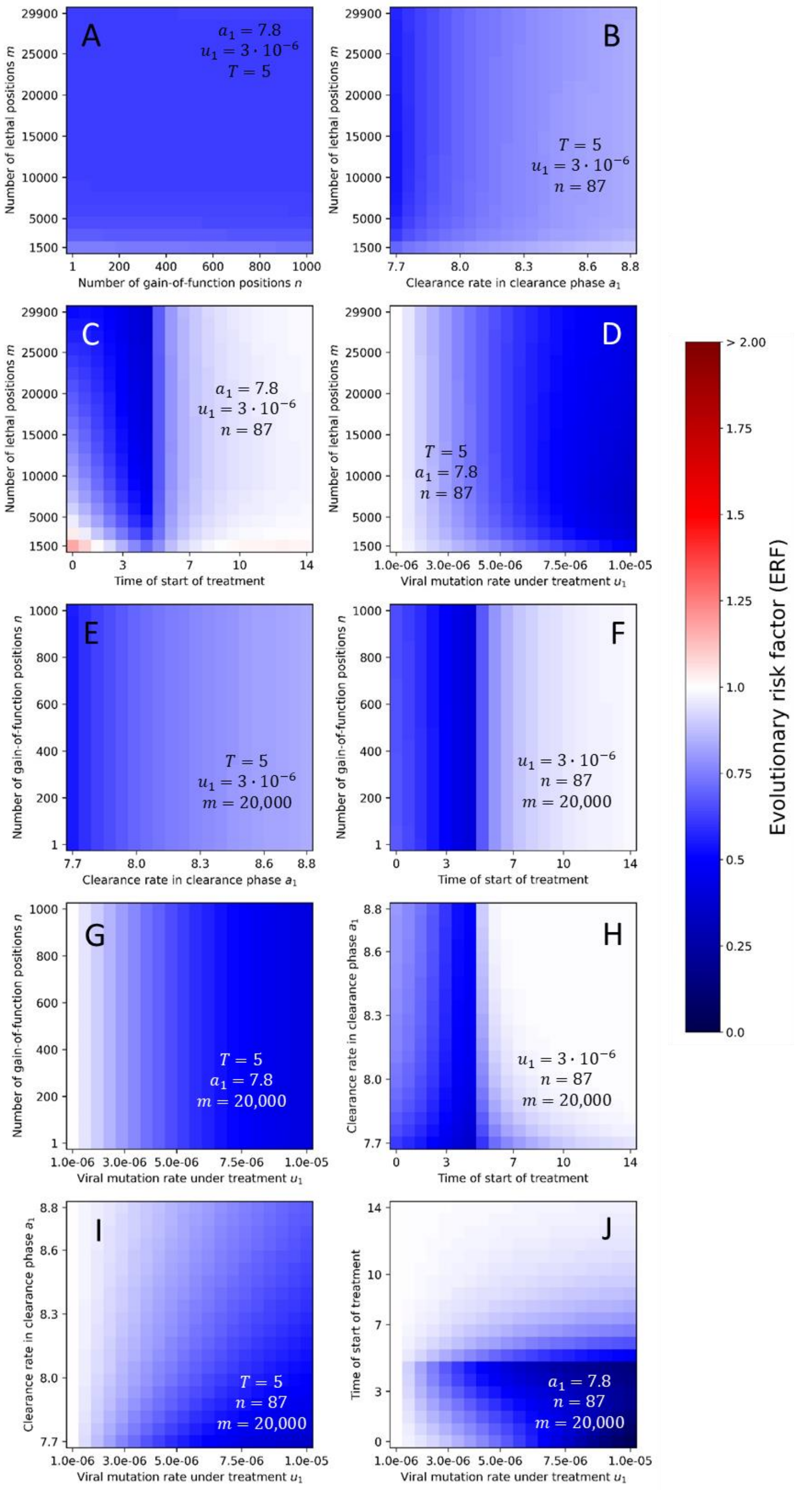

Figure 10: Evolutionary risk factor for model with lethal defection, $\boldsymbol{\beta = 10^{-7}}$. Same as Figure 5, but with $\beta = 10^{-7}$. Parameters: $b = 7.61$, $a_0 = 3$, $T = 5$. Initial condition: $x(0) = 1$, $y(0) = z(0) = 0$.

We find that evolutionary safety of a treatment increases when including the interference of defective virus in the replication of the wild-type and potentially concerning mutants. We reasoned that the increase in evolutionary safety of treatment due to incorporation of lethal defection simply stems from the fact that this inhibition of the properly infective sub-population is enhanced further by the treatment-induced generation of the defective sub-population. This new analysis of the lethal defection scenario is now introduced in the revised paper, see page 15-16, lines 404-412, and in a Supplementary file “Lethal defection”.

I recognize that some of these factors may be hard to model or incorporate here. However, they are real phenomena and could significantly impact the interpretations and conclusions of the model presented here.

We agree that all factors that you suggested are crucial for our extended model and the validity of its conclusions. We enthusiastically recognize that the extended model is much more comprehensive and realistic now, and that it also lays the foundations for a future application to additional pathogens and treatments.

Minor Points

Line 37 - it is unclear why the authors coin a new term "death by mutagenesis" when the term "lethal mutagenesis" has been used by the field for 30 years.

Thank you for your comment. We have replaced all instances of “death by mutagenesis” by “lethal mutagenesis”. See for example l. 58, 86, and 268.

Line 52-53 - while terms like error catastrophe and error threshold are often used in the literature (due to their origins in quasi species theory), these aren't directly applicable to the process of lethal mutagenesis. For models and discussion, see Bull et al. PMID: 17202214

Thank you very much for your comment. We now provide references to the discussion of error catastrophe and error threshold in the context of the lethal mutagenesis l. 116, including the paper that you mention.

Line 75 - "posology"?

Thank you for your comment. We have replaced “posology” with “dosage” in l.80.

Reviewer #2:

In "Evolutionary safety of death by mutagenesis", authors investigate "evolutionary safety" of drugs whose mechanism of action is to induce mutations during viral replication.

I was asked to examine specifically the mathematics used in this study. As such I began with the Methods (starting after the references, page 33 of my pdf).

The model (eqs 5a-b) is very simple but in line with models commonly used to get a foothold into such problems. x represents the wild type population, y any and all mutations, and v=x+y the total virus. I am curious about the assumption that the peak time is independence from the treatment - a treatment initiated before peak would affect the peak timing and magnitude, wouldn't dependence on cumulative virus perhaps be more sensible? - but that can wait until the mathematics are corrected.

The first problem is a mistake in the integration in equation 11. The answer should be V- = exp((b*q^m-a1)*T)*exp((b*q^m-a0)*T)/(a1-b*q^m).

The factor exp((b*q^m-a1)*T) is missing.

I don't think that this is a typo b/c the error is repeated, in eqs (12), (13), (14), (16), (17), (18) (note: I stopped after equation 19, as it seemed that the work was built upon incorrect expressions).

Thank you for your comment and the very thorough review of the paper and the mathematical development. We have checked Eq. 11. The problem you are pointing out is the consequence of a typo in the lower bound for the integration. The integration parameter t should run from 0 to infinity and not from T to infinity. The exponential decline starts with initial condition v_T and decays from this quantity down to zero. The correct Equation 11 reads:

$$V^- = \int_0^\infty v(t)dt = \frac{v_T}{a_1 - bq^m} = \frac{1}{a_1 - bq^m} e^{(bq^m - a_0)T} \qquad (11)$$

As you can see, the right-hand side of this equation is unchanged and therefore all subsequent results are correct. We have corrected our typo in the manuscript. Thank you for pointing this out.

To further ascertain the correctness of Eqs. (11), (12), (13), (14), (16), (17) and (18), we compared our expressions with their numerical values obtained with the Euler method.

The results are shown below in **Figure 11**.

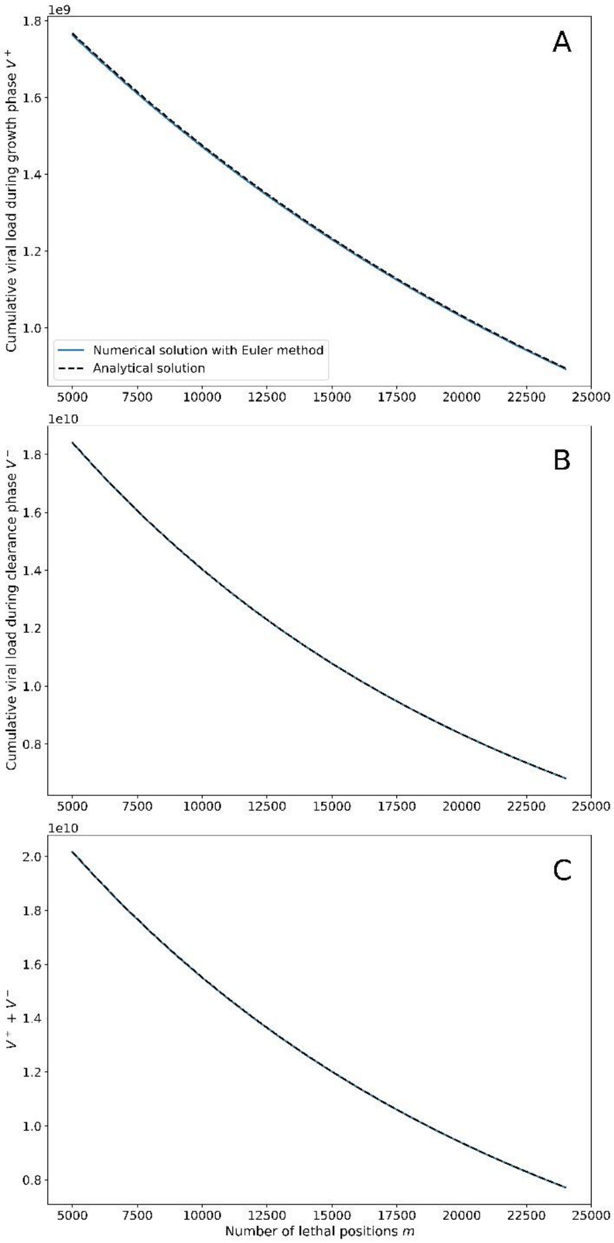

Figure 11: Comparison of analytical expressions from Eqs. 8 (panel A), 11 (panel B) and 12 (panel C) with their numerical values calculated with the Euler method with step size 10^{-4}. Parameters: $b = 7.6$; $a_0 = 3$; $u_0 = u_1 = 10^{-6}$; $a_1 = 8$; $T = 5$; $n = 1$. Initial condition: $x(0) = 1$; $y(0) = 0$.

The next problem is the material that follows equation (18). It's already incorrect (see above). But then the authors say "clearly, yT=vT-xT". It's not obvious why the definition of a new variable merits a "clearly" but also the significance/utility of this new variable is not clear. The expression of interest at the time is Y-, the cumulative mutants after the infection. It is given as
Y- = vT/(a1-b*q1^m) - xT/(a1-b*q1^(m+n))

I think the authors mean to put the expression on common denominator and yT is meant to be the numerator, but since the denominators of the two terms are different, the numerator is not vT-xT. It's (vT-xT)*a1 - b*q1^m*(vT*q1^n-xt). So the meaning of yT is not clear (and again, the expression for Y- is not correct, see above).

The authors then investigate the behaviour of Y-(u1) depending on yT (see note above on yT). Further, yT=vT-xT, and the expressions for the latter two isn't correct as a result of the integration error above. But even putting that aside, the conclusions are not obvious. I played with the expressions a bit and did not see where they came from. And if I didn't see it, many other readers won't either. In my opinion the derivation of these expressions needs to be explained, either here in an SI.

The section you mentioned has now been replaced with the following:

The cumulative virus during the clearance phase with treatment is

$$V^{-} = \frac{v_T}{a_1 - bq_1^m} \tag{16}$$

The cumulative wild-type virus during clearance phase with treatment is

$$X^{-} = \frac{x_T}{a_1 - bq_1^{m+n}} \tag{17}$$

The cumulative mutant virus during clearance phase with treatment is

$$Y^{-} = V^{-} - X^{-} = \frac{v_T}{a_1 - bq_1^m} - \frac{x_T}{a_1 - bq_1^{m+n}} \tag{18}$$

Using $v_T = x_T + y_T$ we write

$$Y^{-} = \frac{x_T + y_T}{a_1 - bq_1^m} - \frac{x_T}{a_1 - bq_1^{m+n}} \tag{19}$$

Introducing $\eta = y_T/x_T$ we write

$$Y^{-} = x_T[\frac{1+\eta}{a_1 - bq_1^m} - \frac{1}{a_1 - bq_1^{m+n}}] \tag{20}$$

From above we have $x_T = \exp[(bq_0^{m+n} - a_0)T]$ and $v_T = \exp[(bq_0^m - a_0)T]$, which in turn specify y_T and η. For the parameters that are relevant to us, we find that Y^{-} as a function of the mutation rate u_1 that is induced during treatment has the following behavior (see **Figure 12**):

1. If $\eta > n/m$ then $Y^{-}(u_1)$ is a declining function. In this case, mutagenic treatment is always beneficial.
2. If $\eta < n/m$ then $Y^{-}(u_1)$ has a single maximum which is attained at

$$u^{*} = \frac{a_1 - b}{mb} \frac{n - \eta m}{n + \eta m} \tag{21}$$

If $u_0 > u^*$ then any mutagenesis treatment is beneficial. If $u_0 < u^*$ then mutagenic treatment needs to be sufficiently strong to be beneficial; specifically we need $Y^-(u_0) > Y^-(u_1)$ where $u_1 > u_0$. For small u_0 the condition $\eta > n/m$ is equivalent to $bT > 1/[mu_0(1-mu_0)]$.

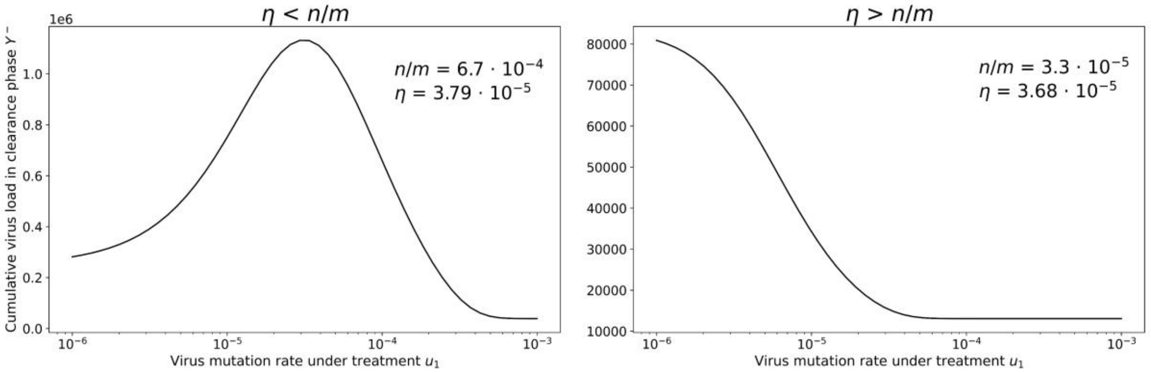

Figure 12: Behavior of the cumulative load of the mutant virus in clearance phase along the mutation rate under treatment u_1. When $\eta < n/m$, the function has a single maximum. When $\eta > n/m$, the function is a declining function along u_1. Parameters: $b = 7.6$, $a_0 = 3$, $u_0 = 10^{-6}$, $T = 5$, $a_1 = 9$. Initial condition: $x_0 = 1$, $y_0 = 0$.

Since I'm not confident yT even is the correct expression to be using giving errors detailed above, this is the point at which I stopped. I think the subject matter is interesting and I hope this can be corrected.

Minor points so far:

1. Equation (8) is an approximation, the authors neglect 1/(bq^m-a0) which is fine assuming that the viral load at time T is large. But why not explain it, it's an extra line, for clarity? I also note that means that many of the expressions that follow starting with V (eq 12) are also approximations, not exact as is indicated.

Thank you for your comment. We have now added an explanation detailing the approximation that led us to Eq. 8. We have also replaced $=$ with $\approx$ where relevant.

2. Then authors examine behaviour of Y-(u1) depending on yT (notes on that above). Authors should remind readers that u1=1-q1 b/c the equation for Y- does not contain u1 and you have to go back two pages to find it.

We have added this clarification as requested, see l.889.

Reviewer #3:

[identifies herself as Pia Abel- zur Wiesch]

The manuscript «Evolutionary safety of death by mutagenesis" is well written and addresses a very important concern regarding the use of the antiviral molnupiravir (lagevrio): that new COVID variants might emerge more easily in treated patients. This is an important and timely contribution- recently, encouraging results of molnupiravir use were published from the Panoramic trial. Moreover, there are only two oral antivirals available. The more widely used paxlovid is difficult to use because of potentially life-threatening interactions in patients receiving multiple other drugs. This limits the use of paxlovid in the most vulnerable groups.

Thank you for your very supportive evaluation.

However, I have two major concerns that need to be addressed before I can recommend publication:

- The authors state that molnupiravir use may be safer in individuals with low clearance. This contrasts findings that new mutants arise more easily with long term infections in immunosuppressed patients (e.g. Weigang et al., https://www.nature.com/articles/s41467-021-26602-3) and these patients may have been the origin of the alpha variant (https://www.nature.com/articles/s41586-021-03291-y). It is not immediately apparent to me how exactly low clearance affects the viral replication rate and therefore mutagenesis, and how these different scenarios were fitted to different patient data. Critically ill patients for example can have very high viral loads, e.g. https://www.atsjournals.org/doi/full/10.1164/rccm.202009-3386LE. If the authors assume that the viral load is constant but just the turnover is low, a reduced viral replication rate also leads to a slower accumulation of mutants. If this is true, it must be corrected (using viral load data from immunosuppressed patients) because it fundamentally alters the conclusion. I would expect then that molnupiravir is safer in patients with an intact immune system.

Thank you for your comment.

You are completely right that new variants of concern arise more easily in immunocompromised individuals with higher viral loads, and who fail to clear the virus within the time frame of 10-15 days typical for healthy individuals.

We admit that this result is counterintuitive, and apologize for the lack of clarity that has led to this misunderstanding. We have now clarified this point, see l. 511 and 513. In particular, we have emphasized that evolutionary safety is always defined relative to the virus load that will be produced if no treatment is administered.

Any infection, also in healthy individuals, will result in a certain load of potentially concerning mutants. Indeed, mutations are always expected to occur, even without mutagenic treatment. The question we explore in this paper is whether mutagenic treatment increases this load beyond what is expected with no treatment.

As you pointed out, immunocompromised individuals can have higher virus loads than healthy individuals. Such patients clear the virus much slower than healthy individuals. Hence, many more replication events occur within an immunocompromised individual compared to a healthy individual. Each replication event is an opportunity for mutation. This might explain why immunocompromised individuals are more likely to be the source of new variants.

Mutagenic treatment can drastically reduce the time to clearance as well as the virus load. Therefore, it reduces the number of replication events in immunocompromised individuals, and thus the cumulative load of mutants produced over the course of an infection.

Let us illustrate this with an extreme example. Suppose that an immunocompromised individual cannot clear the viral infection, and their virus load remains constant along time. Therefore, every day, this individual produces a certain load of mutant virus. A mutagenic treatment can be administered, bringing the within-patient reproduction coefficient of the virus below 1, and hence allow for viral clearance. After treatment, the immunocompromised individual will cease to produce mutant virus. Clearly, in this scenario, the evolutionary safety is very high, and treatment strongly encouraged: without treatment, the cumulative mutant virus load will increase indefinitely. With treatment, the cumulative mutant virus load becomes finite, albeit slightly increased due to additional beneficial mutations.

We hope that this explains why the evolutionary risk factor which we defined is so low for immunocompromised. The viral turnover in immunocompromised individuals is exactly the same as in healthy individuals.

To complete our argument, let us now consider a healthy individual treated with Molnupiravir. A healthy individual has a high clearance rate and is therefore expected to achieve viral clearance without treatment. A mutagenic treatment can decrease their time to clearance. However, this decrease might not counterbalance the negative effect of the increased rate of generation of concerning mutants. Hence, the high ERFs observed for high clearance rates in **Figure 4A** of the main text.

- The authors should clarify which variants their results are valid for. Depending on variant and vaccination status, the time to peak load and clearance can differ https://www.nejm.org/doi/full/10.1056/nejmc2102507. It would be great to obtain sets of parameter estimates for individual variants/vaccine status, and if impossible, this should be stated and a separate sensitivity analysis should be done.

Your point is well taken.

The paper you mentioned provides the times to peak load and clearance for several variants and vaccination statuses. We used these parameter values to conduct a sensitivity analysis for the time to peak virus load and the virus load at peak.

We found that the evolutionary safety factor is robust to variation in the virus load at peak. The evolutionary safety factor becomes higher, in absolute value, the larger the time to

peak of the virus load. This figure is now included in our manuscript as **Supplementary Figure 11**.

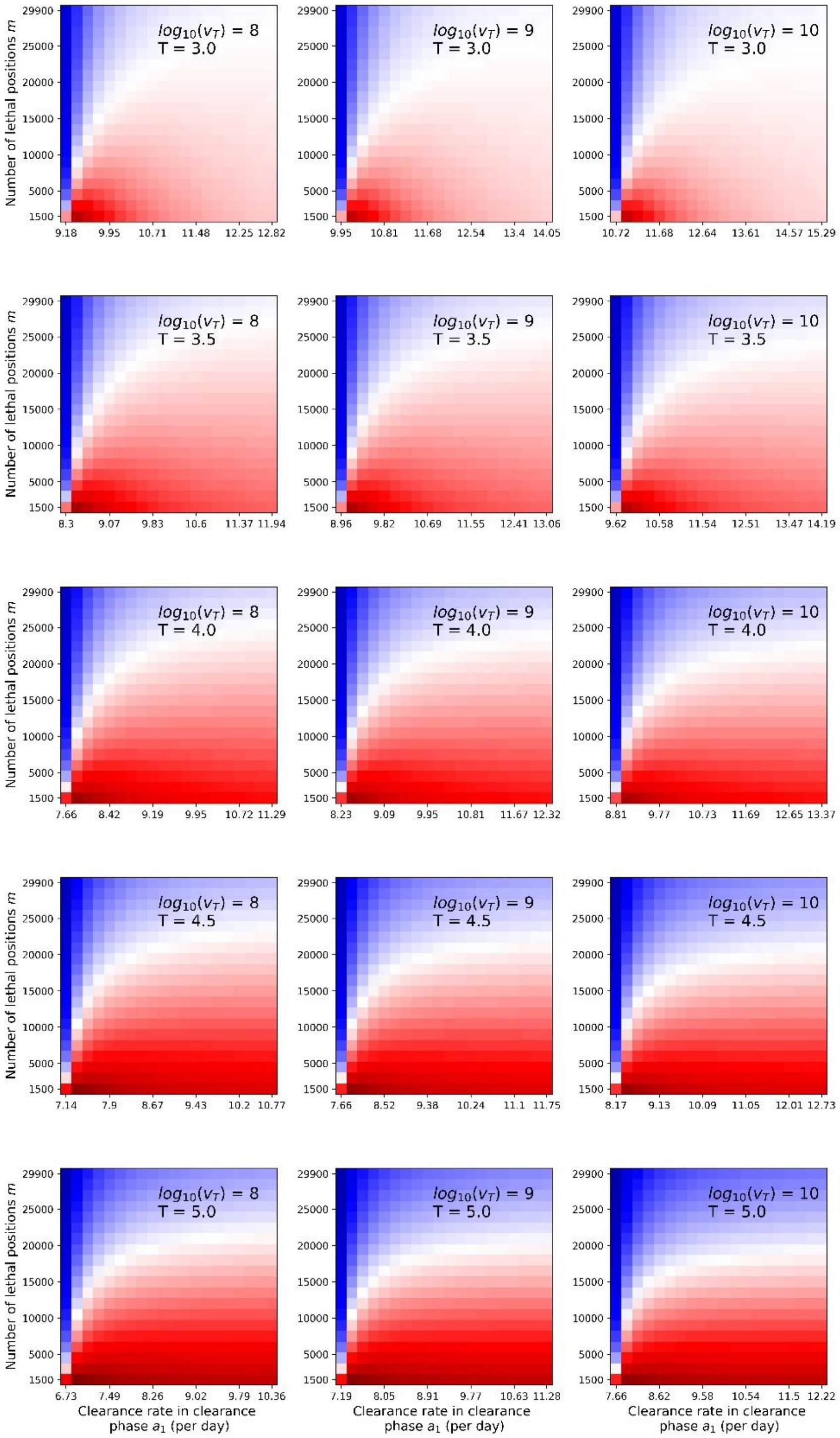

Figure 13: Sensitivity analysis of the evolutionary risk factor on the time to virus peak and the virus load at peak. We calculated the birth rate b for each value of the peak of the virus load and the time to peak of the virus load. For each birth rate b, we adjusted the

clearance rate in the clearance phase a_1 to reflect clearance times between 5 and 30 days. Parameters: $u_0 = 10^{-6}$, $u_1 = 3 \cdot 10^{-6}$, $n = 87$, $a_0 = 3$. Initial conditions: $x(0) = 1$, $y(0) = 0$.

Pia Abel- zur Wiesch

Bibliography

[1] S. Zhou, C.S. Hill, S. Sarkar, L. V. Tse, B.M.D. Woodburn, R.F. Schinazi, T.P. Sheahan, R.S. Baric, M.T. Heise, R. Swanstrom, J. Infect. Dis. 224 (2021) 415–419.

[2] V. Borges, M.J. Alves, M. Amicone, J. Isidro, L. Zé-Zé, S. Duarte, L. Vieira, R. Guiomar, J.P. Gomes, I. Gordo, BioRxiv (2021) 2021.05.19.444774.

[3] Y.M. Bar-On, A. Flamholz, R. Phillips, R. Milo, Elife 9 (2020).

[4] R. Sanjuán, M.R. Nebot, N. Chirico, L.M. Mansky, R. Belshaw, J. Virol. 84 (2010) 9733–9748.

[5] M.B. Schulte, J.A. Draghi, J.B. Plotkin, R. Andino, Elife 4 (2015).

[6] R. Sender, Y.M. Bar-On, S. Gleizer, B. Bernshtein, A. Flamholz, R. Phillips, R. Milo, Proc. Natl. Acad. Sci. 118 (2021).

[7] E. Picardi, C. Manzari, F. Mastropasqua, I. Aiello, A.M. D'Erchia, G. Pesole, Sci. Rep. 5 (2015) 14941.

[8] W.H. Cuddleston, J. Li, X. Fan, A. Kozenkov, M. Lalli, S. Khalique, S. Dracheva, E.A. Mukamel, M.S. Breen, Nat. Commun. 13 (2022) 2997.
