# Supplementary material for: Evolutionary safety of lethal mutagenesis driven by antiviral treatment
Source: PLoS Biol. 2023 Aug 8;21(8):e3002214. doi: 10.1371/journal.pbio.3002214 (PMC10409280; doi:10.1371/journal.pbio.3002214)
Supplement: Attachment — Submitted filename: response_reviewers.pdf [file pbio.3002214.s005.pdf]

Based on the reviews, we are likely to accept this manuscript for publication, provided you satisfactorily address the remaining points raised by reviewer #2. Please also make sure to address the following data and other policy-related requests.

IMPORTANT - please address the following:

a) Your current Title is snappy and intriguing, but a little oblique. Please make it more informative by changing it to: "Evolutionary safety of lethal mutagenesis driven by antiviral drugs"

Thank you for your suggestion. We have changed our title to “Evolutionary safety of lethal mutagenesis driven by antiviral drugs”.

b) Please address the remaining concerns from reviewer #2.

We have now addressed all comments made by reviewer #2.

c) Please provide a blurb, according to the instructions in the submission form.

Our suggestion for a blurb is as follows:

Many antiviral drugs act by increasing the mutation rate of the virus. Could their widespread use accelerate the evolution of new concerning viral variants?

d) Please address my Data Policy requests below; specifically, we need you to supply the code required to generate Figs 2AB, 3AB, 4ABCD, 5AB, S1ABCD, S2, S3, S4AB, S5ABCDEF, S6ABCDEFGHIJ, S7ABCDEFGHIJ, S8ABCDEFGHIJ, S9ABCDEFGHIJ, S10AB, S11, S12, S13AB, S14AB, S15AB, S16AB, S17AB, S18, S19AB, S20AB, S21AB, S22, S23, S24, S25, S26, S27, S28, S29, S30AB, S31; also (in the additional supplement) S2AB, S3, S4, S5, S6, S7, S8ABCDEFGHIJ, S9ABCDEFGHIJ, either as a supplementary data file or as a permanent DOI’d deposition. We note that you mention a Github deposition (https://github.com/gabriela3001/molnupiravir_evol_safety); this looks very comprehensive, but please clarify whether this can indeed allow readers to reproduce all of the Figures.

Thank you for your comment, the code that is needed to reproduce the data, as well as plot all main and supplementary figures, has been deposited in the GitHub repository at https://github.com/gabriela3001/molnupiravir_evol_safety.

e) Because the Github deposition can be changed or deleted at any time, we will need you to make a permanent DOI’d version (e.g. in Zenodo), and to cite this latter URL in the manuscript (see below).

The GitHub repository has the DOI: 10.5281/zenodo.8017992.

f) Please cite the location of the data/code clearly in all relevant main and supplementary Figure legends, e.g. “The data underlying this Figure can be found in https://doi.org/10.5281/zenodo.XXXXX”

Thank you for your comment. We have now added a reference to our GitHub repository under each relevant figure.

As you address these items, please take this last chance to review your reference list to ensure that it is complete and correct. If you have cited papers that have been retracted, please include the rationale for doing so in the manuscript text, or remove these references and replace them with relevant current references. Any changes to the reference list should be mentioned in the cover letter that accompanies your revised manuscript.

References for papers previously cited as preprints that have since been published have now been updated. We ensured that a DOI is given for each cited paper. No additional changes have been made to the reference list.

Regardless of the method selected, please ensure that you provide the code required to generate the following figure panels as they are essential for readers to assess your analysis and to reproduce it: Figs 2AB, 3AB, 4ABCD, 5AB, S1ABCD, S2, S3, S4AB, S5ABCDEF, S6ABCDEFGHIJ, S7ABCDEFGHIJ, S8ABCDEFGHIJ, S9ABCDEFGHIJ, S10AB, S11, S12, S13AB, S14AB, S15AB, S16AB, S17AB, S18, S19AB, S20AB, S21AB, S22, S23, S24, S25, S26, S27, S28, S29, S30AB, S31; also (in the additional supplement) S2AB, S3, S4, S5, S6, S7, S8ABCDEFGHIJ, S9ABCDEFGHIJ. NOTE: the numerical data provided should include all replicates AND the way in which the plotted mean and errors were derived (it should not present only the mean/average values).

Thank you for your comment, the code that is needed to reproduce the data, as well as plot all main and supplementary figures, has been deposited in the GitHub repository at https://github.com/gabriela3001/molnupiravir_evol_safety. The GitHub repository has the DOI: 10.5281/zenodo.8017992.

IMPORTANT: Please also ensure that figure legends in your manuscript include information on where the underlying data can be found, and ensure your supplemental data file/s has a legend.

Please ensure that your Data Statement in the submission system accurately describes where your data can be found.

REVIEWERS' COMMENTS:

Reviewer #2:

In "Evolutionary safety of death by mutagenesis", authors investigate "evolutionary safety" of drugs whose mechanism of action is to induce mutations during viral

replication.

I was asked to examine specifically the mathematics used in this study. As such I began with the Methods (starting after the references, page 59 of my pdf).

Thank you to the authors for their response to my previous questions which addressed the concerns I brought up. I've now been through the rest of the methods and have two recommendations and a question.

The authors thank the reviewer for their previous evaluation, and are pleased to hear that the reviewer's previous concerns were satisfactorily addressed. All additional comments have now been addressed.

Recommendations:
(1) Include an SI with the derivations of the mathematical expressions. I was able to verify most, but not all, of the math. Examples: I don't know how to derive equation (22), and further the parameter regimes aren't clear; when I plug equation (21) into dY-/du I don't recover zero, but it's close for all parameters tested, so I'm guessing it's an approximation. These questions would be clarified with derivations in a separate document. While I'm sure to some top experts such details are unnecessary, if I can't derive them, there are others who won't be able to, either; for reproducibility, in my opinion, these derivations should be included. I don't anticipate this would be too much work for the authors as the calculations are done already.

Thank you for your comment. We have now included the derivation of Eq. 22 all the way from Eq. 15, see **File S2**.

(2) When using approximations, please indicate parameter regimes for which the approximations are valid.

In some places, the authors have done as much (e.g. line 904) but not everywhere (e.g. lines 920, 940, others). I'm sure these seem obvious to the authors! But this is an intriguing and potentially important study being published in a biology journal, so it's likely the mathematical expressions will be used by non-modelers blindly. To minimize the risk of bad science downstream, I urge the authors to put the regimes of validity up front.

Thank you for your comment. We have now added the regimes of validity for our approximations where relevant (see l.920, l.940). In these examples, our approximation assumes that the mutation rate u is much smaller than 1, which is always true.

Finally, my question:

(1) Authors model initiation of immune responses at time T, forcing a peak in the viral load by increasing the clearance rate a so that x'<0 (that is an approximation consistent

with some, though not all, hypotheses on viral load peaks). However, when treatment initiated pre-peak, T is kept as an independent parameter. Would treatment - controlling viral loads and therefore immune stimulation by foreign antigen - not delay immune responses' achieving "full strength"? At the very least should T and treatment time not be correlated in some way? Indeed, treatment may be sufficiently effective so that there is no peak ($bq1^{(m+n)}<a0$), so T is activation of immune responses only. This is the question I was hinting at in my previous review.

The reviewer suggests that the antiviral treatment could have an immunosuppressive effect and delay the time of the peak of the virus load. We explored this hypothetical scenario.

In panel A, we computed the ERF assuming, as we did originally, no delay of the adaptive immunity due to treatment. Here, as assumed throughout the main text, the ERF is the ratio of the cumulative mutant load produced during an infection with treatment to the cumulative mutant load produced without treatment. In both cases (with and without treatment) the peak of the virus load occurs after 5 days.

In panel B, we explore the reviewer's hypothesis that assumes that treatment delays the onset of adaptive immunity, we allowed a delay of a quarter of a day. In this case, the ERF is the ratio of the cumulative mutant load produced during a treatment where the peak of the virus load occurs after 5.25 days and the cumulative mutant load produced without treatment, where the virus load occurs after 5 days.

We observed that the ERF increases when the treatment is immunosuppressive, to be point of being almost always evolutionarily unsafe. However we believe that this extreme situation is unrealistic.

Nevertheless, we have added a sentence referring to this scenario in the Discussion, see l. 547-549.

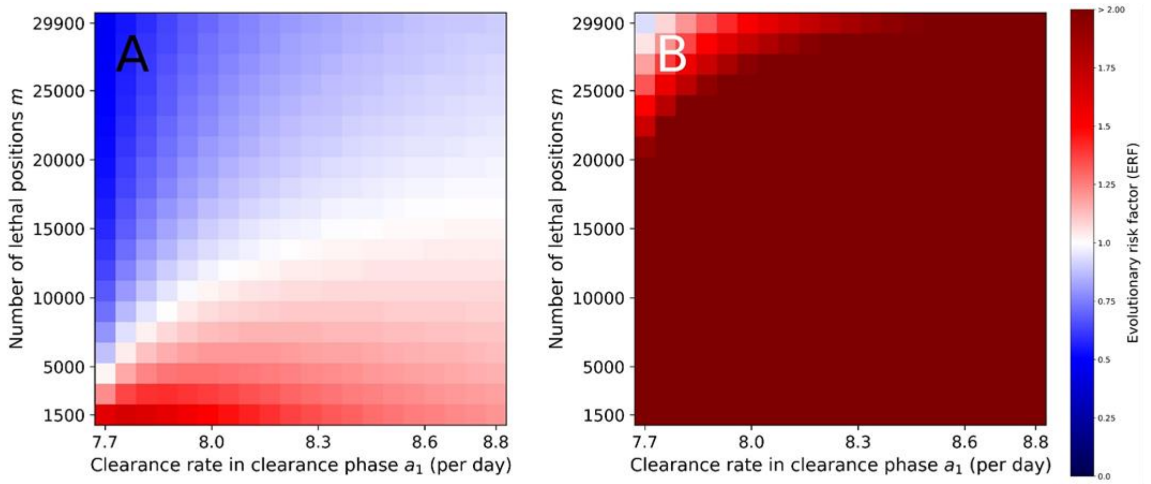

Evolutionary risk factor for immunosuppressive treatment. For each pair of parameters, we numerically compute the ERF for a range of values, while other parameters are fixed. In panel A, treatment does not delay the onset of adaptive immunity, which occurs after 5 days. In panel B, treatment delays the onset of adaptive immunity by a quarter of a day. Treatment starts at infection. Initial condition: $x_0 = 1$ and $y_0 = 0$. Parameters: $b = 7.61$ per day, $a_0 = 3$ per day, $q_0 = 1 - 10^6$, $q_1 = 1 - 3 \cdot 10^{-6}$, $n = 1$. The code used to generate this figure can be found at DOI: 10.5281/zenodo.8017992.

Reviewer #4:

The authors thank you for your positive feedback and sincerely appreciate your kind words and support.

[identifies himself as Raul Andino]

The study conducted by Lobinska, Pilpel and Novak sheds light on the safety and efficacy of antiviral lethal mutagenesis. Their research focuses into the use of nucleoside analogs, a prevalent type of antiviral drugs, that increase the viral mutation rate, causing lethal mutagenesis of the virus. By evaluating the impact of these drugs, the study provides crucial insight into the potential of antiviral lethal mutagenesis as a weapon against viral infections. It presents a comprehensive outlook at the various aspects of this mechanism, including its safety and efficiency, paving the way for future exploration in the field. The results shared in this study will undoubtedly be an essential addition to the existing body of knowledge on antiviral drugs and their application in treating infectious diseases.

As research continues to explore the use of mutagenic treatments for viruses, concerns about their long-term impact on virus evolution have been considered. Namely, their ability to mutate may create an increased number of mutants over time, which could increase virus fitness and pose a safety concern. To address this issue, Lobinska et al have developed a mathematical framework to compare the total mutant load produced with and without mutagenic treatment. This framework considers a variety of variables, such

as timing of treatment and patient immune competence, to predict the rates of viable virus mutants. By using realistic assumptions about viral vulnerability and mutation potential, they provide insight into the potential implications of mutagenic treatments on evolutionary safety.

In the wake of the COVID-19 pandemic, many treatments and drugs have been developed to combat the virus. In particular, Molnupiravir has received FDA approval as a viable treatment option. However, it is crucial to consider the potential evolutionary impact of such drugs. Through extensive analysis, researchers have found that Molnupiravir may be narrowly evolutionarily safe, though this is subject to the current estimate of parameters. To further increase evolutionary safety, restricting treatment with this drug to individuals with a low immunological clearance rate may be beneficial. Additionally, future treatments may be designed to lead to a greater increase in the mutation rate to improve the overall evolutionary impact of these drugs. Consideration of these factors is crucial in developing effective treatments while also taking into account potential long-term consequences. In this interesting study, the authors present a new mathematical rule to help determine the fold-increase in mutation rate necessary for pathogen-treatment combinations to achieve evolutionary safety. The model, which is simple but effective, has far-reaching implications for the development of new treatments against a variety of diseases. This report underscores the importance of mathematical models in the field of medical research and highlights the potential for interdisciplinary collaboration between scientists and mathematicians. With this new tool, we can better understand the complex dynamics of pathogen-treatment interactions and develop more effective treatments to combat evolving pathogens.

The level of effort put forth in addressing the concerns of reviewers is a mark of excellence in academic writing, and the author of this article has clearly exemplified that quality. In addition to the original data presented in the model, the author has provided an in-depth analysis of the data to ensure the validity and comprehensiveness of the article. The additional analyses conducted have helped strengthen the original model and presented a more complete picture of the research topic. The resulting article is well supported and should serve as a valuable resource to researchers and scholars in the field. It is reassuring to see such a thorough approach taken, and it is a testament to the author's dedication and expertise in the field.
